# Synthesis and Structure-Activity Relationships of New 2-Phenoxybenzamides with Antiplasmodial Activity

**DOI:** 10.3390/ph14111109

**Published:** 2021-10-30

**Authors:** Theresa Hermann, Patrick Hochegger, Johanna Dolensky, Werner Seebacher, Eva-Maria Pferschy-Wenzig, Robert Saf, Marcel Kaiser, Pascal Mäser, Robert Weis

**Affiliations:** 1 Institute of Pharmaceutical Sciences, Pharmaceutical Chemistry, University of Graz, Schubertstraße 1, A-8010 Graz, Austria; theresa.hermann@uni-graz.at (T.H.); patrick.hochegger@uni-graz.at (P.H.); johanna.faist@uni-graz.at (J.D.); we.seebacher@uni-graz.at (W.S.); 2 Institute of Pharmaceutical Sciences, Pharmacognosy, University of Graz, Beethovenstraße 8, A-8010 Graz, Austria; eva-maria.wenzig@uni-graz.at; 3 Institute for Chemistry and Technology of Materials (ICTM), Graz University of Technology, Stremayrgasse 9, A-8010 Graz, Austria; robert.saf@tugraz.at; 4 Swiss Tropical and Public Health Institute, Socinstraße 57, CH-4002 Basel, Switzerland; marcel.kaiser@swisstph.ch (M.K.); pascal.maeser@swisstph.ch (P.M.); 5 Swiss TPH, University of Basel, Petersplatz 1, CH-4001 Basel, Switzerland

**Keywords:** antimalarial, CYP3A4 inhibition, PAMPA, 2-phenoxybenzamides, *Plasmodium falciparum*

## Abstract

The 2-phenoxybenzamide **1** from the Medicines for Malaria Venture Malaria Box Project has shown promising multi-stage activity against different strains of *P. falciparum*. It was successfully synthesized via a retrosynthetic approach. Subsequently, twenty-one new derivatives were prepared and tested for their in vitro activity against blood stages of the NF54 strain of *P. falciparum*. Several insights into structure-activity relationships were revealed. The antiplasmodial activity and cytotoxicity of compounds strongly depended on the substitution pattern of the anilino partial structure as well as on the size of substituents. The diaryl ether partial structure had further impacts on the activity. Additionally, several physicochemical and pharmacokinetic parameters were calculated (log P, log D_7_._4_ and ligand efficiency) or determined experimentally (passive permeability and CYP3A4 inhibition). The *tert*-butyl-4-{4-[2-(4-fluorophenoxy)-3-(trifluoromethyl)benzamido]phenyl}piperazine-1-carboxylate possesses high antiplasmodial activity against *P. falciparum* NF54 (*Pf*NF54 IC_50_ = 0.2690 µM) and very low cytotoxicity (L-6 cells IC_50_ = 124.0 µM) resulting in an excellent selectivity index of 460. Compared to the lead structure **1** the antiplasmodial activity was improved as well as the physicochemical and some pharmacokinetic parameters.

## 1. Introduction

Over a half of the world’s population is at risk of an infection with malaria, especially children and pregnant women in developing countries like Africa. In 2019 more than 229 million cases and over 400,000 deaths were reported [1]. Malaria is caused by single-celled, eukaryotic protozoans of the species *Plasmodium*. Five of them are human pathogenic with *Plasmodium falciparum* causing the most deadly and dangerous Malaria tropica [2]. The current gold standard for malaria treatment are artemisinin-based combination therapies (ACTs). They are combinations of short-acting artemisinins with drugs with longer half-life and different mode of action. Progressive resistance development to ACTs in the Southeast Asian region as well as first reports of artemisinin resistances from Africa in 2020, however, present a tremendous threat to previous accomplishments in the fight against malaria [3,4,5,6]. The last chance to at least temporarily prevent resistance development are triple artemisinin-based combination therapies, so called TACTs [7,8]. Vaccine development for malaria is also rather challenging due to the complex life cycle and multiple possible targets. The most advanced candidate in vaccines for *P. falciparum* malaria, RTS,S/AS01, neither provides long term protection nor significant protection against severe malaria [9,10,11]. Therefore, for now, orally administered drugs remain the most important field of research to successfully fight the malaria parasite.

In 2016 the foundation Medicines for Malaria Venture (MMV) has published results of a huge screening project, the so called “Malaria Box” [12]. It consists of 400 compounds with different activity against various strains of *P. falciparum* and serves as starting point for further research. Based on the Malaria Box project, a complex study on resistance development and cross resistances on 50 diverse chemically structured compounds discovered by phenotypic screening was performed [13,14,15]. By comparing the data set of this study, compound **1** was picked as promising lead structure. This 2-phenoxybenzamide is one of few structures exhibiting multi-stage activity against sexual, asexual and liver-stages of *P. falciparum*. Furthermore, in long-term in vitro studies with sub-lethal doses of compound **1** parasites did neither acquire resistances, nor cross-resistances, which is of great significance. 

Within a subsequent study, targets of the 2-phenoxybenzamide were identified [16,17]. Synchronized *P. falciparum* 3D7-A10 parasites with an erythrocytic cycle of 40 h were exposed to different drug concentrations to determine stage specific activity. Compound **1** showed peak activity in sub-micromolar concentrations on late stage trophozoites. Furthermore, dihydroorotate and *N*-carbamoyl-L-aspartate, characteristic metabolic products that indicate disturbance of the mitochondrial electron transport chain, were detected. Consequently, the dihydroorotate-dehydrogenase as well as the cytochrome *bc*_1_ complex are potential targets of **1**. Bloated digestive vacuoles indicate an additional influence on the hemoglobin catabolism [18,19].

The aim of this study was to synthesize new derivatives of the 2-phenoxybenzamide **1** to gain first insights in structure-activity relationships (SAR) and increase antiplasmodial activity. All newly synthesized compounds were tested for their activity against the NF54 strain of *P. falciparum*. To reveal pharmacokinetic parameters essential for orally administered drugs, compounds were analyzed for passive permeability and CYP3A4 inhibition.

## 2. Results and Discussion

### 2.1. Chemistry

**Figure 1:**
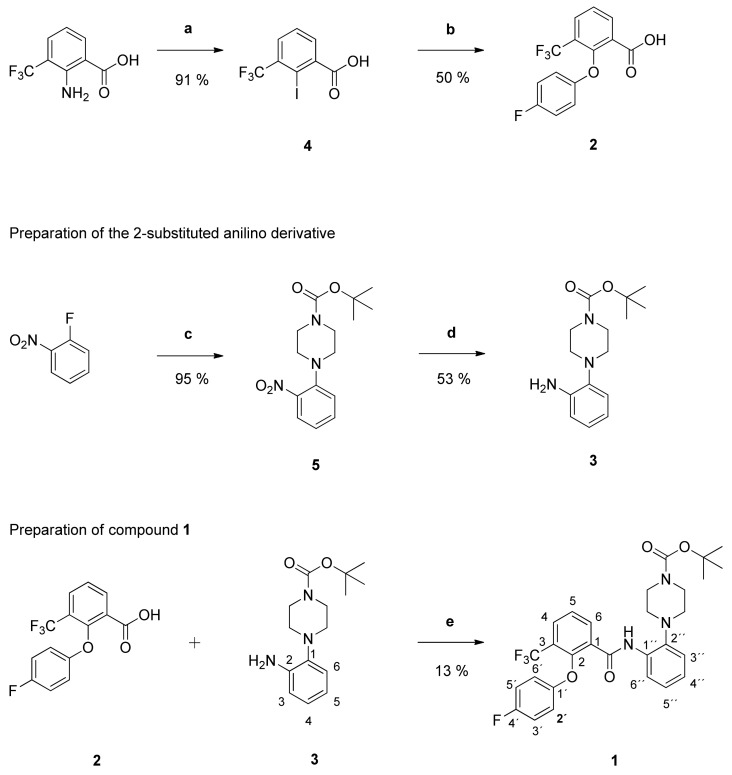
Preparation of compound **1**. Reagents and conditions: (**a**) (1) H_2_SO_4_ 30%, DMSO, 0 °C, 5 min; (2) NaNO_2_, rt, 2 h; (3) KI, H_2_O, rt, 1 h; (4) KI, H_2_O, rt, 1 h; (**b**) 4-fluorophenol, 1,8-diazabicyclo[5.4.0]undec-7-ene, Cu, CuI, pyridine, DMF, 160 °C, 2 h; (**c**) *N*-*Boc*-piperazine, K_2_CO_3_, DMSO, 80 °C, 72 h; (**d**) 15% (m/m) palladium on activated carbon, H_2_, *Me*OH, rt, 24 h; (**e**) (1) CH_2_Cl_2_, 0 °C, 5 min; (2) 2-chloro-*N*-methylpyridinium iodide, diisopropylethylamine, rt, 24 h.

The lead structure **1** was prepared in a multi-step synthesis starting from 3-(trifluoromethyl)anthranilic acid. By this time, no synthetic route to obtain compound **1** has been published. Therefore, a retrosynthetic approach was elaborated to prepare the 2-phenoxy scaffold **2** as well as the 2-substituted derivative of aniline **3**. These partial structures were subsequently coupled to obtain the desired carboxamide **1** (Figure 1).

Treatment of 3-(trifluoromethyl)anthranilic acid with sodium nitrite under acidic conditions yielded the diazonium salt. In the course of a Sandmeyer-like reaction with potassium iodide, the diazonium group was substituted with iodine giving the 2-iodo-3-(trifluoromethyl)benzoic acid **4** in high yields [20]. The iodobenzoic acid **4** was afterwards converted into the diaryl ether **2** by means of a copper-catalyzed Ullmann-like ether synthesis [21]. Thereby, it was coupled with 4-fluorophenol to obtain the 2-phenoxy scaffold **2**. The nucleophilic aromatic substitution of 1-fluoro-2-nitrobenzene with *N*-*Boc*-piperazine and potassium carbonate in dimethyl sulfoxide (DMSO) gave the 1-Boc-4-(2-nitrophenyl)piperazine **5** in high yields [22]. To obtain the 2-substituted derivative of aniline **3**, the nitro group of compound **5** was subsequently reduced with palladium in an atmosphere of hydrogen at a Parr-apparatus [23]. The desired 2-phenoxybenzamide **1** was synthesized by coupling the carboxylic acid **2** with the anilino derivative **3**. Various combinations of DCC, Oxyma Pure, Potassium Oxyma B, EDC x HCl, COMU, CDI and Mukaiyama reagent were used for amide formation. The highest yield was obtained with a combination of 2-chloro-*N*-methylpyridinium iodide (Mukaiyama reagent) and diisopropylethylamine (DIPEA) [24]. This reaction pathway was used for the majority of new compounds in this paper. The successful amide bond formation was detected by significant changes in the NMR spectra. In the ^1^H-NMR spectrum the signal of the aromatic amino protons disappeared and a new signal of the amide hydrogen appeared at much higher frequencies. In the 2D HMBC spectrum we observed a cross-peak from this hydrogen atom to the carbonyl group. 

**Figure 2:**
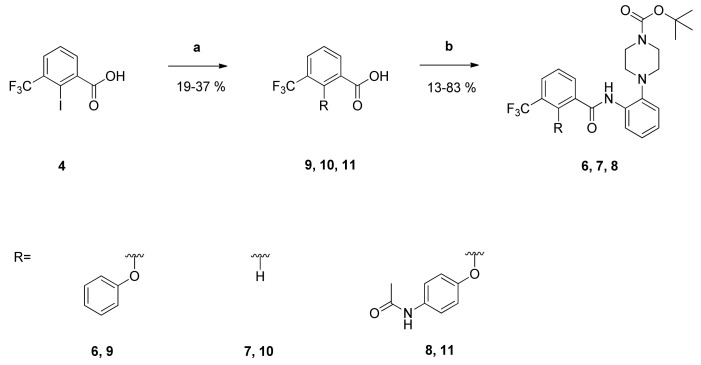
Preparation of compounds **6**, **7** and **8**. Reagents and conditions: (**a**) corresp. phenol, 1,8-diazabicyclo[5.4.0]undec-7-ene, Cu, CuI, pyridine, DMF, 160 °C, 2–48 h; (**b**) (1) anilino derivative **3**, CH_2_Cl_2_, 0 °C, 5 min; (2) 2-chloro-*N*-methylpyridinium iodide, DIPEA, rt, 24–48 h.

To obtain first insights into structure-activity relationships, several series of derivatives were prepared. At first, the 2-(4-fluorophenoxy) substituent was replaced by different functional groups to investigate their influence on the antiplasmodial activity. Compounds **6**, **7** and **8** were synthesized as shown in Figure 2. 

The carboxylic acids **9**, **10** and **11** were prepared from 2-iodo-3-(trifluoromethyl)benzoic acid **4**.The latter was treated with the corresponding phenoles to obtain the diaryl ethers **9** and **11** as well as the 3-(trifluoromethyl)benzoic acid **10**. Subsequent treatment of the carboxylic acids with the anilino derivative **3**, the Mukaiyama reagent and DIPEA in dichloromethane (CH_2_Cl_2_) yielded the amides **6**, **7** and **8**.

In order to determine the importance of the *N*-*Boc*-piperazinyl group for the antiplasmodial activity, the 2-phenoxybenzoic acid **2** was coupled with different primary aromatic amines giving compounds **12**, **13** and **14**.

**Figure 3:**
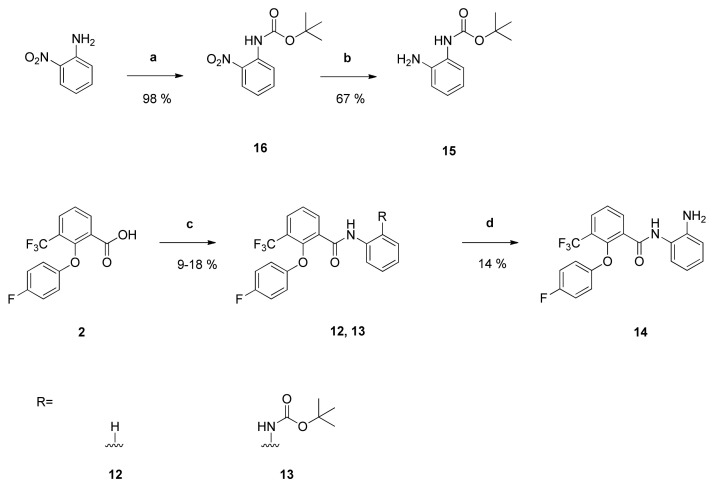
Preparation of compounds **12**, **13** and **14**. Reagents and conditions: (**a**) triethylamine, di-*tert*-butyldicarbonate, CH_2_Cl_2_, rt, 24 h; (**b**) 15% (m/m) palladium on activated carbon, H_2_, *Me*OH, rt, 24 h; (**c**) (1) aniline or compound **15**, CH_2_Cl_2_, 0 °C, 5 min; (2) 2-chloro-*N*-methylpyridinium iodide, DIPEA, rt, 24 h; (**d**) trifluoroacetic acid, CH_2_Cl_2_, rt, 24 h.

The substituted aniline **15** was prepared from 2-nitroaniline. The latter was treated with di-*tert*-butyldicarbonate giving the *tert*-butyl-*N*-(2-nitrophenyl)carbamate **16** [25]. Selective reduction of the nitro group yielded the desired anilino derivative **15**. Reaction of the carboxylic acid **2** with aniline or compound **15** gave benzanilides **12** and **13**, respectively. Carbamate groups are usually cleaved with trifluoroacetic acid in dichloromethane [26]. Such treatment of compound **13** afforded the *N*-(2-aminophenyl)benzamide **14** (Figure 3).

**Figure 4:**
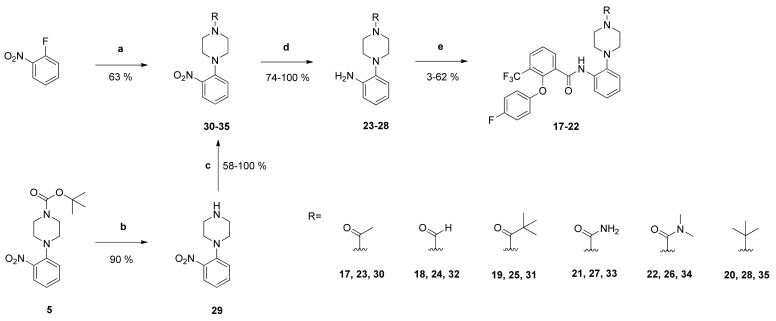
Preparation of compounds **17**–**22**. Reagents and conditions: (**a**) *tert*-butyl-piperazine, K_2_CO_3_, DMSO, 80 °C, 72 h; (**b**) trifluoroacetic acid, CH_2_Cl_2_, rt, 24 h; (**c**) triethylamine, acetyl chloride, acetonitrile, rt, 24 h (compound **30**) or (1) triethylamine, CH_2_Cl_2_, 0 °C, 5 min; (2) pivaloyl chloride, rt, 24 h (compound **31**) or (1) Na, *Et*OH, rt; (2) *Et*OH, 50 °C, 15 min; (3) CHCl_3_, 50 °C, 30 min (compound **32**) or 1*N* HCl, 2*N* KOH, potassium cyanate, rt, 2 h (compound **33**) or (1) triethylamine, 1,1′-carbonyldiimidazole, rt, 30 min; (2) dimethylamine hydrochloride 80 °C, 6 h (compound **34**); (**d**) 15% (m/m) palladium on activated carbon, H_2_, *Me*OH, rt, 24 h; (**e**) (1) carboxylic acid **2**, CH_2_Cl_2_, 0 °C, 5 min; (2) 2-chloro-*N*-methylpyridinium iodide, DIPEA, rt, 24 h.

In another series, the influence of the *tert*-butyloxycarbonyl substituent of the 2-piperazinylphenyl moiety of compound **1** on the antiplasmodial activity was investigated. Its replacement by diverse substituents yielded compounds **17**–**22**. The corresponding primary aromatic amines **23**–**28** for the synthesis of the benzamides **17**–**22** were prepared in multi-stage syntheses from 1-fluoro-2-nitrobenzene (Figure 4).

The *N*-*Boc*-group of carbamate ***5*** was eliminated using trifluoroacetic acid in CH_2_Cl_2_ yielding the 4-unsubstituted piperazinyl derivative **29**. Afterwards, the terminal piperazinyl hydrogen was substituted with different functional groups. Compound **29** was treated with acetyl chloride and pivaloyl chloride, respectively, to obtain the acylated derivatives **30** and **31** [27]. In the course of a Reimer-Tiemann reaction the formylpiperazinyl analogue **32** was prepared from **29** with sodium ethanolate and chloroform [28]. Reaction of **29** with potassium cyanate in a mildly acidic environment yielded the carboxamide **33**. Its *N*,*N*-dimethyl analogue **34** was prepared by reaction of **29** with 1,1′-carbonyldiimidazole and dimethylamine hydrochloride [29,30]. The nitro group of compounds **30**–**34** was reduced with palladium in an atmosphere of hydrogen to obtain the desired 2-substituted anilino derivatives **23**–**27**. Their *tert*-butyl-piperazinyl analogue **28** was prepared by reduction of the nitro group of compound **35**, which was obtained from the reaction of 1-fluoro-2-nitrobenzene with *tert*-butyl-piperazine. The *N*-[2-(piperazin-1-yl)phenyl derivatives **23**–**28** were subsequently coupled with the carboxylic acid **2** to yield the amides **17**–**22** (Figure 4).

In order to evaluate the influence of the *ortho* position of the piperazinyl substituent of **1**, we prepared its 3-substituted and 4-substituted analogues **36** and **37**. Furthermore, the *N*-*Boc* group was replaced by *N*-pivaloyl groups yielding compounds **38** and **39**.

**Figure 5:**
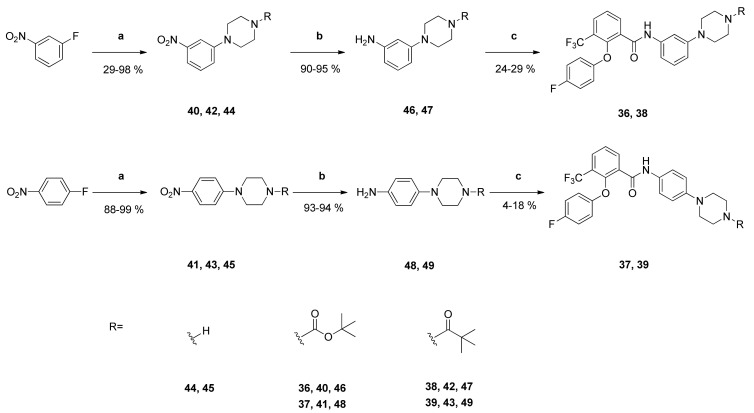
Preparation of compounds **36**–**39**. Reagents and conditions: (**a**) (1) *N*-*Boc*-piperazine, K_2_CO_3_, DMSO, 120 °C, 120 h (compound **40**) or *N*-*Boc*-piperazine, K_2_CO_3_, DMSO, 80 °C, 72 h (compound **41**); (2) trifluoroacetic acid, CH_2_Cl_2_, rt, 24 h (compounds **44** and **45**); (3) pivaloyl chloride, triethylamine, CH_2_Cl_2_, rt, 24 h (compounds **42** and **43**); (**b**) 15% (m/m) palladium on activated carbon, H_2_, *Me*OH, rt, 24 h; (**c**) (1) carboxylic acid **2**, CH_2_Cl_2_, 0 °C, 5 min; (2) 2-chloro-*N*-methylpyridinium iodide, DIPEA, rt, 24 h.

Their syntheses started from the corresponding fluoronitrobenzenes, which reacted in alkaline medium with *N*-*Boc*-piperazine giving compounds **40** and **41**. Their *N*-pivaloyl analogues **42** and **43** were obtained in 2 steps from **40** and **41**. At first the *N*-*Boc* group was cleaved with trifluoroacetic acid affording N-unsubstituted derivatives **44** and **45**. Subsequent reaction with pivaloyl chloride and triethylamine in CH_2_Cl_2_ gave compounds **42** and **43** [27]. The nitro groups of compounds **40**–**43** were reduced with palladium in an atmosphere of hydrogen to obtain their anilino derivatives **46**–**49**. Finally, they were coupled with the carboxylic acid **2** yielding benzamides **36**–**39** (Figure 5).

**Figure 6:**
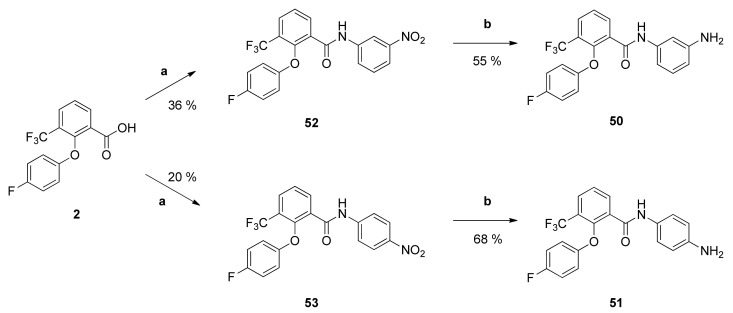
Preparation of compounds **50** and **51**. Reagents and conditions: (**a**) (1) 3-nitroaniline, CH_2_Cl_2_, 0 °C, 5 min; (2) 2-chloro-*N*-methylpyridinium iodide, DIPEA, rt, 48 h (compound **52**) or (1) 4-nitroaniline, CH_2_Cl_2_, 0 °C, 5 min; (2) 2-chloro-*N*-methylpyridinium iodide, DIPEA, rt, 24 h (compound **53**); (**b**) 15% (m/m) palladium on activated carbon, H_2_, *Me*OH, rt, 24 h.

The positive influence of the piperazinyl substituent per se was examined via its replacement by a primary amino group. Compounds **50** and **51** were prepared by amide synthesis of the carboxylic acid **2** with the corresponding aniline giving the *N*-(nitrophenyl)benzamides **52** and **53** which were afterwards reduced with palladium in an atmosphere of hydrogen at the Parr-apparatus yielding the amides **50** and **51** (Figure 6).

**Figure 7:**
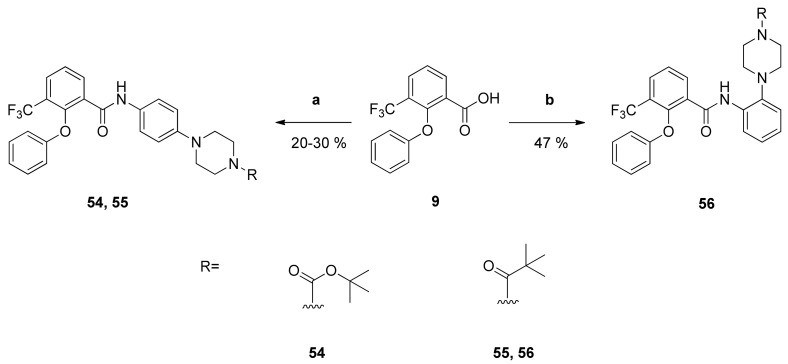
Preparation of compounds **54**–**56**. Reagents and conditions: (**a**) (1) **48** or **49**, CH_2_Cl_2_, 0 °C, 5 min; (2) 2-chloro-*N*-methylpyridinium iodide, DIPEA, rt, 24 h; (**b**) (1) **25**, CH_2_Cl_2_, 0 °C, 5 min; (2) 2-chloro-*N*-methylpyridinium iodide, DIPEA, rt, 24 h.

Finally, analogues **54**, **55** and **56** were synthesized, which exhibit the most promising substituents on the anilino site but lack the 4-fluoro substituent of the phenoxy moiety. They were prepared by reaction of the benzoic acid **9** with anilines **48**, **49** and **25**, respectively (Figure 7).

### 2.2. Antiplasmodial Activity and Cytotoxicity

All newly synthesized compounds were tested for their antiplasmodial activity against the chloroquine-sensitive strain *Plasmodium falciparum* NF54. Cytotoxicity was determined using rat skeletal myofibroblasts (L-6 cells). As standards chloroquine and podophyllotoxin were used. Results obtained are summarized in Table 1.

The already known compound **1** exhibits a 4-fluorophenoxy moiety and a *N*-(2-(4-B*oc*-piperazin-1-yl)phenyl substituent. It served as comparison for all newly synthesized compounds, showing good antiplasmodial activity (*Pf*NF54 IC_50_ = 0.4134 µM) and promising selectivity (S.I. = 316.9). Replacement of the 4-fluorophenoxy substituent by a 4-phenoxy or a 4-acetamidophenoxy distinctly decreased the activity of compounds, but **6** and **8** still exhibited quite good activity and good selectivity (**6**, **8**: *Pf*NF54 IC_50_ = 1.012–1.146 µM; S.I. = 127.1–62.93). Substitution of the 4-fluorophenoxy moiety by a hydrogen atom led to moderate activity and selectivity (**7**: *Pf*NF54 IC_50_ = 3.738 µM; S.I. = 30.22). So the aryloxy substituent appears to be favorable for the antiplasmodial activity. The impact on the cytotoxicity of the above-mentioned compounds was comparatively low (L-6 cells IC_50_ = 73.00–131.0 µM).

Replacement of the piperazinyl substituent in ring position 2 by a hydrogen atom, an amino group or a *N*-*Boc*-amino group caused a decrease of antiplasmodial activity. Compounds **12** and **14** showed negligible activity (*Pf*NF54 IC_50_ = 9.325–21.28 µM) and low to moderate selectivity (S.I. = 21.71–6.080). Their N-Boc-amino analogue **13** exhibited moderate activity (*Pf*NF54 IC_50_ = 1.902 µM), but only low selectivity (S.I. = 9.043), because its cytotoxicity was markedly increased (L-6 cells IC_50_ = 17.20 µM) in contrast to that of compounds **12** and **13** (L-6 cells IC_50_ = 129.4–202.5 µM).

In the next series of compounds the piperazinyl ring in position 2 was retained, but its *N*-*Boc* group was replaced by diverse substituents. The *N*-formyl and the *N*-carbamoyl analogues **18** and **21** possessed weak to negligible activity (*Pf*NF54 IC_50_ = 6.585–15.64 µM), increased cytotoxicity (L-6 cells IC_50_ = 31.80–35.43 µM) and as a result only low selectivity (S.I. = 4.829–2.265). The corresponding *N*,*N*-dimethylcarbamoyl, the *N*-acetyl and the *N*-*tert*-butyl analogues **22**, **17** and **20** showed comparable cytotoxicity (L-6 cells IC_50_ = 20.17–34.72 µM) but slightly improved activity (*Pf*NF54 IC_50_ = 2.300–2.890 µM) and selectivity (S.I. = 10.72–8.770). In this series the *N*-pivaloyl analogues **19** and **56** showed sub-micromolar antiplasmodial activity (*Pf*NF54 IC_50_ = 0.6172–0.6593 µM). Due to their decreased cytotoxicity (L-6 cells IC_50_ = 185.0–190.3 µM) their selectivity indices (S.I. = 299.7–288.6) match up with that of **1**. In this case the 4-fluoro substitution of the phenoxy ring of **19** made no distinction. A benefit of the pivaloyl- compared to the *tert*-butyloxycarbonyl-group is its stability in acidic environment. Bulky, non polar substituents on the terminal piperazinyl nitrogen seem to be beneficial for high antiplasmodial activity.

A shift of the *N*-*Boc* piperazinyl substituent to ring positions 3 and 4 changed the activity significantly, whereas the cytotoxicity of both compounds **36** and **37** remained nearly unchanged (L-6 cells IC_50_ = 124.0 µM). The meta-substituted derivative **36** possessed only moderate activity (*Pf*NF54 IC_50_ = 3.297 µM) and selectivity (S.I. = 37.58). However, its para-substituted analogue **37** showed the highest activity (*Pf*NF54 IC_50_ = 0.2690 µM) and selectivity (S.I. = 461.0) of all tested compounds. Its 2-phenoxy analogue **54** exhibited distinctly lower activity (*Pf*NF54 IC_50_ = 1.222 µM). Due to its decreased cytotoxicity (L-6 cells IC_50_ = 124.0 µM) its selectivity is still good (S.I. = 151.4). Their *N*-pivaloyl analogues **38** and **39** showed slightly increased cytotoxicity (L-6 cells IC_50_ = 78.00–99.62 µM). Again the *meta*-substituted derivative **38** was only moderately active (*Pf*NF54 IC_50_ = 3.174 µM) and selective (S.I. = 24.61). Its para-substituted analogue **39** was a bit more active (*Pf*NF54 IC_50_ = 0.5795 µM) but less selective (S.I. = 171.8) than its ortho-analogues **19** and **56**. Its 2-phenoxy analogue **55** showed remarkably lower activity (*Pf*NF54 IC_50_ = 4.662 µM) and selectivity (S.I. = 19.27), indicating that the 4-fluorophenoxy substituent has generally an advantageous effect. The para substituted *N*-*Boc* and *N*-pivaloylpiperazinyl derivatives are more active than their ortho substituted analogues. As already demonstrated for ortho substituted derivatives a remarkable decrease of activity was observed when the piperazinyl moieties was replaced by amino groups. The 3- amino and 4-amino analogues **50** and **51** were the least active of all tested compounds (*Pf*NF54 IC_50_ = 51.49–51.85 µM).

### 2.3. Physicochemical and Pharmacokinetic Properties

In addition to antiplasmodial activity and cytotoxicity of compounds **1**, **6**–**8**, **12**–**14**, **17**–**22**, **36**–**39**, **50**, **51** and **54**–**56**, some physicochemical parameters like log P and log D_7_._4_ were calculated. Furthermore, ligand efficiency (LE) was determined (Table 2) [31]. The log P and log D_7_._4_ values of compounds range between 4.43–6.60. Among the compounds with considerable antiplasmodial activity the *N*-[4-(4-pivaloylpiperazinyl)phenyl] benzamide **39** (log P = 5.56) exhibits the lowest log P and log D_7_._4_ values, which is compared to compound **1** (log P = 6.44) a remarkable improvement.

Ligand efficiency is an important parameter in early drug development. It becomes more apparent, that large molecules often have disadvantageous molecular properties when it comes to oral bioavailability. Ligand efficiency is defined by the free binding energy for a compound divided by its number of heavy atoms (HA). The calculated values ranged from 0.183–0.255 kcal/mol/HA. From the group of more active compounds it was again **39** (LE = 0.236 kcal/mol/HA) that showed the highest value, which is a minor enhancement compared to **1** (LE = 0.214 kcal/mol/HA).

In addition, ADME assays to determine pharmacokinetic parameters were performed (Table 3). Passive permeability and inhibition of Cytochrom P450 3A4 were determined. Permeability of compounds through a semipermeable membrane was detectable for all compounds except **22** due to insufficient solubility in the used solvents. The most active compounds **19**, **37**, **39** and **56** showed quite low permeability (P_e_ = 0.09–0.24 × 10^−6^ cm/s). In the group of compounds with quite good activity the 2-phenyl and the 2-(4-acetamidophenyl) derivative **6** and **8** (P_e_ = 4.06–3.00 × 10^−6^ cm/s) possessed improved passive permeability compared to their 2-(4-fluorophenyl) analogue **1** (P_e_ = 2.37 × 10^−6^ cm/s). In general, compounds with permeabilities higher than 1.5 × 10^−6^ cm/s are considered to be highly permeable.

Inhibition of the phase I liver enzyme Cytochrom P450 3A4 that plays a crucial role in drug metabolism was determined for compounds with the highest antiplasmodial activities. CYP3A4 inhibition of compounds could result in increased bioavailability of simultaneously applied drugs. The lead structure **1** exhibits high enzyme interaction (87%) that is however surpassed by most tested compounds. Only compounds **18** (82%), **54** (80%) and **55** (60%) show less inhibition of CYP3A4.

## 3. Materials and Methods

### 3.1. Instrumentation and Chemicals

Melting points were obtained on an Electrothermal IA 9200 melting point apparatus. IR-spectra were acquired by a Bruker Alpha Platinum ATR FTIR spectrometer (KBr discs), the frequencies are reported in cm^−1^. The structures of all newly synthesized compounds were determined by one- and two-dimensional NMR spectroscopy. NMR spectra: Varian UnityInova 400 MHz and Bruker Avance Neo 400 MHz, 5 mm tubes, TMS as internal standard. Shifts in ^1^H NMR (400 MHz) and ^13^C NMR (100 MHz) spectra are reported in ppm; ^1^H- and ^13^C-resonances were assigned using ^1^H,^1^H- and ^1^H,^13^C-correlation spectra and are numbered as given in Figure 1. Signal multiplicities are abbreviated as follows: br, broad; d, doublet; dd, doublet of doublets; ddd, doublet of doublet of doublets; dt, doublet of triplets; m, multiplet; q, quartet; t, triplet; td, triplet of doublets; s, singlet. HRMS: Micromass Tofspec 3E spectrometer (MALDI) and GCT-Premiere, Waters (EI, 70 eV) and Q Exactive Hybrid Quadrupole-Orbitrap mass spectrometer (Thermo Fisher Scientific, Waltham, MA, USA).

Materials: column chromatography (CC): silica gel 60 (Merck 70–230 mesh, pore diameter 60 Å), flash silica gel 60 (Merck 230–400 mesh, pore diameter 60 Å or VWR 230–400 mesh, pore diameter 60 Å); thin-layer chromatography (TLC): TLC plates silica gel 60 F254 (Merck); PAMPA: 96-well precoated Corning Gentest PAMPA plate (Corning, Glendale, AZ, USA), 96-well UV-Star Microplates (Greiner Bio-One, Kremsmünster, Austria), SpectraMax M3 UV plate reader (Molecular Devices, San Jose, CA, USA); CYP3A4 inhibition assay: P450-Glo CYP3A4 Assay with Luciferin-IPA, NADPH Regeneration System and Beetle Luciferin, Potassium Salt (Promega Corporation, Madison, WI, USA), Corning Supersomes Human CYP3A4 + Oxidoreductase + b5 and Corning Supersomes Human P450 Oxidoreductase + b5 Negative Control (Corning, Glendale, AZ, USA), Ketoconazole Pharmaceutical Secondary Standard (Sigma Aldrich), 96-well White Plate (Greiner Bio-One, Kremsmünster, Austria); SpectraMax M3 plate reader (Molecular Devices, San Jose, CA, USA). ^1^H-NMR and ^13^C-NMR spectra of new compounds are available in Supplementary Materials Section (Figures S1–S40).

### 3.2. Syntheses

2-Iodo-3-(trifluoromethyl)benzoic acid (**4**): 3-(Trifluoromethyl)anthranilic acid (2.11 g (10.33 mmol)) was dissolved in dimethylsulfoxide (17 mL) and the solution was ice-cooled. Sulfuric acid 30 percent (17 mL) was added and the reaction mixture was stirred at 0 °C for 5 min. After that, NaNO_2_ (1.54 g (22.35 mmol)) was added, the ice bath was removed and the reaction mixture was stirred at room temperature for 2 h. KI (3.02 g (18.21 mmol)) was dissolved in water (10 mL) and added dropwise with a syringe through a septum. The reaction mixture was stirred at ambient temperature for 1 h. After that, the second portion of KI (1.71 g (10.33 mmol)) dissolved in water (7 mL) was added and the reaction mixture was stirred for another hour at room temperature. Then, ethyl acetate (50 mL) were added. The aqueous and organic phases were separated. The organic phase was washed with water and brine, dried over anhydrous sodium sulfate and filtered. The solvent was evaporated in vacuo. The residue was recrystallized from water, giving compound **4** as brownish solid (2.97 g (91%)). m.P. 134 °C. NMR data were in accordance with literature data [32].

#### 3.2.1. General Procedure for the Synthesis of Compounds **2**, **9**, **10** and **11**

The corresponding iodobenzoic acid derivative (4.00 mmol) was dissolved in dry dimethylformamide. Phenol (4.20 mmol), catalytic amounts of copper (0.53 mmol) and copper (I) iodide (0.18 mmol), 1,8-diazabicyclo[5.4.0]undec-7-ene (12.00 mmol) and dry pyridine (0.80 mmol) were added. The reaction mixture was refluxed at 160 °C for 2–48 h. Then, the mixture was acidified with 2*N* HCl to a pH of 1. Ice and dichloromethane were added. The aqueous and organic phases were separated. The aqueous phase was extracted three times with dichloromethane. The combined organic phases were washed with water and brine, dried over anhydrous sodium sulfate and filtered. The solvent was evaporated in vacuo yielding the raw diaryl ether, which was purified by column chromatography.

2-(4-Fluorophenoxy)-3-(trifluoromethyl)benzoic acid (**2**): The reaction of compound **4** (1.50 g (4.74 mmol)), 4-fluorophenol (561 mg (4.98 mmol)), copper (40 mg (0.62 mmol)), copper (I) iodide (43 mg (0.23 mmol)), DBU (2.17 g (14.23 mmol)) and dry pyridine (75 mg (0.95 mmol)) in dry dimethylformamide (38 mL) gave the raw diaryl ether. It was purified by column chromatography (silica gel, CH_2_Cl_2/_*Me*OH/AcOH 149:1:1) followed by recrystallization from CH_2_Cl_2_ yielding compound **2** as white solid (711 mg (50%)). m.P. 143 °C. IR = 3424, 1703, 1503, 1452, 1321, 1249, 1218, 1137, 827, 784, 678; ^1^H NMR (CDCl_3_, 400 MHz) *δ* = 6.67–6.70 (m, 2H, 2′-H, 6′-H), 6.90–6.94 (m, 2H, 3′-H, 5′-H), 7.44 (t, *J* = 7.8 Hz, 1H, 5-H), 7.96 (dd, *J* = 7.6, 1.7 Hz, 1H, 4-H), 8.18 (dd, *J* = 7.9, 1.7Hz, 1H, 6-H); ^13^C NMR (CDCl_3_, 100 MHz) *δ* = 115.87 (d, *J* = 22.8 Hz, C-3′, C-5′), 116.42 (d, *J* = 7.7 Hz, C-2′, C-6′), 122.59 (q, *J* = 273 Hz, CF_3_), 125.04 (C-1), 125.12 (C-5), 126.21 (q, *J* = 31.1 Hz, C-3), 132.33 (q, *J* = 4.6 Hz, C-4), 136.44 (C-6), 153.25 (q, *J* = 1.8 Hz, C-2), 155.05 (d, *J* = 2.5 Hz, C-1′), 158.03 (d, *J* = 240 Hz, C-4′), 168.79 (C=O); HRMS (EI+) calcd for C_14_H_8_F_4_O_3_ [M^+^]: 300.0410; found: 300.0406.

2-Phenoxy-3-(trifluoromethyl)benzoic acid (**9**): The reaction of compound **4** (1.63 g (5.15 mmol)), phenol (509 mg (5.41 mmol)), copper (49 mg (0.77 mmol)), copper (I) iodide (54 mg (0.28 mmol)), DBU (2.35 g (15.45 mmol)) and dry pyridine (71 mg (0.90 mmol)) in dry dimethylformamide (45 mL) gave the raw diaryl ether. It was purified by column chromatography (silica gel, CH_2_Cl_2_/isopropyl alcohol/NH_3_ cc. 8:9:2). The residue was dissolved in water (10 mL) and acidified with 2*N* HCl to a pH of 1. The aqueous phase was extracted with dichloromethane. The organic phase was dried over anhydrous sodium sulfate, filtered and the solvent was evaporated in vacuo yielding compound **9** as pale brown solid (538 mg (37%)). IR = 3430, 1684, 1601, 1493, 1451, 1322, 1244, 1169, 1132, 750; ^1^H NMR (CDCl_3_, 400 MHz) *δ* = 6.76 (d, *J* = 8.1 Hz, 2H, 2 ′-H, 6 ′-H), 7.01 (t, *J* = 7.4 Hz, 1H, 4′-H), 7.24 (d, *J* = 8.1 Hz, 2H, 3′-H, 5′-H), 7.44 (t, *J* = 7.9 Hz, 1H, 5-H), 7.94 (d, *J* = 7.8 Hz, 1H, 4-H), 8.20 (d, *J* = 7.7 Hz, 1H, 6-H); ^13^C NMR (CDCl_3_, 100 MHz) *δ* = 115.33 (C-2′, C-6′), 122.49 (C-4′), 122.62 (q, *J* = 273 Hz, CF_3_), 125.01 (C-5), 125.23 (C-1), 126.23 (q, *J* = 31.7 Hz, C-3), 129.44 (C-3′, C-5′), 132.25 (q, *J* = 5.2 Hz, C-4), 136.33 (C-6), 153.03 (C-2), 158.94 (C-1′), 167.96 (C=O); HRMS (ESI -) calcd for C_14_H_8_F_3_O_3_ [M-H]^−^: 281.0426; found: 281.0426.

3-(Trifluoromethyl)benzoic acid (**10**): The reaction of compound **4** (1.28 g (4.06 mmol)), 2-nitrophenol (545 mg (3.92 mmol)), copper (34 mg (0.54 mmol)), copper (I) iodide (42 mg (0.22 mmol)), DBU (1.83 g (12.00 mmol)) and dry pyridine (63 mg (0.80 mmol)) in dry dimethylformamide (26 mL) gave the raw benzoic acid. It was purified by column chromatography (silica gel, CH_2_Cl_2_/*Me*OH/AcOH 59:1:1) yielding compound **10** as brownish solid (277 mg (19%)). NMR data were in accordance with literature data [33].

2-(4-Acetamidophenoxy)-3-(trifluoromethyl)benzoic acid (**11**): The reaction of compound **4** (1.27 g (4.03 mmol)), *N*-(4-hydroxyphenyl)acetamide (645 mg (4.27 mmol)), copper (35 mg (0.55 mmol)), copper (I) iodide (43 mg (0.25 mmol)), DBU (1.82 g (12.00 mmol)) and dry pyridine (63 mg (0.80 mmol)) in dry dimethylformamide (30 mL) for 48 h gave the raw diaryl ether. It was purified by column chromatography (silica gel, CH_2_Cl_2_/*Et*OH/AcOH 9:1:0.1) yielding compound **11** as pale-yellow solid (438 mg (32%)). IR = 3430, 2925, 1706, 1634, 1507, 1453, 1322, 1242, 1135, 672; ^1^H NMR (*Me*OD, 400 MHz) *δ* = 2.11 (s, 3H, CH_3_), 6.72–6.75 (m, 2H, 2′-H, 6′-H), 7.42–7.45 (m, 2H, 3′-H, 5′-H), 7.50 (t, *J* = 7.8 Hz, 1H, 5-H), 7.94 (dd, *J* = 7.9, 1.6 Hz, 1H, 4-H), 8.09 (dd, *J* = 7.8, 1.7 Hz, 1H, 6-H); ^13^C NMR (*Me*OD, 100 MHz) *δ* = 23.89 (CH_3_), 117.09 (C-2′, C-6′), 122.87 (C-3′, C-5′), 124.83 (q, *J* = 272 Hz, CF_3_), 126.51 (C-5), 126.56 (q, *J* = 30.8 Hz, C-3), 131.42 (q, *J* = 5.0 Hz, C-4), 131.61 (C-1), 134.68 (C-4′), 136.90 (C-6), 153.30 (q, *J* = 1.8 Hz, C-2), 157.20 (C-1′), 169.11 (COOH), 171.71 (C=O); HRMS (ESI +) calcd for C_16_H_13_F_3_NO_4_ [M+H]^+^: 340.0797; found: 340.0793.

#### 3.2.2. General Procedure for the Synthesis of Compounds **5**, **35**, **40** and **41**

Potassium carbonate (14.00 mmol) and the corresponding piperazine derivative (14.00 mmol) were suspended in dry dimethyl sulfoxide. The corresponding fluoronitrobenzene (7.00 mmol) was added and the suspension was refluxed at 80–120 °C for 72–120 h. After that, the reaction mixture was diluted with diethyl ether (30 mL) and acidified with 2*N* HCl to a pH of 1. The aqueous and organic phases were separated. The aqueous phase was extracted three times with diethyl ether. The combined organic phases were washed with water and brine, dried over anhydrous sodium sulfate and filtered. The solvent was evaporated in vacuo yielding the raw nitro compound, which was either purified by column chromatography or used without further purification.

*tert*-Butyl-4-(2-nitrophenyl)piperazine-1-carboxylate (**5**): The reaction of potassium carbonate (1.96 g (14.20 mmol), *N*-*Boc*-piperazine (2.64 g (14.20 mmol) and 1-fluoro-2-nitrobenzene (1.00 g (7.10 mmol)) in dry DMSO (40 mL) yielded compound **5** as orange oil (2.07 g (95%)) which was used without further purification. NMR data were in accordance with literature data [23].

*tert*-Butyl-4-(2-nitrophenyl)piperazine (**35**): The reaction of potassium carbonate (1.11 g (8.00 mmol), 1-*tert*-butylpiperazine (680 mg (4.57 mmol) and 1-fluoro-2-nitrobenzene (565 mg (4.00 mmol)) in dry DMSO (23 mL) gave the raw product. It was purified by column chromatography (silica gel, CH_2_Cl_2/_*Me*OH 19:1) yielding compound **35** as orange oil (664 mg (63%)). IR = 2977, 2830, 1604, 1527, 1490, 1447, 1353, 1293, 1222, 1134, 970, 849, 776, 757; ^1^H NMR (CDCl_3_, 400 MHz) *δ* = 1.11 (s, 9H, (CH_3_)_3_), 2.71–2.75 (m, 4H, N(CH_2_)_2_), 3.07–3.10 (m, 4H, N(CH_2_)_2_), 7.01 (td, *J* = 7.7, 1.2 Hz, 1H, 4-H), 7.14 (dd, *J* = 8.3, 1.1 Hz, 1H, 6-H), 7.46 (td, *J* = 7.8, 1.6 Hz, 1H, 5-H), 7.74 (dd, *J* = 8.1, 1.6 Hz, 1H, 3-H); ^13^C NMR (CDCl_3_, 100 MHz) δ = 25.93 ((CH_3_)_3_), 45.76 (N(CH_2_)_2_), 52.26 (N(CH_2_)_2_), 53.83 (C*Me*_3_),120.70 (C-6), 121.40 (C-4), 125.82 (C-3), 133.41 (C-5), 143.27 (C-2), 146.01 (C-1); HRMS (ESI +) calcd for C_14_H_22_N_3_O_2_ [M+H]^+^: 264.1712; found: 264.1712.

*tert*-Butyl-4-(3-nitrophenyl)piperazine-1-carboxylate (**40**): Refluxing a suspension of potassium carbonate (1.94 g (14.04 mmol), *N*-*Boc*-piperazine (2.61 g (14.00 mmol) and 1-fluoro-3-nitrobenzene (988 mg (7.00 mmol)) in dry DMSO (40 mL) at 120 °C for 120 h gave the raw product. It was purified by column chromatography (silica gel, cyclohexane (CH)/ethyl acetate (EtAc) 4:1) yielding compound **40** as orange solid (624 mg (29%)). NMR data were in accordance with literature data [34].

*tert*-Butyl-4-(4-nitrophenyl)piperazine-1-carboxylate (**41**): The reaction of potassium carbonate (1.94 g (14.02 mmol), *N*-*Boc*-piperazine (2.69 g (14.44 mmol) and 1-fluoro-4-nitrobenzene (988 mg (7.00 mmol)) in dry DMSO (40 mL) yielded compound **41** as orange solid (2.07 g (96%)) which was used without further purification. NMR data were in accordance with literature data [35].

*tert*-Butyl-*N*-(2-nitrophenyl)carbamate (**16**): To a solution of 2-nitroaniline (569 mg (4.12 mmol)) in dry CH_2_Cl_2_ (18 mL), dry triethylamine (567 mg (5.60 mmol)) was added. After that, di-*tert*-butyldicarbonat was added in portions. The reaction mixture was stirred at room temperature for 24 h. Then, the organic phase was washed with 8% aq NaHCO_3_ and brine, dried over anhydrous sodium sulfate and filtered. The solvent was evaporated in vacuo yielding compound **16** as orange solid (962 mg (98%)), which was used without further purification. NMR data were in accordance with literature data [36].

#### 3.2.3. General Procedure for the Synthesis of Compounds **14**, **29**, **44** and **45**

To an ice-cooled solution of the corresponding *N*-*Boc* derivative (1.00 mmol) in dry CH_2_Cl_2_ (10 mL) a solution of trifluoroacetic acid (30 mmol) in dry CH_2_Cl_2_ (3 mL) was added dropwise via a dropping funnel. The ice-bath was removed and the reaction mixture was stirred at room temperature for 24 h. After that, the solvent and excess trifluoroacetic acid were evaporated in vacuo. The residue was suspended in a solution of potassium carbonate (6.00 mmol) in water (12 mL). The aqueous phase was extracted five times with CH_2_Cl_2*/*_isopropyl alcohol (3:1). The organic phases were combined, dried over anhydrous sodium sulfate and filtered. The solvent was evaporated in vacuo yielding the amino or piperazine derivative, which was either purified by column chromatography or used without further purification.

*N*-(2-Aminophenyl)-2-(4-fluorophenoxy)-3-(trifluoromethyl)benzamide (**14**): Reaction of compound **13** (502 mg (1.02 mmol)) with trifluoroacetic acid (3.49 g (30.64 mmol)) in dichloromethane (13 mL) gave the protonated form **14**. Work-up with a solution of potassium carbonate (2.92 g (21.00 mmol)) in water (42 mL) gave the raw product. It was purified by column chromatography (silica gel, CH_2_Cl_2/_EtAc 39:1) yielding compound **14** as white solid (56 mg (14%)). IR = 3292, 1649, 1501, 1452, 1312, 1223, 1141, 1099, 778, 743, 685; ^1^H NMR (CDCl_3_, 400 MHz) *δ* = 3.50 (br s, 2H, NH_2_), 6.69–6.74 (m, 2H, 3″-H, 5″-H), 6.79–6.83 (m, 2H, 2′-H, 6′-H), 6.92 (dd, *J* = 8.3, 1.5 Hz, 1H, 6″-H), 6.95–6.98, (m, 2H, 3′-H, 5′-H), 7.01 (td, *J* = 7.7, 1.5 Hz, 1H, 4″-H), 7.54 (t, *J* = 7.8 Hz, 1H, 5-H), 7.91 (dd, *J* = 8.0, 1.8 Hz, 1H, 4-H), 8.33 (dd, *J* = 7.9, 1.8 Hz, 1H, 6-H), 8.37 (br s, 1H, NH); ^13^C NMR (CDCl_3_, 100 MHz) *δ* = 116.26 (d, *J* = 8.3 Hz, C-2′, C-6′), 116.62 (d, *J* = 23.7 Hz, C-3′, C- 5′), 117.70 (C-3″), 119.31 (C-5″), 122.64 (q, *J* = 274 Hz, CF_3_), 123.12 (C-1″), 125.40 (C-6″), 125.41 (q, *J* = 31.8 Hz, C-3), 126.33 (C-5), 127.64 (C-4″), 130.54 (C-1), 130.88 (q, *J* = 4.9 Hz, C-4), 136.00 (C-6), 140.88 (C-2″), 149.50 (q, *J* = 1.9 Hz, C-2), 153.99 (d, *J* = 2.5 Hz, C-1′), 158.53 (d, *J* = 242 Hz, C-4′), 162.22 (C=O); HRMS (ESI +) calcd for C_20_H_15_F_4_N_2_O_2_ [M+H]^+^: 391.1070; found: 391.1060.

1-(2-Nitrophenyl)piperazine (**29**): Reaction of compound **5** (2.24 g (7.30 mmol)) with trifluoroacetic acid (5.00 g (43.80 mmol)) in dichloromethane (95 mL) gave the protonated form of **29**. Work-up with a solution of potassium carbonate (6.06 g (43.80 mmol)) in water (88 mL) yielded compound **29** as orange oil (1.36 g (90%)), which was used without further purification. NMR data were in accordance with literature data [37].

1-(3-Nitrophenyl)piperazine (**44**): Reaction of compound **40** (464 mg (1.51 mmol)) with trifluoroacetic acid (2.05 g (18.00 mmol)) in dichloromethane (20 mL) gave the protonated form of **44**. Work-up with a solution of potassium carbonate (1.25 g (9.00 mmol)) in water (18 mL) yielded compound **44** as orange oil (307 mg (98%)), which was used without further purification. NMR data were in accordance with literature data [34].

1-(4-Nitrophenyl)piperazine (**45**): Reaction of compound **41** (1.02 g (3.33 mmol)) with trifluoroacetic acid (4.45 g (39.00 mmol)) in dichloromethane (42 mL) gave the protonated form of **45**. Work-up with a solution of potassium carbonate (2.70 g (19.56 mmol)) in water (40 mL) yielded compound **45** as yellow solid (683 mg (99%)), which was used without further purification. NMR data were in accordance with literature data [38].

1-[4-(2-Nitrophenyl)piperazin-1-yl]ethan-1-one (**30**): To a solution of compound **29** (1.36 g (6.59 mmol)) in dry acetonitrile (27 mL), dry triethylamine was added (2.00 g (19.78 mmol)). Acetyl chloride (1.55 g (19.78 mmol)) was added dropwise with a syringe through a septum. The reaction mixture was stirred at room temperature for 24 h. Afterwards, the solvent was evaporated in vacuo. The residue was dissolved in CH_2_Cl_2_ (20 mL). The organic phase was washed with 8% aq NaHCO_3_ and brine, dried over anhydrous sodium sulfate and filtered. The solvent was evaporated in vacuo yielding compound **30** as brown oil (1.64 g (100%)), which was used without further purification. NMR data were in accordance with literature data [23].

4-(2-Nitrophenyl)piperazine-1-carbaldehyde (**32**): Sodium (498 mg (21.70 mmol)) was added in portions to dry ethanol (12 mL). After that, a solution of compound **29** (746 mg (3.60 mmol)) in dry ethanol (5 mL) was added. The reaction mixture was stirred at 50 °C for 15 min. Dry chloroform (1.55 g (13.02 mmol)) was added dropwise with a syringe through a septum. The reaction mixture was stirred for another 45 min at 50 °C. Then, the mixture was quenched with water (30 mL). The aqueous and organic phases were separated, and the aqueous phase was extracted with CH_2_Cl_2_. The combined organic phases were washed with 1*N* HCl, dried over anhydrous sodium sulfate and filtered. The solvent was evaporated in vacuo yielding compound **32** as brown oil (661 mg (78%)), which was used without further purification. NMR data are in accordance with literature data [39].

4-(2-Nitrophenyl)piperazine-1-carboxamide (**33**): To a solution of **29** (559 mg (2.73 mmol)) in 1*N* HCl (3.5 mL), 2*N* KOH was added dropwise up to a pH of 3. After that, potassium cyanate (292 mg (3.60 mmol)) was added, and the reaction mixture was stirred at room temperature for 2 h. The precipitate was filtered and washed with water. It was dissolved in CH_2_Cl_2_. The organic phase was washed with 2*N* NaOH, dried over anhydrous sodium sulfate and filtered. The solvent was evaporated in vacuo yielding compound **33** as orange solid (396 mg (58%)), which was used without further purification. IR = 3388, 1651, 1589, 1523, 1501, 1440, 1332, 1229, 988, 780, 705; ^1^H NMR (CDCl_3_, 400 MHz) *δ* = 2.93–2.96 (m, 4H, N(CH_2_)_2_), 3.38–3.41 (m, 4H, N(CH_2_)_2_), 6.05 (s, 2H, NH_2_), 7.16 (ddd, *J* = 8.2, 7.3, 1.2 Hz, 1H, 4-H), 7.35 (dd, *J* = 8.4, 1.2 Hz, 1H, 6-H), 7.60 (ddd, *J* = 8.6, 7.4, 1.6 Hz, 1H, 5-H), 7.82 (dd, *J* = 8.1, 1.6 Hz, 1H, 3-H); ^13^C NMR (CDCl_3_, 100 MHz) *δ* = 43.61 (N(CH_2_)_2_), 51.39 (N(CH_2_)_2_), 121.98 (C-6), 122.49 (C-4), 125.51 (C-3), 133.96 (C-5), 143.35 (C-2), 145.38 (C-1), 158.15 (C=O); HRMS (ESI +) calcd for C_11_H_15_N_4_O_3_ [M+H]^+^: 251.1144; found: 251.1143.

*N,N*-Dimethyl-4-(2-nitrophenyl)piperazine-1-carboxamide (**34**): To a solution of compound **29** (414 mg (2.00 mmol)) and 1,1’-carbonyldiimidazole (433 mg (2.40 mmol)) in dry dimethylformamide (4 mL), dry triethylamine (1.13 g (10.00 mmol)) was added. The reaction mixture was stirred at room temperature for 30 min. After that, dimethylamine hydrochloride (726 mg (8.00 mmol)) was added. The reaction mixture was stirred at 80 °C for 6 h. The solvent was evaporated in vacuo and the residue was mixed with water (5 mL). The aqueous phase was extracted three times with ethyl acetate. The combined organic phases were washed with 8% aq NaHCO_3_ and brine, dried over anhydrous sodium sulfate and filtered. The solvent was evaporated in vacuo giving the raw product. Purification by column chromatography (silica gel, EtAc) yielded compound **34** as yellow solid (395 mg (71%)). IR = 3441, 2843, 1645, 1605, 1520, 1488, 1451, 1385, 1349, 1287, 1232, 1210, 1173, 1044, 1002, 927, 781, 759; ^1^H NMR (CDCl_3_, 400 MHz) *δ* = 2.85 (s, 6H, N(CH_3_)_2_), 3.04–3.08 (m, 4H, N(CH_2_)_2_), 3.41–3.44 (m, 4H, N(CH_2_)_2_), 7.08 (ddd, *J* = 8.3, 7.3, 1.2 Hz, 1H, 4-H), 7.15 (dd, *J* = 8.3, 1.1 Hz, 1H, 6-H), 7.49 (ddd, *J* = 8.5, 7.4, 1.6 Hz, 1H, 5-H), 7.82 (dd, *J* = 8.2, 1.5 Hz, 1H, 3-H); ^13^C NMR (CDCl_3_, 100 MHz) *δ* = 38.47 (N(CH_3_)_2_), 46.68 (N(CH_2_)_2_), 51.63 (N(CH_2_)_2_), 121.31 (C-6), 122.31 (C-4), 125.80 (C-3), 133.49 (C-5), 143.75 (C-2), 145.88 (C-1), 164.50 (C=O); HRMS (ESI +) calcd for C_13_H_19_N_4_O_3_ [M+H]^+^: 279.1457; found: 279.1458 [M+H]^+^.

#### 3.2.4. General Procedure for the Synthesis of Compounds **31**, **42** and **43**

To an ice-cooled solution of the corresponding piperazine (2.00 mmol) in dry CH_2_Cl_2_ (8 mL), dry triethylamine (3.00 mmol) was added. After that, pivaloyl chloride (2.10 mmol) was added dropwise with a syringe through a septum. The ice-bath was removed and the reaction mixture was stirred at room temperature for 24 h. Then, the reaction was quenched with water (30 mL). The aqueous and organic phases were separated, and the organic phase was washed with 2*N* NaOH, 8% aq NaHCO_3_ and brine. It was dried over anhydrous sodium sulfate, filtered and the residue was evaporated in vacuo yielding the pivaloyl-piperazine derivative, which was either purified by column chromatography or used without further purification.

2,2-Dimethyl-1-[4-(2-nitrophenyl)piperazin-1-yl]propan-1-one (**31**): Reaction of **29** (555 mg (2.68 mmol)) with dry triethylamine (815 mg (8.05 mmol)) and pivaloyl chloride (340 mg (2.82 mmol)) in dry CH_2_Cl_2_ (11 mL) yielded compound **31** as yellow solid (687 mg (88%)), which was used without further purification. IR = 3441, 2973, 1619, 1523, 1493, 1424, 1363, 1272, 1231, 1187, 1015, 772, 751; ^1^H NMR (CDCl_3_, 400 MHz) *δ* = 1.31 (s, 9H, (CH_3_)_3_), 3.04–3.07 (m, 4H, N(CH_2_)_2_), 3.79–3.82 (m, 4H, N(CH_2_)_2_), 7.11 (td, *J* = 7.7, 1.2 Hz, 1H, 4-H), 7.15 (dd, *J* = 8.2, 1.2 Hz, 1H, 6-H), 7.51 (ddd, *J* = 8.1, 7.3, 1.6 Hz, 1H, 5-H), 7.79 (dd, *J* = 8.1, 1.6 Hz, 1H, 3-H); ^13^C NMR (CDCl_3_, 100 MHz) *δ* = 28.40 ((CH_3_)_3_), 38.67 (C*Me*_3_), 45.11 (N(CH_2_)_2_), 52.01 (N(CH_2_)_2_), 121.30 (C-6), 122.70 (C-4), 125.83 (C-3), 133.55 (C-5), 143.94 (C-2), 145.55 (C-1), 176.50 (C=O); HRMS (ESI +) calcd for C_15_H_22_N_3_O_3_ [M+H]^+^: 292.1661; found: 292.1661.

2,2-Dimethyl-1-[4-(3-nitrophenyl)piperazin-1-yl]propan-1-one (**42**): Reaction of **44** (332 mg (1.60 mmol)) with dry triethylamine (486 mg (4.80 mmol)) and pivaloyl chloride (203 mg (1.68 mmol)) in dry CH_2_Cl_2_ (8 mL) gave the raw product. Purification by column chromatography (silica gel, CH/EtAc 2:1) yielded compound **42** as yellow solid (322 mg (69%). IR = 3442, 2360, 1616, 1526, 1418, 1340, 1239, 734; ^1^H NMR (CDCl_3_, 400 MHz) *δ* = 1.33 (s, 9H, (CH_3_)_3_), 3.26–3.29 (m, 4H, N(CH_2_)_2_), 3.83–3.86 (m, 4H, N(CH_2_)_2_), 7.20 (dd, *J* = 8.3, 2.5 Hz, 1H, 6-H), 7.41 (t, *J* = 8.1 Hz, 1H, 5-H), 7.69–7.73 (m, 2 H, 2-H, 4-H); ^13^C NMR (CDCl_3_, 100 MHz) *δ* = 28.37 ((CH_3_)_3_), 38.69 (C*Me*_3_), 44.64 (N(CH_2_)_2_), 48.66 (N(CH_2_)_2_), 109.96 (C-2), 114.36 (C-4), 121.39 (C-6), 129.82 (C-5), 149.23 (C-3), 151.50 (C-1), 176.46 (C=O); HRMS (EI+) calcd for C_15_H_22_N_3_O_3_ [M+H]^+^: 292.1661; found: 292.1661.

2,2-Dimethyl-1-[4-(4-nitrophenyl)piperazin-1-yl]propan-1-one (**43**): Reaction of **45** (414 mg (2.00 mmol)) with dry triethylamine (607 mg (6.00 mmol)) and pivaloyl chloride (253 mg (2.10 mmol)) in dry CH_2_Cl_2_ (8 mL) yielded compound **43** as orange solid (513 mg (88%), which was used without further purification. IR = 3443, 1625, 1595, 1493, 1417, 1320, 1241, 1186, 1113, 1014, 753; ^1^H NMR (CDCl_3_, 400 MHz) *δ* = 1.32 (s, 9H, (CH_3_)_3_), 3.42–3.45 (m, 4H, N(CH_2_)_2_), 3.82–3.85 (m, 4H, N(CH_2_)_2_), 6.84 (d, *J* = 9.3 Hz, 2H, 2-H, 6-H), 8.15 (d, *J* = 9.3 Hz, 2H, 3-H, 5-H); ^13^C NMR (CDCl_3_, 100 MHz) *δ* = 28.22 ((CH_3_)_3_), 38.71 (C*Me*_3_), 44.42 ((NCH_2_)_2_), 47.07 ((NCH_2_)_2_), 112.88 (C-2, C- 6), 125.90 (C-3, C-5), 139.05 (C-4), 154.55 (C-1), 176.56 (C=O); HRMS (EI+) calcd for C_15_H_22_N_3_O_3_ [M+H]^+^: 292.1661; found: 292.1663.

#### 3.2.5. General Procedure for the Synthesis of Compounds **3**, **15**, **23**–**28**, **46**–**49**, **50** and **51**

To a solution of 15% (m/m) palladium on activated carbon in dry methanol (100 mL), the corresponding nitro compound (2.00 mmol) was added. The reduction of the nitro group was performed in an atmosphere of 50 psi hydrogen at the Parr-apparatus at room temperature for 24 h. After that, the reaction mixture was filtered and the solvent was evaporated in vacuo yielding the corresponding amino compound, which was either purified by column chromatography or used without further purification.

*tert*-Butyl-4-(2-aminophenyl)piperazine-1-carboxylate (**3**): Reaction of compound **5** (3.67 g (11.93 mmol)) with PdC (560 mg) in dry methanol (100 mL) gave the raw anilino derivative. It was purified by column chromatography (silica gel, CH_2_Cl_2_/*Me*OH 79:1) yielding compound **3** as pale brown solid (1.75 g (53%)). NMR data were in accordance with literature data [23].

*tert*-Butyl-*N*-(2-aminophenyl)carbamate (**15**): Reaction of compound **16** (1.12 g (4.71 mmol)) with PdC (172 mg) in dry methanol (100 mL) yielded compound **15** as orange solid (657 mg (67%)), which was used without further purification. NMR data were in accordance with literature data [40].

1-[4-(2-Aminophenyl)piperazin-1-yl]ethan-1-one (**23**): Reaction of compound **30** (1.78 g (7.14 mmol)) with PdC (268 mg) in dry methanol (80 mL) yielded compound **23** as dark-green oil (1.57 g (100%)), which was used without further purification. NMR data were in accordance with literature data [23].

4-(2-Aminophenyl)piperazine-1-carbaldehyde (**24**): Reaction of compound **32** (600 mg (2.55 mmol)) with PdC (123 mg) in dry methanol (90 mL) yielded compound **24** as pale brown solid (508 mg (97%)), which was used without further purification. IR = 3419, 3323, 2923, 2825, 1654, 1619, 1586, 1503, 1442, 1397, 1365, 1303, 1270, 1235, 1191, 1135, 1012, 918, 756; ^1^H NMR (CDCl_3_, 400 MHz) *δ* = 2.88–2.96 (m, 4H, N(CH_2_)_2_), 3.52 (t, *J* = 5.0 Hz, 2H, NCH_2_), 3.70 (br, 2H, NCH_2_), 4.00 (br s, 2H, NCH_2_), 6.73–6.77 (m, 2H, 3-H, 5-H), 6.94–7.00 (m, 2H, 4-H, 6-H), 8.10 (s, 1H, C=O); ^13^C NMR (CDCl_3_, 100 MHz) *δ* = 40.67 (NCH_2_), 46.32 (NCH_2_), 50.54 (NCH_2_), 51.70 (NCH_2_), 115.33 (C-3), 118.65 (C-5), 119.90 (C-6), 125.25 (C-4), 138.29 (C-1), 141.31 (C-2), 160.87 (C=O); HRMS (EI+) calcd for C_11_H_16_N_3_O [M+H]^+^: 206.1293; found: 206.1292.

1-[4-(2-Aminophenyl)piperazin-1-yl]-2,2-dimethylpropan-1-one (**25**): Reaction of compound **31** (555 mg (1.90 mmol)) with PdC (111 mg) in dry methanol (90 mL) yielded compound **25** as silver-grey solid (367 mg (74%)), which was used without further purification. IR = 3397, 3320, 2965, 2825, 1614, 1500, 1477, 1426, 1360, 1300, 1276, 1228, 1196, 1152, 1042, 1018, 934, 752; ^1^H NMR (CDCl_3_, 400 MHz) *δ* = 1.32 (s, 9H, (CH_3_)_3_), 2.88–2.91 (br, 4H, N(CH_2_)_2_), 3.78 (br, 4H, N(CH_2_)_2_), 3.99 (s, 2H, NH_2_), 6.72–6.76 (m, 2H, 3-H, 5-H), 6.93–6.97 (m, 2H, 4-H, 6-H); ^13^C NMR (CDCl_3_, 100 MHz) *δ* = 28.44 ((CH_3_)_3_), 38.67 (C*Me*_3_), 45.82 (N(CH_2_)_2_), 51.23 (N(CH_2_)_2_), 115.27 (C-3), 118.64 (C-5), 119.84 (C-6), 125.05 (C-4), 138.47 (C-1), 141.42 (C-2), 176.46 (C=O); HRMS (ESI +) calcd for C_15_H_24_N_3_O [M+H]^+^: 262.1919; found: 262.1919.

4-(2-Aminophenyl)-*N,N*-dimethylpiperazine-1-carboxamide (**26**): Reaction of compound **34** (407 mg (1.46 mmol)) with PdC (61 mg) in dry methanol (90 mL) yielded compound **28** as white solid (330 mg (91%)), which was used without further purification. IR = 3397, 3315, 2811, 1621, 1502, 1455, 1392, 1365, 1212, 1107, 1069, 1002, 928, 753; ^1^H NMR (CDCl_3_, 400 MHz) *δ* = 2.87 (s, 6H, N(CH_3_)_2_), 2.89–2.92 (m, 4H, N(CH_2_)_2_), 3.38 (br, 4H, N(CH_2_)_2_), 3.98 (br, 2H, NH_2_), 6.72–6.76 (m, 2H, 3-H, 5-H), 6.94 (td, *J* = 7.6, 1.2 Hz, 1H, 4-H), 6.98 (dd, *J* = 8.2, 1.3 Hz, 1H, 6-H); ^13^C NMR (CDCl_3_, 100 MHz) *δ* = 38.50 (N(CH_3_)_2_), 47.49 (N(CH_2_)_2_), 50.95 (N(CH_2_)_2_), 115.20 (C-3), 118.58 (C-5), 119.93 (C-6), 124.88 (C-4), 138.86 (C-1), 141.47 (C-2), 164.82 (C=O); HRMS (ESI +) calcd for C_13_H_21_N_4_O [M+H]^+^: 249.1715; found: 249.1714.

4-(2-Aminophenyl)piperazine-1-carboxamide (**27**): Reaction of compound **33** (404 mg (1.61 mmol)) with PdC (62 mg) in dry methanol (90 mL) yielded compound **27** as pale brown solid (333 mg (94%)), which was used without further purification. IR = 3424, 1645, 1592, 1503, 1440, 1283, 993, 754; ^1^H NMR (CDCl_3_, 400 MHz) *δ* = 2.69–2.73 (m, 4H, N(CH_2_)_2_), 3.44 (br, 4H, N(CH_2_)_2_), 4.77 (s, 2H, NH_2_), 6.00 (s, 2H, (C=O)NH_2_), 6.53 (td, *J* = 7.5, 1.5 Hz, 1H, 5-H), 6.67 (td, *J* = 7.9, 1.5 Hz, 1H, 3-H), 6.80 (td, *J* = 7.6, 1.3 Hz, 1H, 4-H),6.87 (dd, *J* = 7.8, 1.4 Hz, 1H, 6-H); ^13^C NMR (CDCl_3_, 100 MHz) *δ* = 44.12 (N(CH_2_)_2_), 50.63 (N(CH_2_)_2_), 114.55 (C-3), 116.72 (C-5), 119.32 (C-6), 124.31 (C-4), 138.13 (C-1), 142.51 (C-2), 158.33 (C=O); HRMS (EI+) calcd for C_11_H_17_N_4_ [M+H]^+^: 221.1402; found: 221.1402.

2-(4-*tert*-Butylpiperazin-1-yl)aniline (**28**): Reaction of compound **35** (619 mg (2.35 mmol)) with PdC (112 mg) in dry methanol (90 mL) yielded compound **26** as pale brown solid (472 mg (86%)), which was used without further purification. IR = 3395, 2974, 2829, 1610, 1503, 1457, 1363, 1279, 1220, 1132, 963, 760, 739; ^1^H NMR (CDCl_3_, 400 MHz) *δ* = 1.12 (s, 9H, (CH_3_)_3_), 2.73 (br, 4H, N(CH_2_)_2_), 2.95 (br, 4H, N(CH_2_)_2_), 3.97 (br, 2H, NH_2_), 6.71–6.76 (m, 2H, 3-H, 5-H), 6.92 (td, *J* = 7.6, 1.5 Hz, 1H, 4-H), 7.02 (dd, *J* = 8.3, 1.4 Hz, 1H, 6-H); ^13^C NMR (CDCl_3_, 100 MHz) *δ* = 25.92 ((CH_3_)_3_), 46.44 (N(CH_2_)_2_), 51.68 (N(CH_2_)_2_), 53.74 (C*Me*_3_), 115.00 (C-3), 118.57 (C-5), 119.91 (C-6), 124.45 (C-4), 139.34 (C-1), 141.53 (C-2); HRMS (ESI +) calcd for C_14_H_24_N_3_ [M+H]^+^: 234.1970; found: 234.1972.

*tert*-Butyl-4-(3-aminophenyl)piperazine-1-carboxylate (**46**): Reaction of compound **40** (809 mg (2.63 mmol)) with PdC (125 mg) in dry methanol (100 mL) yielded compound **46** as brown oil (657 mg (90%)), which was used without further purification. NMR data were in accordance with literature data [41].

1-[4-(3-Aminophenyl)piperazin-1-yl]-2,2-dimethylpropan-1-one (**47**): Reaction of compound **42** (291 mg (1.00 mmol)) with PdC (60 mg) in dry methanol (80 mL) gave the raw anilino derivative. The residue was dissolved in ethyl acetate and extracted with 2*N* HCl. The aqueous phases were combined and basified with 2*N* NaOH to a pH of 14. The aqueous phase was extracted with ethyl acetate. The organic phase was washed with 8% aq NaHCO_3_, dried over anhydrous sodium sulfate and filtered. The solvent was evaporated in vacuo yielding compound **47** as pale brown solid (248 mg (95%)). IR = 3471, 3338, 2972, 1614, 1503, 1426, 1364, 1283, 1210, 1193, 974, 841, 761, 689; ^1^H NMR (CDCl_3_, 400 MHz) *δ* = 1.31 (s, 9H, (CH_3_)_3_), 3.12–3.15 (m, 4H, N(CH_2_)_2_), 3.63 (br, 2H, NH_2_), 3.77–3.80 (m, 4H, N(CH_2_)_2_), 6.24–6.26 (m, 2H, 2-H, 2-H, 4-H), 6.35 (dd, *J* = 8.2, 2.0 Hz, 1H, 6-H), 7.06 (t, *J* = 8.2 Hz, 1H, 5-H); ^13^C NMR (CDCl_3_, 100 MHz) *δ* = 28.40 ((CH_3_)_3_), 38.63 (C*Me*_3_), 45.00 (N(CH_2_)_2_), 49.47 (N(CH_2_)_2_), 103.14 (C-2), 107.00 (C-6), 107.57 (C-4), 129.99 (C-5), 147.35 (C-3), 152.18 (C-1), 176.33 (C=O); HRMS (ESI +) calcd for C_15_H_24_N_3_O [M+H]^+^: 262.1919; found: 262.1920.

*tert*-Butyl-4-(4-aminophenyl)piperazine-1-carboxylate (**48**): Reaction of compound **41** (1.98 g (6.45 mmol)) with PdC (299 mg) in dry methanol (100 mL) yielded compound **48** as dark-red oil (1.66 g (93%)), which was used without further purification. NMR data were in accordance with literature data [41].

1-[4-(4-Aminophenyl)piperazin-1-yl]-2,2-dimethylpropan-1-one (**49**): Reaction of compound **43** (410 mg (1.41 mmol)) with PdC (69 mg) in dry methanol (100 mL) yielded compound **49** as dark-red oil (346 mg (94%)). IR = 3435, 2966, 1610, 1515, 1423, 1364, 1269, 1229, 1190, 1017, 831; ^1^H NMR (CDCl_3_, 400 MHz) *δ* = 1.31 (s, 9H, (CH_3_)_3_), 2.99–3.02 (m, 4H, N(CH_2_)_2_), 3.46 (br, 2H, NH_2_), 3.77–3.80 (m, 4H, N(CH_2_)_2_), 6.66 (d, *J* = 8.7 Hz, 2H, 3-H, 5-H), 6.80 (d, *J* = 8.7 Hz, 2H, 2-H, 6-H); ^13^C NMR (CDCl_3_, 100 MHz) *δ* = 28.43 ((CH_3_)_3_), 38.64 (C*Me*_3_), 45.23 ((NCH_2_)_2_), 51.34 ((NCH_2_)_2_), 116.14 (C-3, C-5), 118.96 (C-2, C-6), 140.68 (C-4), 144.04 (C-1), 176.30 (C=O); HRMS (ESI +) calcd for C_15_H_24_N_3_O [M+H]^+^: 262.1919; found: 262.1913.

*N*-(3-Aminophenyl)-2-(4-fluorophenoxy)-3-(trifluoromethyl)benzamide (**50**): Reaction of compound **52** (90 mg (0.21 mmol)) with PdC (15 mg) in dry methanol (80 mL) yielded compound **50** as pale yellow solid (45 mg (55%)). IR = 3253, 1656, 1597, 1547, 1500, 1450, 1325, 1222, 1160, 776, 686; ^1^H NMR (CDCl_3_, 400 MHz) *δ* = 6.43 (dd, *J* = 8.1, 2.2 Hz, 1H, 4″-H), 6.54 (dd, *J* = 8.0, 1.9 Hz, 1H, 6″-H), 6.74–6.78 (m, 2H, 2′-H, 6′-H), 6.90–6.95 (m, 2H, 3′-H, 5′-H), 7.02–7.06 (m, 2H, 2″-H, 5″-H), 7.53 (t, *J* = 7.8 Hz, 1H, 5-H), 7.89 (dd, *J* = 7.9, 1.7 Hz, 1H, 4-H), 8.27 (dd, *J* = 7.8, 1.7 Hz, 1H, 6-H), 8.39 (br s, 1H, NH); ^13^C NMR (CDCl_3_, 100 MHz) *δ* = 106.82 (C-2″), 110.02 (C-6″), 111.69 (C-4″), 116.20 (d, *J* = 8.3 Hz, C-2′, C-6′), 116.53 (d, *J* = 23.8 Hz, C-3′, C-5′), 122.67 (q, *J* = 273 Hz, CF_3_), 125.22 (q, *J* = 31.7 Hz, C-3), 126.28 (C-5), 129.71 (C-5″), 130.59 (q, *J* = 4.8 Hz, C-4), 130.97 (C-1), 135.89 (C-6), 138.18 (C-1″), 147.19 (C-3″), 149.43 (q, *J* = 1.8 Hz, C-2), 154.01 (d, *J* = 2.5 Hz, C-1′), 158.50 (d, *J* = 242 Hz, C-4′), 161.52 (C=O); HRMS (ESI +) calcd for C_20_H_15_F_4_N_2_O_2_ [M+H]^+^: 391.1070; found: 391.1061.

*N*-(4-Aminophenyl)-2-(4-fluorophenoxy)-3-(trifluoromethyl)benzamide (**51**): Reaction of compound **53** (58 mg (0.14 mmol)) with PdC (10 mg) in dry methanol (80 mL) gave the raw anilino derivative. It was purified by column chromatography (silica gel, CH/EtAc 1:1) yielding compound **51** as pale yellow solid (37 mg (68%)). IR = 3362, 1654, 1517, 1500, 1449, 1315, 1217, 1167, 1135, 1097, 828, 779, 685; ^1^H NMR (CDCl_3_, 400 MHz) *δ* = 3.61 (br s, 2H, NH_2_), 6.59 (d, *J* = 8.6 Hz, 2H, 3″-H, 5″-H), 6.75–6.79 (m, 2H, 2′-H, 6′-H), 6.91–6.96 (m, 2H, 3′-H, 5′-H), 7.09 (d, *J* = 8.6 Hz, 2H, 2″-H, 6″-H), 7.52 (t, *J* = 7.8 Hz, 1H, 5-H), 7.88 (dd, *J* = 7.8, 1.7 Hz, 1H, 4-H), 8.26–8.29 (m, 1H, 6-H, NH); ^13^C NMR (CDCl_3_, 100 MHz) *δ* = 115.27 (C-3″, C-5″), 116.19 (d, *J* = 8.2 Hz, C-2′, C-6′), 116.51 (d, *J* = 23.7 Hz, C-3′, C-5′), 122.35 (C-2″, C-6″), 122.71 (q, *J* = 273 Hz, CF_3_), 125.13 (q, *J* = 31.8 Hz, C-3), 126.22 (C-5), 128.31 (C-1″), 130.38 (q, *J* = 4.9 Hz, C-4), 131.02 (C-1), 135.88 (C-6), 143.91 (C-4″), 149.39 (q, *J* = 1.8 Hz, C-2), 154.03 (d, *J* = 2.5 Hz, C-1′), 158.47 (d, *J* = 242 Hz, C-4′), 161.41 (C=O); HRMS (ESI +) calcd for C_20_H_15_F_4_N_2_O_2_ [M+H]^+^: 391.1070; found: 391.1062.

#### 3.2.6. General Procedure for the Synthesis of Compounds **1**, **6**–**8**, **12**, **13**, **17**–**22**, **36**–**39** and **52**–**56**

Carboxylic acid (1.00 mmol) and anilino derivative (1.00 mmol) were dissolved in dry CH_2_Cl_2_ and cooled to 0 °C in an ice-bath. 2-Chloro-*N*-methylpyridinium iodide and diisopropylethylamine were added whereupon the ice-bath was removed. The reaction mixture was stirred at room temperature for 24–48 h. Reaction progress was monitored by TLC. Afterwards, 20% aq NH_4_Cl (50 mL) was added. The aqueous and organic phases were separated, and the aqueous phase was extracted twice with ethyl acetate. The combined organic phases were washed with 8% aq NaHCO_3_ and brine, dried over anhydrous sodium sulfate and filtered. The solvent was evaporated in vacuo giving the raw carboxamide that was purified by recrystallization or column chromatography.

*tert*-Butyl-4-{2-[2-(4-fluorophenoxy)-3-(trifluoromethyl)benzamido]phenyl}piperazine-1-carboxylate (**1**): Reaction of the carboxylic acid **2** (210 mg (0.70 mmol)) with the amine **3** (194 mg (0.70 mmol)), 2-chloro-*N*-methylpyridinium iodide (316 mg (1.24 mmol)) and DIPEA (452 mg (3.50 mmol)) in dry CH_2_Cl_2_ (30 mL) gave the raw carboxamide. Purification by column chromatography (silica gel, CH_2_Cl_2_/*Me*OH 99:1) yielded compound **1** as pale-yellow solid (51 mg (13%)). IR = 3440, 1690, 1539, 1523, 1500, 1450, 1366, 1320, 1216, 1166, 1135, 837, 777, 689; ^1^H NMR (CDCl_3_, 400 MHz) *δ* = 1.50 (s, 9H, (CH_3_)_3_), 2.81 (t, *J* = 4.8 Hz, 4H, N(CH_2_)_2_), 3.62 (br s, 4H, N(CH_2_)_2_), 6.68–6.72 (m, 2H, 2′-H, 6′-H), 6.84–6.89 (m, 2H, 3′-H, 5′-H), 7.04–7.14 (m, 3H, 3″-H, 4″-H, 5″-H), 7.53 (t, *J* = 7.6 Hz, 1H, 5-H), 7.89 (dd, *J* = 7.9, 1.6 Hz, 1H, 4-H), 8.21 (dd, *J* = 7.6, 1.6 Hz, 1H, 6-H), 8.31 (dd, *J* = 8.3, 1.6 Hz, 1H, 6″-H), 9.69 (s, 1H, NH); ^13^C NMR (CDCl_3_, 100 MHz) *δ* = 28.39 ((CH_3_)_3_), 44.12 (N(CH_2_)_2_), 52.26 (N(CH_2_)_2_), 80.13 (C*Me*_3_), 116.28 (d, *J* = 23.6 Hz, C-3′, C-5′), 116.44 (d, *J* = 7.6 Hz, C-2′, C- 6′), 119.68 (C-6″), 120.53 (C-3″), 122.67 (q, *J* = 274 Hz, CF_3_), 124.41 (C-4″), 125.31 (q, *J* = 31.8 Hz, C-3), 125.80 (C-5″), 126.18 (C-5), 130.42 (q, *J* = 4.9 Hz, C-4), 131.94 (C-1), 133.11 (C-1″), 135.28 (C-6), 141.07 (C-2″), 149.77 (q, *J* = 1.8 Hz, C-2), 154.14 (d, *J* = 2.6 Hz, C-1′), 154.65 (C=O), 158.35 (d, *J* = 242 Hz, C-4′), 161.74 ((C=O)NH); HRMS (EI+) calcd for C_29_H_29_F_4_N_3_O_4_ [M^+^]: 559.2094; found: 559.2094.

*tert*-Butyl-4-{2-[2-phenoxy-3-(trifluoromethyl)benzamido]phenyl}piperazine-1-carboxylate (**6**): Reaction of the carboxylic acid **9** (414 mg (1.47 mmol)) with the amine **3** (411 mg (1.48 mmol)), 2-chloro-*N*-methylpyridinium iodide (657 mg (2.57 mmol)) and DIPEA (949 mg (7.34 mmol)) in dry CH_2_Cl_2_ (32 mL) gave the raw carboxamide. Purification by column chromatography (silica gel, CH_2_Cl_2_/*Et*OH 79:1) yielded compound **6** as white solid (414 mg (52%)). IR = 3330, 2976, 1687, 1592, 1522, 1449, 1356, 1321, 1229, 1136, 911, 871, 752, 690; ^1^H NMR (CDCl_3_, 400 MHz) *δ* = 1.50 (s, 9H, (CH_3_)_3_), 2.81 (t, *J* = 4.9 Hz, 4H, N(CH_2_)_2_), 3.64 (br t, *J* = 5.0 Hz, 4H, N(CH_2_)_2_), 6.74 (br d, *J* = 7.9 Hz, 2H, 2′-H, 6′-H), 6.95 (br t, *J* = 7.4 Hz, 1H, 4′-H), 7.02–7.12 (m, 3H, 3″-H, 4″-H, 5″-H), 7.14–7.20 (m, 2H, 3′-H, 5′-H), 7.53 (br t, *J* = 7.8 Hz, 1H, 5-H), 7.89 (dd, *J* = 8.0, 1.7 Hz, 1H, 4-H), 8.24 (dd, *J* = 7.8, 1.7 Hz, 1H, 6-H), 8.29 (dd, *J* = 8.1, 1.8 Hz, 1H, 6″-H), 9.76 (s, 1H, NH); ^13^C NMR (CDCl_3_, 100 MHz) *δ* = 28.42 ((CH_3_)_3_), 44.07 (N(CH_2_)_2_), 52.26 (N(CH_2_)_2_), 80.07 (C*Me*_3_), 115.22 (C-2′, C-6′), 119.77 (C-6″), 120.48 (C-3″), 122.72 (q, *J* = 273 Hz, CF_3_), 123.13 (C-4′), 124.27 (C-4″), 125.41 (q, *J* = 31.7 Hz, C-3), 125.69 (C-5″), 126.02 (C-5), 129.72 (C-3′, C-5′), 130.41 (q, *J* = 4.9 Hz, C-4), 131.98 (C-1), 133.23 (C-1″), 135.29 (C-6), 141.16 (C-2″), 149.68 (q, *J* = 2.0 Hz, C-2), 154.66 (COO), 158.16 (C-1′), 161.80 (C=O); HRMS (EI+) calcd for C_29_H_30_F_3_N_3_O_4_ [M^+^]: 551.2110; found: 542.2278 [M+H]^+^.

*tert*-Butyl-4-{2-[3-(trifluoromethyl)benzamido]phenyl}piperazine-1-carboxylate (**7**): Reaction of the carboxylic acid **10** (323 mg (1.70 mmol)) with the amine **3** (287 mg (1.04 mmol)), 2-chloro-*N*-methylpyridinium iodide (463 mg (1.81 mmol)) and DIPEA (646 mg (5.00 mmol)) in dry CH_2_Cl_2_ (44 mL) gave the raw carboxamide. Purification by column chromatography (silica gel, CH_2_Cl_2_/*Me*OH 79:1) yielded compound **7** as white solid (99 mg (13%)). IR = 3324, 2854, 1687, 1591, 1521, 1456, 1395, 1368, 1247, 1166, 1125, 1072, 911, 773, 695; ^1^H NMR (CDCl_3_, 400 MHz) *δ* = 1.50 (s, 3H, (CH_3_)_3_), 2.88 (t, *J* = 5.0 Hz, 4H, N(CH_2_)_2_), 3.61 (br, 4H, N(CH_2_)_2_), 7.14 (ddd, *J* = 8.0, 7.3, 1.5 Hz, 1H, 4″-H), 7.23 (ddd, *J* = 9.6, 7.3, 1.1 Hz, 1H, 3″-H), 7.25–7.28 (m, 1H, 5″-H), 7.68 (br t, *J* = 7.7 Hz, 1H, 5-H), 7.83 (br d, *J* = 7.8 Hz, 1H, 4-H), 8.10 (br d, *J* = 7.8 Hz, 1H, 6-H), 8.20 (br s, 1H, 2-H), 8.56 (dd, *J* = 8.1, 1.4 Hz, 1H, 6″-H), 9.58 (s, 1H, NH); ^13^C NMR (CDCl_3_, 100 MHz) *δ* = 28.39 ((CH_3_)_3_), 44.53 (N(CH_2_)_2_), 52.35 (N(CH_2_)_2_), 80.22 (C*Me*_3_), 119.45 (C-6″), 123.64 (q, *J* = 273 Hz, CF_3_), 120.92 (C-3″), 123.98 (q, *J* = 3.8 Hz, C-2), 124.33 (C-4″), 126.27 (C-5″), 128.38 (q, *J* = 3.6 Hz, C-4), 129.61 (C-5), 129.95 (C-6), 131.49 (q, *J* = 32.7 Hz, C-3), 133.33 (C-1″), 135.89 (C-1), 141.09 (C-2″), 154.61 (COO), 163.10 (C=O); HRMS (ESI +) calcd for C_23_H_27_F_3_N_3_O_3_ [M+H]^+^: 450.2005; found: 450.2008.

*tert*-Butyl-4-{2-[2-(4-acetamidophenoxy)-3-(trifluoromethyl)benzamido]phenyl}piperazine-1-carboxylate (**8**): Reaction of the carboxylic acid **11** (708 mg (2.09 mmol)) with the amine **3** (579 mg (2.09 mmol)), 2-chloro-*N*-methylpyridinium iodide (933 mg (3.65 mmol)) and DIPEA (1.35 g (10.44 mmol)) in dry CH_2_Cl_2_ (100 mL) gave the raw carboxamide. Purification by column chromatography (silica gel, CH_2_Cl_2_/*Me*OH 29:1) yielded compound **8** as white solid (1.04 g (83%)). IR = 3333, 1673, 1603, 1505, 1449, 1367, 1324, 1233, 1163, 760; ^1^H NMR (DMSO-d_6_, 400 MHz) *δ* = 1.43 (s, 9H, (CH_3_)_3_), 1.96 (s, 3H, CH_3_), 2.76 (t, *J* = 4.9 Hz, 4H, N(CH_2_)_2_), 3.50 (t, *J* = 4.9 Hz, 4H, N(CH_2_)_2_), 6.70 (d, *J* = 9.0 Hz, 2H, 2′-H, 6′-H), 7.01 (td, *J* = 7.7, 1.5 Hz, 1H, 5″-H), 7.06 (td, *J* = 7.6, 1.6 Hz, 1H, 4″-H), 7.19 (br d, *J* = 7.8 Hz, 1H, 3″-H), 7.41 (d, *J* = 9.0 Hz, 2H, 3′-H, 5′-H), 7.64 (t, *J* = 7.8 Hz, 1H, 5-H), 7.68 (br d, *J* = 8.0 Hz, 1H, 6″-H), 8.00–8.06 (m, 2H, 4-H, 6-H), 9.62 (s, 1H, NH), 9.81 (s, 1H, NH); ^13^C NMR (DMSO-d_6_, 100 MHz) *δ* = 23.74 (CH_3_), 28.03 ((CH_3_)_3_), 43.65 (N(CH_2_)_2_), 51.39 (N(CH_2_)_2_), 79.00 (C*Me*_3_), 115.46 (C-2′, C-6′), 120.31 (C-3′, C-5′), 120.67 (C-3″), 120.95 (C-6″), 123.02 (q, *J* = 273 Hz, CF_3_), 123.39 (q, *J* = 30.9 Hz, C-3), 124.38 (C-5″), 124.78 (C-4″), 126.17 (C-5), 129.61 (q, *J* = 4.2 Hz, C-4), 132.08 (C-1), 132.22 (C-1″), 134.41 (C-4′), 134.64 (C-6), 142.77 (C-2″), 149.42 (q, *J* = 1.7 Hz, C-2), 153.48 (C-1′), 153.89 (COO), 162.07 (ArC=O), 167.85 (CH_3_C=O); HRMS (ESI +) calcd for C_31_H_34_F_3_N_4_O_5_ [M+H]^+^: 599.2481; found: 599.2487.

2-(4-Fluorophenoxy)-*N*-phenyl-3-(trifluoromethyl)benzamide (**12**): Reaction of the carboxylic acid **2** (303 mg (1.01 mmol)) with aniline (93 mg (1.00 mmol)), 2-chloro-*N*-methylpyridinium iodide (464 mg (1.82 mmol)) and DIPEA (646 mg (5.00 mmol)) in dry CH_2_Cl_2_ (30 mL) gave the raw carboxamide. Purification by column chromatography (silica gel, CH/EtAc 3:1) yielded compound **12** as white solid (68 mg (18%)). IR = 3317, 1657, 1579, 1529, 1502, 1444, 1339, 1313, 1249, 1217, 1166, 1132, 821, 779, 755, 687; ^1^H NMR (CDCl_3_, 400 MHz) *δ* = 6.75–6.79 (m, 2H, 2′-H, 6′-H), 6.91–6.95 (m, 2H, 3′-H, 5′-H), 7.11 (t, *J* = 7.4 Hz, 1H, 4″-H), 7.29 (t, *J* = 7.9 Hz, 2H, 3″-H, 5″-H), 7.38 (d, *J* = 7.6 Hz, 2H, 2″-H, 6″-H), 7.54 (t, *J* = 7.8 Hz, 1H, 5-H), 7.90 (dd, *J* = 7.9, 1.7 Hz, 1H, 4-H), 8.30 (dd, *J* = 7.9, 1.7 Hz, 1H, 6-H), 8.49 (br s, 1H, NH); ^13^C NMR (CDCl_3_, 100 MHz) *δ* = 116.15 (d, *J* = 8.4 Hz, C-2′, C-6′), 116.57 (d, *J* = 23.8 Hz, C-3′, C- 5′), 120.22 (C-2″, C-6″), 122.66 (q, *J* = 273 Hz, CF_3_), 125.01 (C-4″), 125.24 (q, *J* = 32.2 Hz, C-3), 126.33 (C-5), 129.04 (C-3″, C-5″), 130.70 (q, *J* = 4.6 Hz, C-4), 135.95 (C-6), 137.12 (C-1″), 149.46 (q, *J* = 1.6 Hz, C-2), 154.00 (d, *J* = 2.1 Hz, C-1′), 158.51 (d, *J* = 242 Hz, C-4′), 161.61 (C=O); HRMS (ESI +) calcd for C_20_H_13_F_4_NO_2_ [M^+^]: 375.0882; found: 375.0894.

*tert*-Butyl-*N*-{2-[2-(4-fluorophenoxy)-3-(trifluoromethyl)benzamido]phenyl}carbamate (**13**): Reaction of the carboxylic acid **2** (311 mg (1.04 mmol)) with the amine **15** (217 mg (1.04 mmol)), 2-chloro-*N*-methylpyridinium iodide (490 mg (1.92 mmol)) and DIPEA (646 mg (5.00 mmol)) in dry CH_2_Cl_2_ (30 mL) gave the raw carboxamide. Purification by column chromatography (silica gel, CH/EtAc 3:1) yielded compound **13** as dark-red solid (46 mg (9%)). IR = 3276, 1730, 1639, 1604, 1503, 1455, 1314, 1223, 1164, 842, 754; ^1^H NMR (CDCl_3_, 400 MHz) *δ* = 1.50 (s, 9H, (CH_3_)_3_), 6.50 (br s, 1H, NH), 6.80–6.84 (m, 2H, 2′-H, 6′-H), 6.92–6.97 (m, 2H, 3′-H, 5′-H), 7.10 (td, *J* = 7.4, 1.6 Hz, 1H, 5″-H), 7.15 (td, *J* = 7.6, 1.7 Hz, 1H, 4″-H), 7.24–7.27 (m, 1H, 6″-H), 7.34 (dd, *J* = 7.8, 1.6 Hz, 1H, 3″-H), 7.51 (t, *J* = 7.8 Hz, 1H, 5-H), 7.89 (dd, *J* = 7.8, 1.7 Hz, 1H, 4-H), 8.23 (dd, *J* = 7.8, 1.7 Hz, 1H, 6-H), 9.02 (br s, 1H, NH); ^13^C NMR (CDCl_3_, 100 MHz) *δ* = 28.19 ((CH_3_)_3_), 81.41 (C*Me*_3_), 116.39 (d, *J* = 23.8 Hz, C-3′, C-5′), 116.47 (d, *J* = 8.1 Hz, C-2′, C-6′), 122.66 (q, *J* = 273 Hz, CF_3_), 124.54 (C-3″), 124.97 (C-6″), 125.27 (q, *J* = 31.8 Hz, C-3), 125.77 (C-5″), 126.02 (C-5), 126.49 (C-4″), 129.83 (C-1″), 130.27 (C-2″), 130.60 (q, *J* = 4.9 Hz, C-4), 130.89 (C-1), 135.66 (C-6), 149.83 (q, *J* = 1.9 Hz, C-2), 153.95 (N(C=O)O), 154.20 (d, *J* = 2.4 Hz, C-1′), 158.40 (d, *J* = 242 Hz, C-4′), 162.67 (C=O); HRMS (ESI -) calcd for C_25_H_20_F_4_N_2_O_4_ [M-H]^−^: 489.1437; found: 489.1442

*N*-[2-(4-Acetylpiperazin-1-yl)phenyl]-2-(4-fluorophenoxy)-3-(trifluoromethyl)benzamide (**17**): Reaction of the carboxylic acid **2** (623 mg (2.08 mmol)) with the amine **23** (462 mg (2.11 mmol)), 2-chloro-*N*-methylpyridinium iodide (928 mg (3.63 mmol)) and DIPEA (1.29 g (10.00 mmol)) in dry CH_2_Cl_2_ (90 mL) gave the raw carboxamide. Purification by column chromatography (silica gel, CH_2_Cl_2_/*Me*OH 39:1) yielded compound **17** as colorless oil (31 mg (3%)). IR = 3419, 1653, 1591, 1520, 1501, 1448, 1324, 1219, 1137, 999, 834, 780; ^1^H NMR (CDCl_3_, 400 MHz) *δ* = 2.16 (s, 3H, CH_3_), 2.82–2.88 (m, 4H, 2 NCH_2_), 3.63–3.67 (m, 2H, NCH_2_), 3.82 (br, 2H, NCH_2_), 6.68–6.72 (m, 2H, 2′-H, 6′-H), 6.85–6.89 (m, 2H, 3′-H, 5′-H), 7.05–7.19 (m, 3H, 3″-H, 4″-H, 5″-H), 7.54 (t, *J* = 7.9 Hz, 1H, 5-H), 7.90 (dd, *J* = 7.9, 1.7 Hz, 1H, 4-H), 8.23 (dd, *J* = 7.9, 1.7 Hz, 1H, 6-H), 8.32 (dd, *J* = 8.0, 1.4 Hz, 1H, 6″-H), 9.70 (s, 1H, NH); ^13^C NMR (CDCl_3_, 100 MHz) *δ* = 21.36 (CH_3_), 41.94 (NCH_2_), 46.83 (NCH_2_), 52.18 (NCH_2_), 52.55 (NCH_2_), 116.37 (d, *J* = 23.7 Hz, C-3′, C-5′), 116.41 (d, *J* = 8.3 Hz, C-2′, C-6′), 119.81 (C-6″), 120.57 (C-3″), 122.66 (q, *J* = 273 Hz, CF_3_), 124.48 (C-4″), 125.33 (q, *J* = 31.8 Hz, C-3), 126.09 (C-5″), 126.28 (C-5), 130.50 (q, *J* = 4.8 Hz, C-4), 131.98 (C-1), 131.98 (C-1″), 135.35 (C-6), 140.59 (C-2″), 149.72 (q, *J* = 1.8 Hz, C-2), 154.10 (d, *J* = 2.3 Hz, C-1′), 158.40 (d, *J* = 242 Hz, C-4′), 161.73 ((C=O)NH), 169.07 (*Me*C=O); HRMS (ESI +) calcd for C_26_H_24_F_4_N_3_O_3_ [M+H]^+^: 502.1754; found: 502.1774.

2-(4-Fluorophenoxy)-*N*-[2-(4-formylpiperazin-1-yl)phenyl]-3-(trifluoromethyl)benzamide (**18**): Reaction of the carboxylic acid **2** (312 mg (1.04 mmol)) with the amine **24** (222 mg (1.08 mmol)), 2-chloro-*N*-methylpyridinium iodide (451 mg (1.77 mmol)) and DIPEA (646 mg (5.00 mmol)) in dry CH_2_Cl_2_ (40 mL) gave the raw carboxamide. Purification by column chromatography (silica gel, EtAc/CH 3:1) yielded compound **18** as white solid (314 mg (62%)). IR = 3441, 1668, 1521, 1500, 1447, 1322, 1218, 1010, 780; ^1^H NMR (CDCl_3_, 400 MHz) *δ* = 2.83–2.86 (m, 2H, NCH_2_), 2.87–2.90 (m, 2H, NCH_2_), 3.56–3.59 (m, 2H, NCH_2_), 3.76 (br, 2H, NCH_2_), 6.68–6.72 (m, 2H, 2′-H, 6′-H), 6.86–6.90 (m, 2H, 3′-H, 5′-H), 7.05–7.19 (m, 3H, 3″-H, 4″-H, 5″-H), 7.55 (t, *J* = 7.9 Hz, 1H, 5-H), 7.90 (dd, *J* = 7.9, 1.7 Hz, 1H, 4-H), 8.12 (s, 1H, HC=O), 8.24 (dd, *J* = 7.9, 1.7 Hz, 1H, 6-H), 8.33 (dd, *J* = 8.1, 1.3 Hz, 1H, 6″-H), 9.67 (s, 1H, NH); ^13^C NMR (CDCl_3_, 100 MHz) *δ* = 40.43 (NCH_2_), 46.07 (NCH_2_), 51.87 (NCH_2_), 52.98 (NCH_2_), 116.39 (d, *J* = 23.6 Hz, C-3′, C-5′), 116.41 (d, *J* = 8.1 Hz, C-2′, C-6′), 119.88 (C-6″), 120.60 (C-3″), 122.64 (q, *J* = 273 Hz, CF_3_), 124.52 (C-4″), 125.32 (q, *J* = 31.7 Hz, C-3), 126.21 (C-5″), 126.32 (C-5), 130.53 (q, *J* = 4.8 Hz, C-4), 131.99 (C-1), 133.05 (C-1″), 135.34 (C-6), 140.47 (C-2″), 149.69 (q, *J* = 1.8 Hz, C-2), 154.07 (d, *J* = 2.5 Hz, C-1′), 158.41 (d, *J* = 242 Hz, C-4′), 160.81 (H(C=O)NR_2_), 161.75 ((C=O)NH); HRMS (ESI +) calcd for C_25_H_22_F_4_N_3_O_3_ [M+H]^+^: 488.1597; found: 488.1587.

*N*-{2-[4-(2,2-Dimethylpropanoyl)piperazin-1-yl]phenyl}-2-(4-fluorophenoxy)-3-(trifluoromethyl)benzamide (**19**): Reaction of the carboxylic acid **2** (299 mg (0.99 mmol)) with the amine **25** (221 mg (0.85 mmol)), 2-chloro-*N*-methylpyridinium iodide (454 mg (1.78 mmol)) and DIPEA (646 mg (5.00 mmol)) in dry CH_2_Cl_2_ (40 mL) gave the raw carboxamide. Purification by column chromatography (silica gel, CH/EtAc 3:1) yielded compound **19** as white solid (194 mg (42%)). IR = 3441, 1631, 1520, 1501, 1449, 1325, 1219, 1139, 1016, 781; ^1^H NMR (CDCl_3_, 400 MHz) *δ* = 1.33 (s, 9H, (CH_3_)_3_), 2.83–2.86 (m, 4H, N(CH_2_)_2_), 3.85 (br, 4H, N(CH_2_)_2_), 6.68–6.74 (m, 2H, 2′-H, 6′-H), 6.84–6.90 (m, 2H, 3′-H, 5′-H), 7.07 (td, *J* = 7.5, 1.6 Hz, 1H, 4″-H), 7.11–7.16 (m, 2H, 3″-H, 5″-H), 7.54 (td, *J* = 7.9, 0.9 Hz, 1H, 5-H), 7.90 (dd, *J* = 7.9, 1.7 Hz, 1H, 4-H), 8.24 (dd, *J* = 7.9, 1.7 Hz, 1H, 6-H), 8.32 (dd, *J* = 8.3, 1.6 Hz, 1H, 6″-H), 9.74 (s, 1H, NH); ^13^C NMR (CDCl_3_, 100 MHz) *δ* = 28.40 ((CH_3_)_3_), 38.71 (C*Me*_3_), 45.61 (N(CH_2_)_2_), 52.50 (N(CH_2_)_2_), 116.35 (d, *J* = 23.7 Hz, C-3′, C-5′), 116.45 (d, *J* = 8.3 Hz, C-2′, C-6′), 119.77 (C-6″), 120.51 (C-3″), 122.67 (q, *J* = 273 Hz, CF_3_), 124.45 (C-4″), 125.34 (q, *J* = 31.7 Hz, C-3), 125.95 (C-5″), 126.25 (C-5), 130.47 (q, *J* = 4.9 Hz, C-4), 131.91 (C-1), 133.14 (C-1″), 135.37 (C-6), 140.63 (C-2″), 149.78 (q, *J* = 1.9 Hz, C-2), 154.13 (d, *J* = 2.5 Hz, C-1′), 158.38 (d, *J* = 242 Hz, C-4′), 161.67 ((C=O)NH), 176.52 (C=O); HRMS (ESI +) calcd for C_29_H_30_F_4_N_3_O_3_ [M+H]^+^: 544.2223; found: 544.2214.

2-(4-Fluorophenoxy)-*N*-[2-(4-tert-butylpiperazin-1-yl)phenyl]-3-(trifluoromethyl)benzamide (**20**): Reaction of the carboxylic acid **2** (304 mg (1.01 mmol)) with the amine **26** (220 mg (0.94 mmol)), 2-chloro-*N*-methylpyridinium iodide (453 mg (1.77 mmol)) and DIPEA (646 mg (5.00 mmol)) in dry CH_2_Cl_2_ (40 mL) gave the raw carboxamide. Purification by column chromatography (silica gel, EtAc) was followed by recrystallization from CH yielding compound **20** as white solid (99 mg (20%)). m.P. 142–145 °C; IR = 3447, 2974, 1672, 1588, 1501, 1447, 1323, 1214, 1165, 1129, 782; ^1^H NMR (CDCl_3_, 400 MHz) *δ* = 1.14 (s, 9H, (CH_3_)_3_), 2.82 (br, 4H, N(CH_2_)_2_), 2.90–2.93 (m, 4H, N(CH_2_)_2_), 6.69–6.73 (m, 2H, 2′-H, 6′-H), 6.83–6.87 (m, 2H, 3′-H, 5′-H), 7.03–7.12 (m, 2H, 4″-H, 5″-H), 7.18 (dd, *J* = 7.6, 1.8 Hz, 3″-H), 7.53 (t, *J* = 7.8 Hz, 1H, 5-H), 7.89 (dd, *J* = 7.8, 1.7 Hz, 1H, 4-H), 8.26 (dd, *J* = 7.9, 1.7 Hz, 1H, 6-H), 8.33 (dd, *J* = 7.8, 1.7 Hz, 1H, 6″-H), 9.96 (s, 1H, NH); ^13^C NMR (CDCl_3_, 100 MHz) *δ* = 25.80 ((CH_3_)_3_), 46.32 (N(CH_2_)_2_), 53.03 (N(CH_2_)_2_), 53.84 (C*Me*_3_), 116.25 (d, *J* = 23.7 Hz, C-3′, C-5′), 116.33 (d, *J* = 8.0 Hz, C-2′, C-6′), 119.24 (C-6″), 120.56 (C-3″), 122.76 (q, *J* = 273 Hz, CF_3_), 124.25 (C-4″), 125.34 (q, *J* = 31.7 Hz, C-3), 125.50 (C-5″), 126.11 (C-5), 130.28 (q, *J* = 4.9 Hz, C-4), 132.00 (C-1), 133.36 (C-1″), 135.57 (C-6), 141.39 (C-2″), 149.78 (q, *J* = 1.9 Hz, C-2), 154.30 (d, *J* = 2.5 Hz, C-1′), 158.30 (d, *J* = 242 Hz, C-4′), 161.48 ((C=O)NH); HRMS (EI+) calcd for C_28_H_30_F_4_N_3_O_2_ [M+H]^+^: 516.2274; found: 516.2266.

*N*-[2-(4-Carbamoylpiperazin-1-yl)phenyl]-2-(4-fluorophenoxy)-3-(trifluoromethyl)benzamide (**21**): Reaction of the carboxylic acid **2** (297 mg (0.99 mmol)) with the amine **27** (220 mg (1.00 mmol)), 2-chloro-*N*-methylpyridinium iodide (453 mg (1.77 mmol)) and DIPEA (646 mg (5.00 mmol)) in dry CH_2_Cl_2_ (40 mL) gave the raw carboxamide. Purification by column chromatography (silica gel, EtAc) yielded compound **21** as white solid (239 mg (48%)). IR = 3355, 1656, 1592, 1500, 1448, 1325, 1219, 1139, 988, 831, 780; ^1^H NMR (CDCl_3_, 400 MHz) *δ* = 2.85–2.88 (m, 4H, N(CH_2_)_2_), 3.59–3.61 (m, 4H, N(CH_2_)_2_), 4.60 (s, 2H, NH_2_), 6.68–6.72 (m, 2H, 2′-H, 6′-H), 6.84–6.89 (m, 2H, 3′-H, 5′-H), 7.05–7.16 (m, 3H, 3″-H, 4″-H, 5″-H), 7.54 (t, *J* = 7.8 Hz, 1H, 5-H), 7.89 (dd, *J* = 7.9, 1.6 Hz, 1H, 4-H), 8.22 (dd, *J* = 7.8, 1.6 Hz, 1H, 6-H), 8.30 (dd, *J* = 7.9, 2.0 Hz, 1H, 6″-H), 9.67 (s, 1H, NH); ^13^C NMR (CDCl_3_, 100 MHz) *δ* = 44.63 (N(CH_2_)_2_), 52.09 (N(CH_2_)_2_), 116.34 (d, *J* = 23.8 Hz, C-3′, C-5′), 116.45 (d, *J* = 8.3 Hz, C-2′, C-6′), 119.80 (C-6″), 120.55 (C-3″), 122.67 (q, *J* = 273 Hz, CF_3_), 124.49 (C-4″), 125.34 (q, *J* = 31.7 Hz, C-3), 126.01 (C-5″), 126.24 (C-5), 130.46 (q, *J* = 4.9 Hz, C-4), 131.99 (C-1), 133.12 (C-1″), 135.29 (C-6), 140.74 (C-2″), 149.75 (q, *J* = 2.2 Hz, C-2), 154.12 (d, *J* = 2.5 Hz, C-1′), 157.80 ((C=O)NH2), 158.39 (d, *J* = 242 Hz, C-4′), 161.75 ((C=O)NH); HRMS (ESI +) calcd for C_25_H_23_F_4_N_4_O_3_ [M+H]^+^: 503.1706; found: 503.1700.

*N*-{2-[4-(*N,N*-Dimethylcarbamoyl)piperazin-1-yl]phenyl}-2-(4-fluorophenoxy)-3-(trifluoromethyl)benzamide (**22**): Reaction of the carboxylic acid **2** (339 mg (1.13 mmol)) with the amine **28** (279 mg (1.12 mmol)), 2-chloro-*N*-methylpyridinium iodide (509 mg (1.99 mmol)) and DIPEA (724 mg (5.60 mmol)) in dry CH_2_Cl_2_ (50 mL) gave the raw carboxamide. Purification by column chromatography (silica gel, EtAc/CH 2:1) yielded compound **22** as yellow oil (244 mg (41%)). IR = 3333, 2848, 1649, 1591, 1500, 1448, 1394, 1328, 1218, 1138, 828, 781; ^1^H NMR (CDCl_3_, 400 MHz) *δ* = 2.84–2.87 (m, 4H, N(CH_2_)_2_), 2.87 (s, 6H, N(CH_3_)_2_), 3.42–3.46 (m, 4H, N(CH_2_)_2_), 4.60 (s, 2H, NH_2_), 6.69–6.73 (m, 2H, 2′-H, 6′-H), 6.83–6.88 (m, 2H, 3′-H, 5′-H), 7.04–7.18 (m, 3H, 3″-H, 4″-H, 5″-H), 7.53 (t, *J* = 7.8 Hz, 1H, 5-H), 7.89 (dd, *J* = 7.9, 1.7 Hz, 1H, 4-H), 8.20 (dd, *J* = 7.8, 1.7 Hz, 1H, 6-H), 8.28 (dd, *J* = 7.9, 1.6 Hz, 1H, 6″-H), 9.66 (s, 1H, NH); ^13^C NMR (CDCl_3_, 100 MHz) *δ* = 38.52 (N(CH_3_)_2_), 47.30 (N(CH_2_)_2_), 52.23 (N(CH_2_)_2_), 116.28 (d, *J* = 23.7 Hz, C-3′, C-5′), 116.53 (d, *J* = 8.2 Hz, C-2′, C-6′), 119.70 (C-6″), 120.65 (C-3″), 122.70 (q, *J* = 273 Hz, CF_3_), 124.44 (C-4″), 125.38 (q, *J* = 31.7 Hz, C-3), 125.80 (C-5″), 126.16 (C-5), 130.39 (q, *J* = 4.8 Hz, C-4), 132.01 (C-1), 133.16 (C-1″), 135.18 (C-6), 141.07 (C-2″), 149.87 (q, *J* = 1.9 Hz, C-2), 154.20 (d, *J* = 2.5 Hz, C-1′), 158.36 (d, *J* = 242 Hz, C-4′), 161.81 ((C=O)NH), 164.52 ((C=O)NR_2_); HRMS (ESI +) calcd for C_27_H_27_F_4_N_4_O_3_ [M+H]^+^: 531.2019; found: 531.2026

*tert*-Butyl-4-{3-[2-(4-fluorophenoxy)-3-(trifluoromethyl)benzamido]phenyl}piperazine-1-carboxylate (**36**): Reaction of the carboxylic acid **2** (329 mg (1.10 mmol)) with the amine **46** (302 mg (1.09 mmol)), 2-chloro-*N*-methylpyridinium iodide (486 mg (1.90 mmol)) and DIPEA (698 mg (5.40 mmol)) in dry CH_2_Cl_2_ (35 mL) gave the raw carboxamide. Purification by column chromatography (silica gel, CH/EtAc 2:1) yielded compound **36** as pale-yellow solid (177 mg (29%)). IR = 3422, 1691, 1609, 1501, 1450, 1332, 1248, 1221, 1166, 998, 777, 688; ^1^H NMR (CDCl_3_, 400 MHz) *δ* = 1.48 (s, 9H, (CH_3_)_3_), 3.09–3.12 (m, 4H, N(CH_2_)_2_), 3.54–3.57 (m, 4H, N(CH_2_)_2_), 6.65–6.70 (m, 2H, 4″-H, 6″-H), 6.75–6.79 (m, 2H, 2′-H, 6′-H), 6.91–6.96 (m, 2H, 3′-H, 5′-H), 7.16 (t, *J* = 8.1 Hz, 1H, 5″-H), 7.18 (d, *J* = 2.5 Hz, 1H, 2″-H), 7.54 (t, *J* = 7.8 Hz, 1H, 5-H), 7.90 (dd, *J* = 7.9, 1.7 Hz, 1H, 4-H), 8.29 (dd, *J* = 7.9, 1.7 Hz, 1H, 6-H), 8.44 (br s, 1H, NH); ^13^C NMR (CDCl_3_, 100 MHz) *δ* = 28.42 ((CH_3_)_3_), 43.46 ((NCH_2_)_2_), 49.04 ((NCH_2_)_2_), 79.92 (C*Me*_3_), 108.47 (C-2″), 111.75 (C-6″), 112.95 (C-4″), 116.17 (d, *J* = 7.3 Hz, C-2′, C-6′), 116.57 (d, *J* = 23.7 Hz, C-3′, C-5′), 122.66 (q, *J* = 274 Hz, CF_3_), 125.25 (q, *J* = 31.8 Hz, C-3), 126.35 (C-5), 129.54 (C-5″), 130.69 (q, *J* = 5.0 Hz, C-4), 130.87 (C-1), 135.90 (C-6), 138.09 (C-1″), 149.40 (q, *J* = 2.0 Hz, C-2), 151.86 (C-3″), 154.01 (d, *J* = 2.2 Hz, C-1′), 154.69 (COO), 158.50 (d, *J* = 242 Hz, C-4′), 161.59 (C=O); HRMS (ESI +) calcd for C_29_H_30_F_4_N_3_O_4_ [M+H]^+^: 560.2172; found: 560.2163.

*tert*-Butyl-4-{4-[2-(4-fluorophenoxy)-3-(trifluoromethyl)benzamido]phenyl}piperazine-1-carboxylate (**37**): Reaction of the carboxylic acid **2** (305 mg (1.02 mmol)) with the amine **48** (280 mg (1.01 mmol)), 2-chloro-*N*-methylpyridinium iodide (452 mg (1.77 mmol)) and DIPEA (646 mg (5.00 mmol)) in dry CH_2_Cl_2_ (30 mL) gave the raw carboxamide. Purification by column chromatography (silica gel, CH/EtAc 3:1) yielded compound **37** as pale-yellow solid (23 mg (4%)). IR = 3422, 1662, 1502, 1450, 1315, 1219, 1163, 824, 782; ^1^H NMR (CDCl_3_, 400 MHz) *δ* = 1.48 (s, 9H, (CH_3_)_3_), 3.06–3.19 (m, 4H, N(CH_2_)_2_), 3.54–3.57 (m, 4H, N(CH_2_)_2_), 6.75–6.79 (m, 2H, 2′-H, 6′-H), 6.84 (d, *J* = 8.8 Hz, 2H, 3″-H, 5″-H), 6.91–6.96 (m, 2H, 3′-H, 5′-H), 7,24 (d, J = 8.8 Hz, 2H, 2″-H, 6″-H), 7.53 (t, *J* = 7.8 Hz, 1H, 5-H), 7.88 (dd, *J* = 7.4, 1.5 Hz, 1H, 4-H), 8.28 (dd, *J* = 7.9, 1.8 Hz, 1H, 6-H), 8.37 (br s, 1H, NH); ^13^C NMR (CDCl_3_, 100 MHz) *δ* = 28.41 ((CH_3_)_3_), 43.51 ((NCH_2_)_2_), 49.55 ((NCH_2_)_2_), 79.92 (C*Me*_3_), 116.16 (d, *J* = 8.3 Hz, C-2′, C-6′), 116.53 (d, *J* = 23.7 Hz, C-3′, C-5′), 116.98 (C-3″, C-5″), 121.73 (C-2″, C-6″), 122.68 (q, *J* = 273 Hz, CF_3_), 125.17 (q, *J* = 31.8 Hz, C-3), 126.27 (C-5), 129.84 (C-1″), 130.50 (q, *J* = 4.9 Hz, C-4), 130.90 (C-1), 135.89 (C-6), 148.70 (C-4″), 149.39 (q, *J* = 2.8 Hz, C-2), 154.01 (d, *J* = 2.3 Hz, C-1′), 154.67 (COO), 158.48 (d, *J* = 242 Hz, C-4′), 161.42 (C=O); HRMS (ESI +) calcd for C_29_H_30_F_4_N_3_O_4_ [M+H]^+^: 560.2172; found: 560.2162.

*N*-{3-[4-(2,2-Dimethylpropanoyl)piperazin-1-yl]phenyl}-2-(4-fluorophenoxy)-3-(trifluoromethyl)benzamide (**38**): Reaction of the carboxylic acid **2** (221 mg (0.74 mmol)) with the amine **47** (183 mg (0.70 mmol)), 2-chloro-*N*-methylpyridinium iodide (337 mg (1.49 mmol)) and DIPEA (452 mg (3.50 mmol)) in dry CH_2_Cl_2_ (30 mL) gave the raw carboxamide. Purification by column chromatography (silica gel, CHCl_3_/EtAc 2:1) yielded compound **38** as white solid (91 mg (24%)). IR = 2978, 1608, 1543, 1501, 1449, 1332, 1220, 1188, 997, 837, 777, 688; ^1^H NMR (CDCl_3_, 400 MHz) *δ* = 1.31 (s, 9H, (CH_3_)_3_), 3.12–3.15 (m, 4H, N(CH_2_)_2_), 3.76–3.79 (m, 4H, N(CH_2_)_2_), 6.65–6.68 (m, 2H, 4″-H, 6″-H), 6.75–6.79 (m, 2H, 2′-H, 6′-H), 6.91–6.95 (m, 2H, 3′-H, 5′-H), 7.17 (t, *J* = 7.6 Hz, 1H, 5″-H), 7.21 (t, *J* = 2.1 Hz, 1H, 2″-H), 7.54 (t, *J* = 7.8 Hz, 1H, 5-H), 7.90 (dd, *J* = 7.9, 1.6 Hz, 1H, 4-H), 8.28 (dd, *J* = 7.8, 1.6 Hz, 1H, 6-H), 8.45 (br s, 1H, NH); ^13^C NMR (CDCl_3_, 100 MHz) *δ* = 28.40 ((CH_3_)_3_), 38.65 (C*Me*_3_), 44.88 (N(CH_2_)_2_), 49.17 (N(CH_2_)_2_), 108.26 (C-2″), 111.81 (C-6″), 112.75 (C-4″), 116.18 (d, *J* = 8.2 Hz, C-2′, C-6′), 116.56 (d, J = 23.7 Hz, C-3′, C-5′), 122.66 (q, *J* = 274 Hz, CF_3_), 125.26 (q, *J* = 31.6 Hz, C-3), 126.35 (C-5), 129.55 (C-5″), 130.69 (q, *J* = 4.9 Hz, C-4), 130.85 (C-1), 135.85 (C-6), 138.122 (C-1″), 149.41 (q, *J* = 1.8 Hz, C-2), 151.60 (C-3″), 154.02 (d, *J* = 2.5 Hz, C-1′), 158.49 (d, *J* = 242 Hz, C-4′), 161.65 ((C=O)NH), 176.38 (C=O); HRMS (ESI +) calcd for C_29_H_30_F_4_N_3_O_3_ [M+H]^+^: 544.2223; found: 544.2217.

*N*-{4-[4-(2,2-Dimethylpropanoyl)piperazin-1-yl]phenyl}-2-(4-fluorophenoxy)-3-(trifluoromethyl)benzamide (**39**): Reaction of the carboxylic acid **2** (307 mg (1.03 mmol)) with the amine **49** (256 mg (0.98 mmol)), 2-chloro-*N*-methylpyridinium iodide (440 mg (1.72 mmol)) and DIPEA (633 mg (4.90 mmol)) in dry CH_2_Cl_2_ (30 mL) gave the raw carboxamide. Purification by column chromatography (silica gel, CH_2_Cl_2_/*Et*OH 59:1) yielded compound **39** as white solid (96 mg (18%)). IR = 3275, 1660, 1609, 1514, 1503, 1449, 1316, 1223, 1186, 1156, 1098, 1016, 824, 781; ^1^H NMR (CDCl_3_, 400 MHz) *δ* = 1.31 (s, 9H, (CH_3_)_3_), 3.09–3.12 (m, 4H, N(CH_2_)_2_), 3.77–3.80 (m, 4H, N(CH_2_)_2_), 6.75–6.78 (m, 2H, 2′-H, 6′-H), 6.84 (d, *J* = 8.9 Hz, 2H, 3″-H, 5″-H), 6.91–6.96 (m, 2H, 3′-H, 5′-H), 7.25 (d, *J* = 8.9 Hz, 2H, 2″-H, 6″-H), 7.53 (t, *J* = 7.8 Hz, 1H, 5-H), 7.89 (dd, *J* = 7.8, 1.6 Hz, 1H, 4-H), 8.29 (dd, *J* = 7.9, 1.7 Hz, 1H, 6-H), 8.38 (br s, 1H, NH); ^13^C NMR (CDCl_3_, 100 MHz) *δ* = 28.41 ((CH_3_)_3_), 38.66 (C*Me*_3_), 44.93 ((NCH_2_)_2_), 49.70 ((NCH_2_)_2_), 116.16 (d, *J* = 8.2 Hz, C-2′, C-6′), 116.53 (d, *J* = 23.8 Hz, C-3′, C-5′), 116.78 (C-3″, C-5″), 121.72 (C-2″, C-6″), 122.68 (q, *J* = 273 Hz, CF_3_), 125.18 (q, *J* = 31.8 Hz, C-3), 126.76 (C-5), 129.97 (C-1″), 130.51 (q, *J* = 4.9 Hz, C-4), 130.88 (C-1), 135.89 (C-6), 148.40 (C-4″), 149.40 (q, *J* = 1.9 Hz, C-2), 154.01 (d, *J* = 2.5 Hz, C-1′), 158.48 (d, *J* = 242 Hz, C-4′), 161.43 (C=O), 176.37 ((C=O)NR_2_); HRMS (ESI +) calcd for C_29_H_30_F_4_N_3_O_3_ [M+H]^+^: 544.2223; found: 544.2214.

2-(4-Fluorophenoxy)-*N*-(3-nitrophenyl)-3-(trifluoromethyl)benzamide (**52**): Reaction of the carboxylic acid **2** (309 mg (1.03 mmol)) with 3-nitroaniline (145 mg (1.05 mmol)), 2-chloro-*N*-methylpyridinium iodide (454 mg (1.78 mmol)) and DIPEA (646 mg (5.00 mmol)) in dry CH_2_Cl_2_ (30 mL) gave the raw carboxamide. Purification by column chromatography (silica gel, CH_2_Cl_2_) yielded compound **52** as pale-yellow solid (156 mg (36%)). IR = 3368, 1695, 1601, 1548, 1503, 1450, 1351, 1287, 1266, 1222, 1182, 1167, 1127, 1098, 825, 782, 738; ^1^H NMR (CDCl_3_, 400 MHz) *δ* = 6.77–6.80 (m, 2H, 2′-H, 6′-H), 6.93–6.98 (m, 2H, 3′-H, 5′-H), 7.47 (t, *J* = 8.2 Hz, 1H, 5″-H), 7.58 (t, *J* = 7.8 Hz, 1H, 5-H), 7.76 (dd, *J* = 8.1, 2.1 Hz, 1H, 6″-H), 7.94–7.98 (m, 2H, 4-H, 4″-H), 8.31–8.34 (m, 2H, 2″-H, 6-H), 8.76 (br s, 1H, NH); ^13^C NMR (CDCl_3_, 100 MHz) *δ* = 114.89 (C-2″), 115.99 (d, *J* = 8.3 Hz, C-2′, C-6′), 116.80 (d, *J* = 23.8 Hz, C-3′, C-5′), 119.57 (C-4″), 122.53 (q, *J* = 273 Hz, CF_3_), 125.47 (q, *J* = 32.0 Hz, C-3), 125.62 (C-6″), 126.60 (C-5), 129.88 (C-1), 129.91 (C-5″), 131.35 (q, *J* = 5.0 Hz, C-4), 136.01 (C-6), 138.23 (C-1″), 148.56 (C-3″), 149.49 (q, *J* = 1.9 Hz, C-2), 153.89 (d, *J* = 2.7 Hz, C-1′), 158.62 (d, *J* = 243 Hz, C-4′), 161.94 (C=O); HRMS (EI+) calcd for C_20_H_11_F_4_N_2_O_4_ [M-H]^−^: 419.0655; found: 419.0660.

2-(4-Fluorophenoxy)-*N*-(4-nitrophenyl)-3-(trifluoromethyl)benzamide (**53**): Reaction of the carboxylic acid **2** (301 mg (1.00 mmol)) with 4-nitroaniline (141 mg (1.03 mmol)), 2-chloro-*N*-methylpyridinium iodide (467 mg (1.83 mmol)) and DIPEA (646 mg (5.00 mmol)) in dry CH_2_Cl_2_ (30 mL) gave the raw carboxamide. Purification by column chromatography (silica gel, CH_2_Cl_2/_AcOH 100:1) yielded compound **53** as white solid (84 mg (20%)). IR = 3299, 1660, 1597, 1502, 1449, 1406, 1345, 1304, 1256, 1219, 1167, 1134, 834, 778, 751, 696; ^1^H NMR (CDCl_3_, 400 MHz) *δ* = 6.74–6.78 (m, 2H, 2′-H, 6′-H), 6.92–6.96 (m, 2H, 3′-H, 5′-H), 7.59 (t, *J* = 7.8 Hz, 1H, 5-H), 7.62 (d, *J* = 9.1 Hz, 2H, 2″-H, 6″-H), 7.96 (dd, *J* = 7.7, 1.6 Hz, 1H, 4-H), 8.19 (d, *J* = 9.1 Hz, 2H, 3″-H, 5″-H), 8.32 (dd, *J* = 7.7, 1.6 Hz, 1H, 6-H), 8.85 (br s, 1H, NH); ^13^C NMR (CDCl_3_, 100 MHz) *δ* = 115.98 (d, *J* = 8.3 Hz, C-2′, C-6′), 116.80 (d, *J* = 24.0 Hz, C-3′, C-5′), 119.39 (C-2″, C-6″), 122.50 (q, *J* = 274 Hz, CF_3_), 125.10 (C-3″, C-5″), 125.51 (q, *J* = 31.8 Hz, C-3), 126.64 (C-5), 129.83 (C-1), 131.49 (q, *J* = 4.8 Hz, C-4), 136.04 (C-6), 142.88 (C-1″), 144.02 (C-4″), 149.53 (q, *J* = 1.9 Hz, C-2), 153.85 (d, *J* = 2.5 Hz, C-1′), 158.62 (d, *J* = 243 Hz, C-4′), 161.91 (C=O); HRMS (ESI -) calcd for C_20_H_11_F_4_N_2_O_4_ [M-H]^−^: 419.0655; found: 419.0660.

*tert*-Butyl-4-{4-[2-phenoxy-3-(trifluoromethyl)benzamido]phenyl}piperazine-1-carboxylate (**54**): Reaction of the carboxylic acid **9** (264 mg (0.94 mmol)) with the amine **48** (274 mg (0.99 mmol)), 2-chloro-*N*-methylpyridinium iodide (438 mg (1.71 mmol)) and DIPEA (607 mg (4.70 mmol)) in dry CH_2_Cl_2_ (30 mL) gave the raw carboxamide. Purification by column chromatography (silica gel, CH/EtAc 2:1) yielded compound **54** as pale-yellow solid (153 mg (30%)). IR = 3309, 1689, 1518, 1449, 1316, 1234, 1164, 750; ^1^H NMR (CDCl_3_, 400 MHz) *δ* = 1.48 (s, 9H, (CH_3_)_3_), 3.04–3.08 (m, 4H, N(CH_2_)_2_), 3.53–3.57 (m, 4H, N(CH_2_)_2_), 6.80–6.83 (m, 4H, 2′-H, 3″-H, 5″-H, 6′-H), 7.03 (t, *J* = 7.4 Hz, 1H, 4′-H), 7.21 (d, *J* = 8.8 Hz, 2H, 2″-H, 6″-H), 7.25 (t, *J* = 8.0 Hz, 2H, 3′-H, 5′-H), 7.53 (t, *J* = 7.8 Hz, 1H, 5-H), 7.89 (dd, *J* = 7.9, 1.7 Hz, 1H, 4-H), 8.33 (dd, *J* = 7.9, 1.7 Hz, 1H, 6-H), 8.48 (br s, 1H, NH); ^13^C NMR (CDCl_3_, 100 MHz) *δ* = 28.41 ((CH_3_)_3_), 43.50 (N(CH_2_)_2_), 49.58 (N(CH_2_)_2_), 79.90 (C*Me*_3_), 114.92 (C-2′, C-6′), 116.95 (C-3″, C-5″), 121.87 (C-2″, C-6″), 122.74 (q, *J* = 274 Hz, CF_3_), 123.32 (C-4′), 125.28 (q, J = 31.6 Hz, C-3), 125.13 (C-5), 126.33 (C-5), 129.97 (C-1″), 130.00 (C-3′, C- 5′), 130.52 (q, *J* = 5.0 Hz, C-4), 130.80 (C-1), 135.94 (C-6), 148.62 (C-4″), 154.67 (N(C=O)O), 158.03 (C-1″), 161.44 (C=O); HRMS (ESI +) calcd for C_29_H_31_F_3_N_3_O_4_ [M+H]^+^: 542.2267; found: 542.2255.

*N*-{4-[4-(2,2-Dimethylpropanoyl)piperazin-1-yl]phenyl}-2-phenoxy-3-(trifluoromethyl)benzamide (**55**): Reaction of the carboxylic acid **9** (260 mg (0.92 mmol)) with the amine **49** (252 mg (0.96 mmol)), 2-chloro-*N*-methylpyridinium iodide (445 mg (1.74 mmol)) and DIPEA (595 mg (4.60 mmol)) in dry CH_2_Cl_2_ (30 mL) gave the raw carboxamide. Purification by column chromatography (silica gel, CH/EtAc 1:1) yielded compound **55** as pale-yellow solid (97 mg (20%)). IR = 3423, 1625, 1516, 1448, 1316, 1234, 1162, 751, 688; ^1^H NMR (CDCl_3_, 400 MHz) *δ* = 1.30 (s, 9H, (CH_3_)_3_), 3.08–3.11 (m, 4H, N(CH_2_)_2_), 3.76–3.79 (m, 4H, N(CH_2_)_2_), 6.80–6.83 (m, 4H, 2′-H, 3″-H, 5″-H, 6′-H), 7.03 (t, *J* = 7.4 Hz, 1H, 4′-H), 7.20–7.28 (m, 4H, 2″-H, 3′-H, 5′-H, 6″-H) 7.53 (t, *J* = 7.8 Hz, 1H, 5-H), 7.89 (dd, *J* = 7.9, 1.7 Hz, 1H, 4-H), 8.33 (dd, *J* = 7.9, 1.7 Hz, 1H, 6-H), 8.49 (s, 1H, NH); ^13^C NMR (CDCl_3_, 100 MHz) *δ* = 28.41 ((CH_3_)_3_), 38.66 (C*Me*_3_), 44.94 (N(CH_2_)_2_), 49.74 (N(CH_2_)_2_), 114.92 (C-2′, C-6′), 116.75 (C-3″, C-5″), 121.87 (C-2″, C-6″), 122.73 (q, *J* = 274 Hz, CF_3_), 123.32 (C-4′), 125.29 (q, J = 31.7 Hz, C-3), 126.14 (C-5), 130.00 (C-3′, C-5′), 130.09 (C-1″), 130.53 (q, *J* = 4.9 Hz, C-4), 130.77 (C-1), 135.94 (C-6), 148.32 (C-4″), 149.30 (q, *J* = 1.8 Hz, C-2), 158.03 (C-1″), 161.46 (N(C=O)O), 176.36 (C=O); HRMS (ESI +) calcd for C_29_H_31_F_3_N_3_O_3_ [M+H]^+^: 526.2318; found: 526.2310.

*N*-{2-[4-(2,2-Dimethylpropanoyl)piperazin-1-yl]phenyl}-2-phenoxy-3-(trifluoromethyl)benzamide (**56**): Reaction of the carboxylic acid **9** (163 mg (0.58 mmol)) with the amine **25** (148 mg (0.57 mmol)), 2-chloro-*N*-methylpyridinium iodide (268 mg (1.05 mmol)) and DIPEA (368 mg (2.85 mmol)) in dry CH_2_Cl_2_ (30 mL) gave the raw carboxamide. Purification by column chromatography (silica gel, CH/EtAc 3:1) yielded compound **56** as white solid (141 mg (47%)). IR = 3442, 1685, 1636, 1588, 1521, 1448, 1326, 1160, 1122, 799, 752, 690; ^1^H NMR (CDCl_3_, 400 MHz) *δ* = 1.33 (s, 9H, (CH_3_)_3_), 2.84–2.87 (m, 4H, N(CH_2_)_2_), 3.87 (br s, 4H, N(CH_2_)_2_), 6.75 (d, *J* = 8.2 Hz, 2H, 2′-H, 6′-H), 6.97 (t, *J* = 7.4 Hz, 1H, 4′-H), 7.05 (t, *J* = 7.9 Hz, 1H; 4″-H), 7.09–7.14 (m, 2H, 3″-H, 5″-H), 7.19 (t, *J* = 7.9 Hz, 2H, 3′-H, 5′-H), 7.54 (t, *J* = 7.8 Hz, 1H, 5-H), 7.90 (dd, *J* = 7.8, 1.7 Hz, 1H, 4-H), 8.27 (br d, *J* = 7.8 Hz, 1H, 6-H), 8.31 (br d, *J* = 7.9 Hz, 1H, 6″-H), 9.82 (s, 1H, NH); ^13^C NMR (CDCl_3_, 100 MHz) *δ* = 28.42 ((CH_3_)_3_), 38.72 (C*Me*_3_), 45.62 (N(CH_2_)_2_), 52.49 (N(CH_2_)_2_), 115.22 (C-2′, C-6′), 119.87 (C-6″), 120.46 (C-3″), 122.72 (q, *J* = 274 Hz, CF3), 123.23 (C-4′), 124.32 (C-4″), 125.43 (q, *J* = 31.6 Hz, C-3), 125.84 (C-5″), 126.10 (C-5), 129.78 (C-3′, C-5′), 130.48 (q, *J* = 4.9 Hz, C-4), 131.93 (C-1), 133.26 (C-1″), 135.40 (C-6), 140.73 (C-2″), 149.68 (q, *J* = 1.9 Hz, C-2), 154.14 (C-1′), 161.73 (C=O), 176.50 ((C=O)NH); HRMS (ESI +) calcd for C_29_H_31_F_3_N_3_O_3_ [M+H]^+^: 525.2318; found: 526.2309.

### 3.3. Biological Tests

#### 3.3.1. In Vitro Microplate Assay against *P. falciparum* NF54

In vitro activity against erythrocytic stages of *P. falciparum* was determined with a ^3^H-hypoxanthine incorporation assay [42,43], using the chloroquine sensitive NF54 strain [44]. Chloroquine (Sigma C6628) was used as standard. Test compounds were dissolved in DMSO at 10 mg/mL and added to parasite cultures incubated in RPMI 1640 medium without hypoxanthine, supplemented with HEPES (5.94 g/L, NaHCO_3_ (2.1 g/L), neomycin (100 U/mL), Albumax (5 g/L) and washed human red blood cells A^+^ at 2.5% hematocrit (0.3% parasitemia). Serial drug dilutions of 11 3-fold dilution steps (covering a range from 100–0.002 µg/mL) were prepared. The 96-well plates were incubated at a humidified atmosphere at 37 °C; 4% CO_2_, 3% O_2_, 93% N_2_. After 48 h, 0.05 mL of ^3^H-hypoxanthine (=0.5 µCi) was added to each well. The plates were incubated for another 24 h under the same conditions. Then, the plates were harvested with a Betaplate cell harvester (Wallac, Zurich, Switzerland). The red blood cells were transferred onto glass fiber filter and washed with distilled water. The dried filters were inserted into a plastic foil with 10 mL of scintillation fluid and counted in a Betaplate liquid scintillation counter. IC_50_ values were determined from sigmoidal inhibition curves by linear regression using Microsoft Excel [45]. Chloroquine was used as control.

#### 3.3.2. In Vitro Cytotoxicity with L-6 Cells

In vitro cytotoxicity was determined using a primary cell line of rat skeletal myofibroblasts. The assay was performed in 96-well microtiter plates, each well containing 0.1 mL of RPMI 1640 medium, supplemented with 0.1% L-glutamine (200 mM) and 10% fetal bovine serum, as well as 4000 L-6 cells [46,47]. Serial drug dilutions of 11 3-fold dilution steps (covering a range from 100–0.002 µg/mL) were prepared. After 70 h of incubation time, the plates were inspected under an inverted microscope to ensure sterile conditions and growth of the controls. Then, 0.01 mL of Alamar Blue was added to each well and the plates were incubated for another 2 h. After that, the plates were read with a SpectraMax Gemini XS microplate fluorometer (Molecular Devices Corporation, Sunnyvale, CA, USA) using an excitation wavelength of 536 nm and an emission wavelength of 588 nm. IC_50_ values were determined by linear regression from the sigmoidal dose inhibition curves using SoftmaxPro software (Molecular Devices Corporation, Sunnyvale, CA, USA) [45]. Podophyllotoxin (Sigma P4405) was used as control.

#### 3.3.3. Parallel Artificial Membrane Permeability Assay

The PAMPA was performed using a Corning Gentest Pre-coated PAMPA Plate System at a pH of 7.4. It consists of 96-well polystyrene plates, whereas the donor-plate (bottom plate) is a conventional 96-well microtiter plate. The base of the acceptor-plate (top plate) consists of a porous membrane. The pores are lined with a lipid-oil-lipid trilayer. Stock solutions (10 mM) of each test compound were prepared in DMSO or *Me*OH and then further diluted to a final concentration of 200 µM with phosphate-buffered saline (PBS) at a pH of 7.4. Compound solutions were then added to the wells of the donor plate and pure PBS was added to each well of the acceptor plate. Compounds and negative control (pure PBS) were tested in quadruplicates. Donor and acceptor plate were coupled and incubated at ambient temperature for 5 h. After that, the plates were separated and solutions from each well of both plates were transferred onto 96-well UV-Star Microplates (Greiner Bio-One). The UV-absorption was measured at different wavelengths (between 200 and 300 nm) by a SpectraMax M3 UV plate reader (Molecular Devices). The concentrations were received from a calibration curve for each substance. The plates were analyzed at a wavelength were the R^2^ value of the calibration curve was higher than 0.99 [48]. Hydrochlorothiazide (*P_e_* = 0.90) and caffeine (*P_e_* = 80.00) were used as standards. The effective permeability (*P_e_*) was calculated as shown in the following Equations (1)–(3):

(1) $$Pe\;\left(nm/s\right)=\frac{-\ln\left[1-\frac{c_{A}\left(t\right)}{c_{equ}}\right]}{S*\left(\frac{1}{V_{D}}+\frac{1}{V_{A}}\right)*t}$$

where:

*P_e_*—effective permeability;
*S—*filter area (0.3 cm^2^);
*V_D_*—donor well volume;
*V_A_*—acceptor well volume;
*t*—incubation time (18,000 s);
*c_A_*(*t*)*—*compound concentration in acceptor well at time *t*;
*c_equ_*—equilibrium concentration.

(2) $$c_{equ}=\frac{\left[c_{D}\left(t\right)*\;V_{D}+c_{A}\left(t\right)*\;V_{A}\right]}{\left(V_{D}+V_{A}\right)}$$

where:

*c_D_*(*t*)—compound concentration in donor well at time *t*.

Recovery of compounds from donor and acceptor wells (mass retention) was calculated as shown in the equation below. Data were only accepted when recovery exceeded 70%.

(3) $$R=1-\frac{\left[c_{D}\left(t\right)*\;V_{D}+c_{A}\left(t\right)*\;V_{A}\right]}{\left(c_{0}*\;V_{D}\right)}$$

where:

*R*—mass retention (%);
*c_A_*(*t*)—compound concentration in acceptor well at time *t*;
*c_D_*(*t*)—compound concentration in donor well at time *t*;
*c*_0_—initial compound concentration in donor well;
*V_D_*—donor well volume;
*V_A_*—acceptor well volume.

#### 3.3.4. Cytochrom P450 3A4 Inhibition Assay

The CYP3A4 inhibition assay was performed using 96-well White Plates (Greiner Bio-One) at a pH of 7.4. Stock solutions (4 mM) of test compounds were prepared in DMSO, stock solution of the standard ketoconazole (5 mM) was prepared in acetonitrile. Stock solutions were further diluted to a final concentration of 20 µM using water (HPLC grade). The luciferin IPA stock solution (3 mM) was diluted to a final concentration of 0.3 mM using water (HPLC grade). The CYP3A4 reaction mixture was prepared by mixing water (HPLC grade) with potassium phosphate buffer (1 M), luciferin IPA (0.3 mM) and CYP3A4 membrane (1 pmol/µL). The control reaction mixture was prepared using water (HPLC grade), potassium phosphate buffer (1 M), luciferin IPA (0.3 mM) and membrane without CYP activity (1 pmol/µL). Solutions A and B of the NADPH regeneration system were mixed and HPLC grade water was added. The reconstituted luciferin detection reagent was prepared by mixing the reconstituted buffer with esterase with the luciferin detection reagent. Then, solutions of test compounds and standard were added to the wells of the 96-well White Plate, each was tested in triplicate. The CYP3A4 reaction mixture was added to each well and the plate was incubated for 10 min at room temperature. After that, the NADPH regeneration system was added, inducing the reaction followed by an incubation time of 10 min at ambient temperature. By adding the reconstituted luciferin detection reagent, the reaction was terminated, and a luminescent signal was formed. Luminescence was measured by a SpectraMax M3 UV plate reader (Molecular Devices). The relative light units (RLU) were received from a calibration curve with beetle luciferin. Ketoconazole (100% enzyme inhibition) was used as standard [49]. The CYP3A4 inhibition (%) was calculated from the RLU.

#### 3.3.5. Ligand Efficiency (LE)

Ligand efficiency was calculated as shown in the following Equation (4) [31]:

(4) $$\mathrm{LE}=\frac{1.37}{\mathrm{HA}}*{\mathrm{pIC}}_{50}$$

where:

LE—ligand efficiency;
HA—number of heavy atoms;
pIC_50_—negative logarithm of IC_50_.

## 4. Conclusions

This paper deals with the synthesis, antiplasmodial activities and first insights into structure-activity relationships of a series of new 2-phenoxybenzamides. The lead compound MMV030666 from MMV’s Malaria Box Project is a 2-(4-fluorophenoxy)benzanilide with a 4-(*N-Boc*)piperazinyl substituent in *ortho* position of the anilide nitrogen. It is of particular interest as it exhibits multi-stage activity against different strains of *P. falciparum*. Moreover, development of resistant parasites could not be observed in long-term in vitro studies with sub-lethal doses. We aimed on synthesizing derivatives to gain first insights into structure-activity relationships. Our main focus was put on derivatization of the anilino moiety.

**Figure 8:**
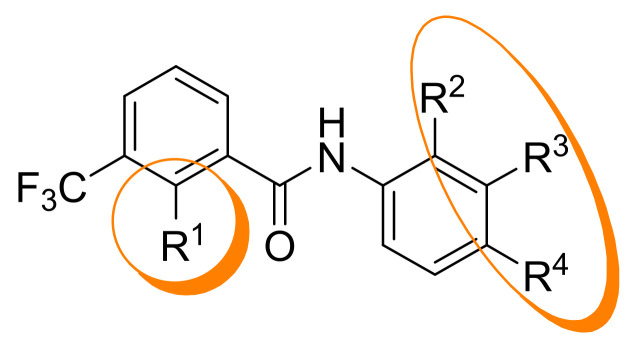
Positions of structural modifications.

Modifications in Figure 8 showed the great importance of the phenoxy substituent as well as the beneficial effect of its 4-fluoro substitution. Replacement of the piperazinyl moiety by a hydrogen atom or an amino group led to inactive compounds. Substitution of the *N-Boc* group usually caused a considerable decrease in activity, but compounds with a *N*-pivaloyl group showed comparable activity. Their enhanced stability in acidic environment could be of advantage. Moreover, the ring position of the piperazinyl substituent was of particular importance. *Meta* substituted derivatives were only moderately active. The most active compounds had a 4-(*N-Boc*)piperazinyl or a 4-(*N*-pivaloyl)piperazinyl substituent in position 2 or 4 of the anilide nitrogen (Table 4).

## Figures and Tables

**Table 1:** Activities of compounds **1**, **6**–**8**, **12**–**14**, **17**–**22**, **36**–**39**, **50**, **51** and **54**–**56** against *P. falciparum* NF54 and L-6 cells, expressed as IC_50_ (µM) ^a^.

Compound	*P.f.* NF54 ^b^ IC_50_ (µM)	S.I. = IC_50_ (Cyt.)/IC_50_ (*P.f.* NF54)	Cytotoxicity L-6 Cells IC_50_ (µM)
**1**	0.4134	316.9	131.0
**6**	1.012	127.1	128.3
**7**	3.738	30.22	113.0
**8**	1.146	62.93	73.00
**12**	9.325	21.71	202.5
**13**	1.902	9.043	17.20
**14**	21.28	6.080	129.4
**17**	2.533	10.72	27.12
**18**	6.585	4.829	31.80
**19**	0.6172	299.7	185.0
**20**	2.890	12.01	34.72
**21**	15.64	2.265	35.43
**22**	2.300	8.770	20.17
**36**	3.297	37.58	124.0
**37**	0.2690	461.0	124.0
**38**	3.174	24.61	78.00
**39**	0.5795	171.9	99.62
**50**	51.49	2.026	104.3
**51**	55.85	2.328	130.0
**54**	1.222	151.4	184.7
**55**	4.662	19.27	89.81
**56**	0.6593	288.6	190.3
CQ	0.009	9672	90.92
POD			0.012

CQ = chloroquine; POD = podophyllotoxin. ^a^ Values represent the average of four determinations (two determinations of two independent experiments); ^b^ sensitive to chloroquine.

**Table 2:** Key physicochemical parameters of compounds **1**, **6**–**8**, **12**–**14**, **17**–**22**, **36**–**39**, **50**, **51** and **54**–**56**.

Compound	Log P ^a^	Log D_7_._4_ ^a^	LE (Kcal/Mol/HA)
**1**	6.44	6.44	0.219
**6**	6.30	6.30	0.211
**7**	4.80	4.80	0.232
**8**	5.54	5.54	0.198
**12**	5.59	5.59	0.255
**13**	6.50	6.50	0.224
**14**	4.76	4.76	0.229
**17**	4.77	4.77	0.213
**18**	4.72	4.72	0.203
**19**	6.57	6.57	0.218
**20**	6.60	5.48	0.205
**21**	4.43	4.43	0.183
**22**	4.88	4.88	0.203
**36**	6.44	6.44	0.188
**37**	6.44	6.44	0.225
**38**	6.57	6.57	0.193
**39**	5.56	5.56	0.236
**50**	4.76	4.76	0.210
**51**	4.76	4.76	0.208
**54**	6.30	6.30	0.208
**55**	6.42	6.42	0.187
**56**	6.42	6.42	0.217

^a^ log P and log D were calculated using the ChemAxon software JChem for Excel 14.9.1500.912 (2014).

**Table 3:** Passive permeability and CYP3A4 inhibition values of compounds **1**, **6**–**8**, **12**–**14**, **17**–**22**, **36**–**39**, **50**, **51** and **54**–**56**.

Compound	Pe ^a^ (10^−6^ cm/s)	CYP3A4 Inhibition ^b^ (%)
**1**	2.37	87
**6**	3.00	87
**7**	0.08	
**8**	4.06	99
**12**	10.09	
**13**	8.72	90
**14**	11.41	
**17**	10.12	
**18**	1.05	82
**19**	0.23	94
**20**	2.48	
**21**	7.44	
**22**	n.d.	
**36**	6.17	96
**37**	0.09	93
**38**	1.17	
**39**	0.24	89
**50**	18.38	
**51**	12.94	88
**54**	1.22	80
**55**	11.15	60
**56**	0.11	

^a^ determined by PAMPA, n.d.: could not be determined; ^b^ determined by Cytochrom P450 3A4 inhibition assay.

**Table 4:** Summary of SAR of compounds against *P. falciparum* NF54 expressed as IC_50_ (µM).

R^1^	R^2^	R^3^	R^4^	*P.f.* NF54 IC_50_ (µM)
4-fluorophenoxy	H	H	*N-*pivaloylpiperazinyl	0.2690
4-fluorophenoxy	*N-Boc*-piperazinyl	H	H	0.4134
4-fluorophenoxy	H	H	*N-*pivaloy*l*piperazinyl	0.5795
4-fluorophenoxy	*N*-pivaloylpiperazinyl	H	H	0.6172
phenoxy	*N*-pivaloylpiperazinyl	H	H	0.6593
phenoxy	*N*-*Boc*-piperazinyl	H	H	1.012
4-acetamidophenoxy	*N*-*Boc*-piperazinyl	H	H	1.146
phenoxy	H	H	*N*-*Boc*-piperazinyl	1.222
4-fluorophenoxy	*N*-carbamoyl-piperazinyl	H	H	1.902
4-fluorophenoxy	*N-*(dimethylcarbamoyl)piperazinyl	H	H	2.300
4-fluorophenoxy	*N*-acetylpiperazinyl	H	H	2.533
4-fluorophenoxy	*N*-*tert*-Butylpiperazinyl	H	H	2.890
4-fluorophenoxy	H	*N*-pivaloylpiperazinyl	H	3.174
4-fluorophenoxy	H	*N*-*Boc*-piperazinyl	H	3.297
H	*N-Boc*-piperazinyl	H	H	3.738
phenoxy	H	H	*N-*pivaloylpiperazinyl	4.662
4-fluorophenoxy	*N*-formylpiperazinyl	H	H	6.585
4-fluorophenoxy	H	H	H	9.325
4-fluorophenoxy	*N-*carbamoylpiperazinyl	H	H	15.64
4-fluorophenoxy	NH_2_	H	H	21.28
4-fluorophenoxy	H	NH_2_	H	51.49
4-fluorophenoxy	H	H	NH_2_	55.85

## Data Availability

The data presented in this study are available in this article.

## Supplementary Materials

The following are available online at https://www.mdpi.com/article/10.3390/ph14111109/s1, Figures S1–S40.
