# Supplementary material for: Synthesis and Structure-Activity Relationships of New 2-Phenoxybenzamides with Antiplasmodial Activity
Source: Pharmaceuticals (Basel). 2021 Oct 30;14(11):1109. doi: 10.3390/ph14111109 (PMC8625693; doi:10.3390/ph14111109)

Supplementary Information

Synthesis and Structure-Activity Relationships of new 2-Phenoxybenzamides with Antiplasmodial Activity

Theresa Hermann [1], Patrick Hochegger [1], Johanna Dolensky [1], Werner Seebacher [1], Eva-Maria Pferschy-Wenzig [2], Robert Saf [3], Marcel Kaiser [4, 5], Pascal Mäser [4, 5] and Robert Weis [1, *]

[1] Institute of Pharmaceutical Sciences, Pharmaceutical Chemistry, University of Graz, Schubertstraße 1, A-8010 Graz, Austria; theresa.hermann@uni-graz.at (T.H.); patrick.hochegger@uni-graz.at (P.H.); johanna.faist@uni-graz.at (J.D.); we.seebacher@uni-graz.at (W.S.)
[2] Institute of Pharmaceutical Sciences, Pharmacognosy, University of Graz, Beethovenstraße 8, A-8010, Graz, Austria; eva-maria.wenzig@uni-graz.at (E.P.-W.)
[3] Institute for Chemistry and Technology of Materials (ICTM), Graz University of Technology, Stremayrgasse 9, A-8010 Graz, Austria; robert.saf@tugraz.at (R.S.)
[4] Swiss Tropical and Public Health Institute, Socinstraße 57, CH-4002 Basel, Switzerland; marcel.kaiser@swisstph.ch (M.K.); pascal.maeser@swisstph.ch (P.M.)
[5] University of Basel, Petersplatz 1, CH-4001, Basel, Switzerland
* Correspondence: robert.weis@uni-graz.at; Tel.: +43-316-380-5379; Fax: +43-316-380-9846

NMR spectra data of compounds 1, 2, 6-9, 11-14, 17-22, 24-28, 31, 33-39, 42, 43, 47 and 49-56

Figure S1. 1H NMR at 400 MHz and 13C NMR at 100 MHz spectra for compound **1**

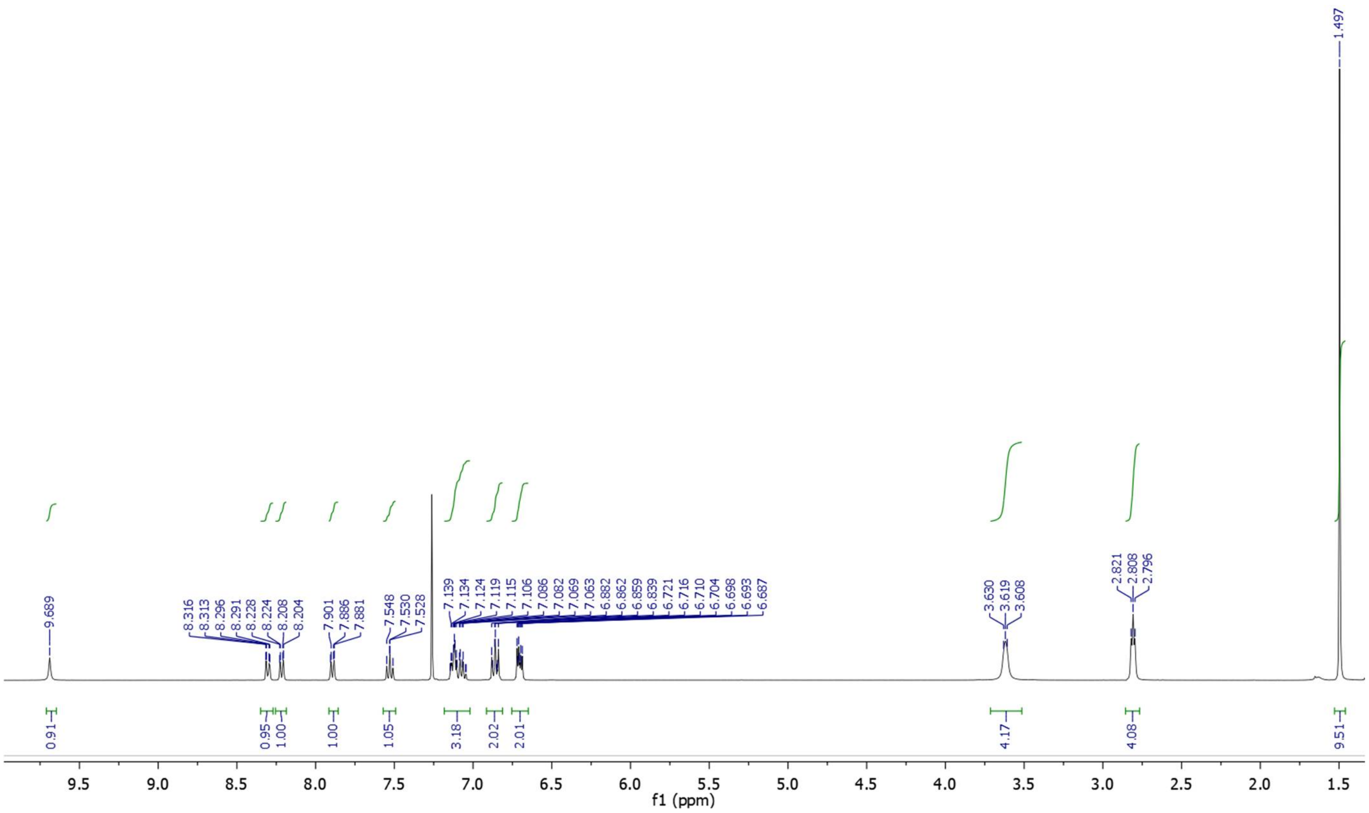

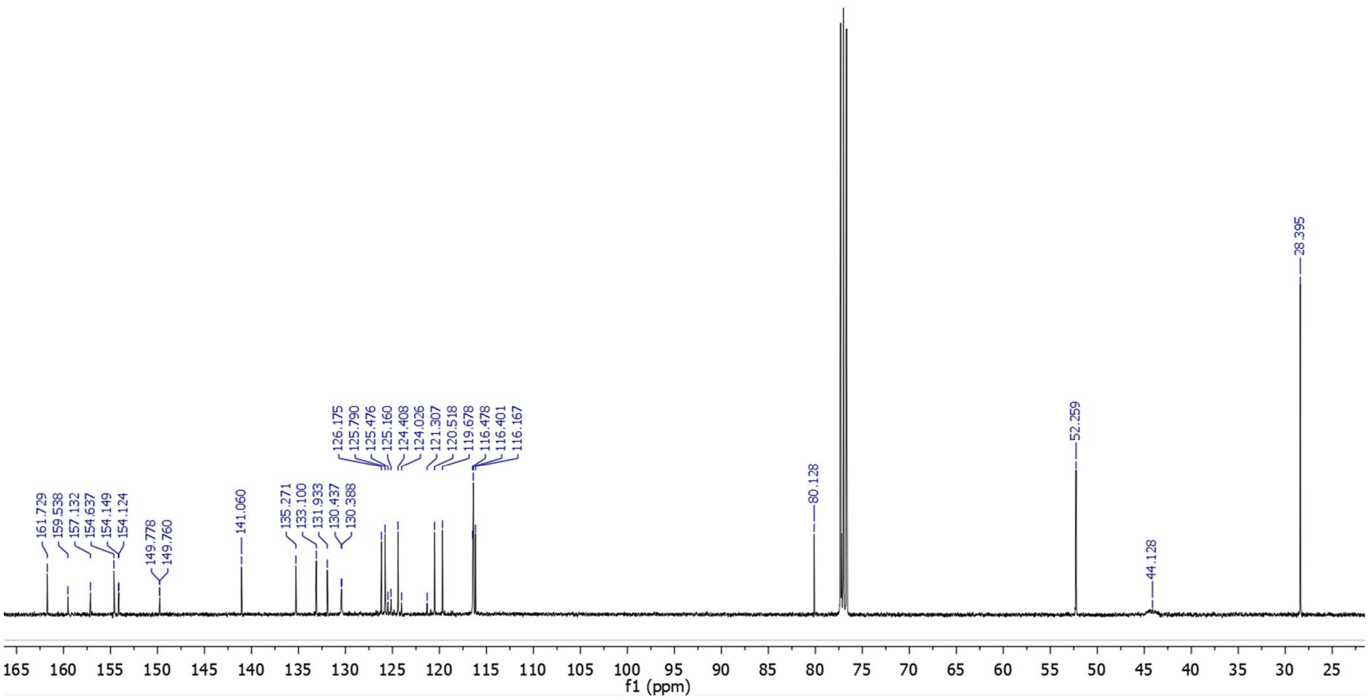

Figure S2. 1H NMR at 400 MHz and 13C NMR at 100 MHz spectra for compound **2**

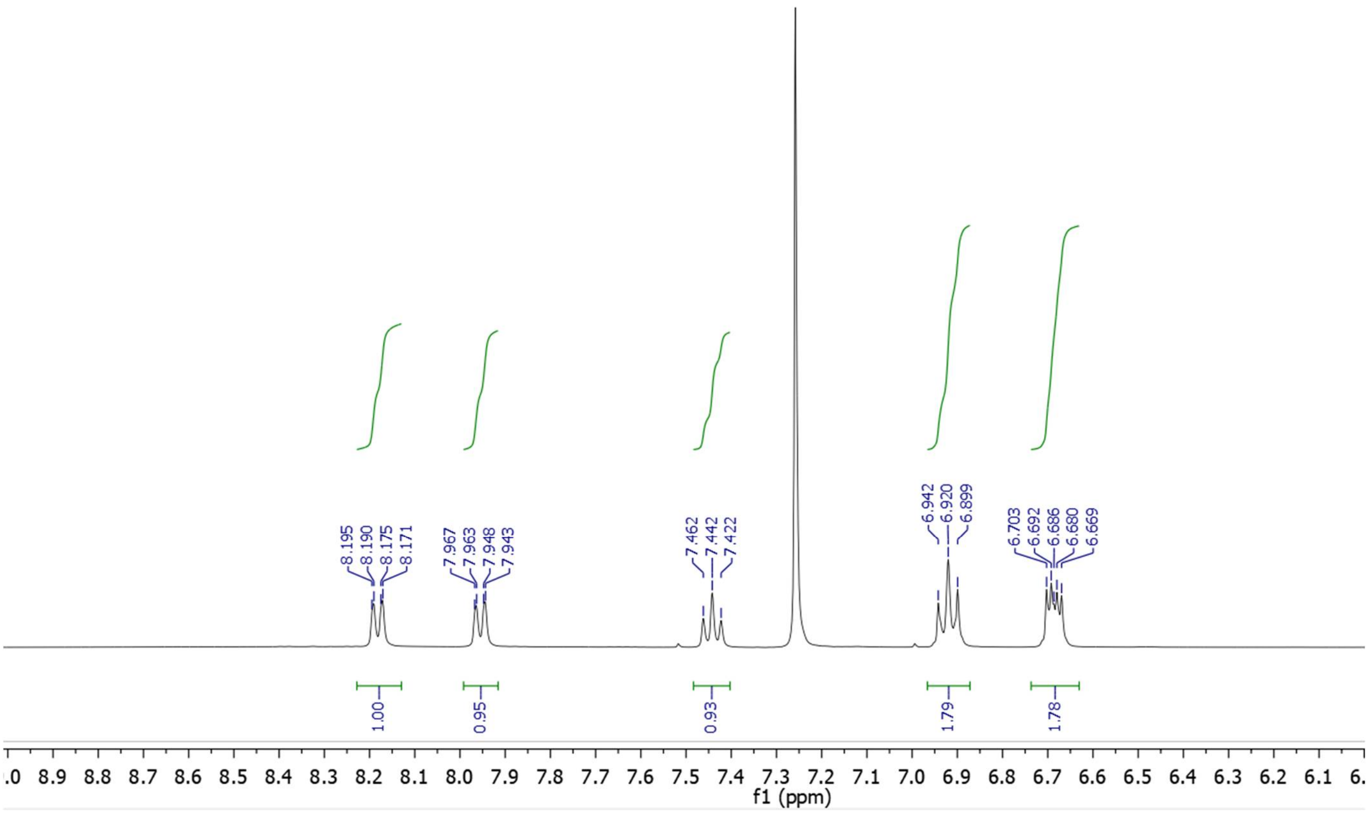

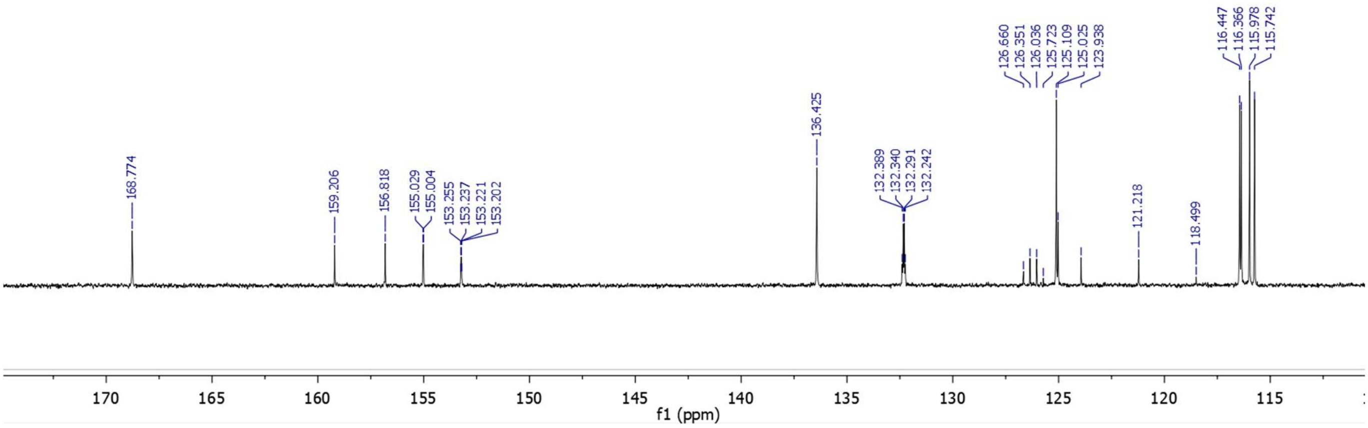

Figure S3. 1H NMR at 400 MHz and 13C NMR at 100 MHz spectra for compound **6**

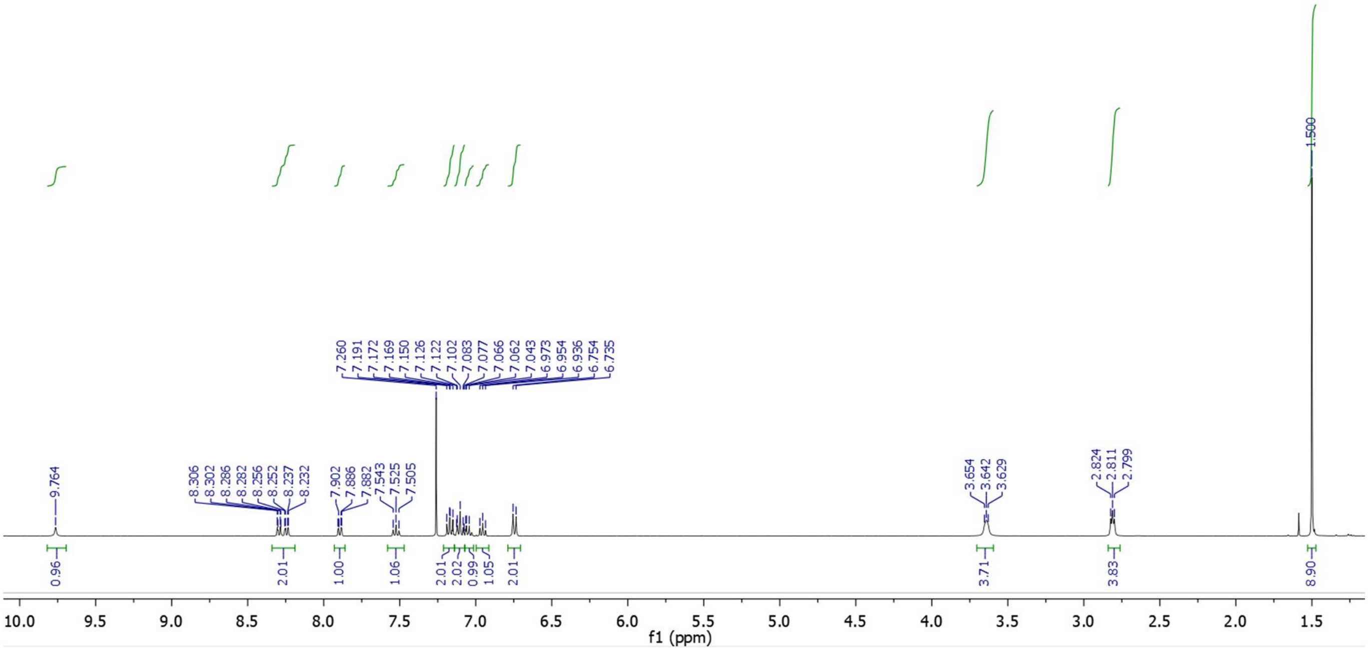

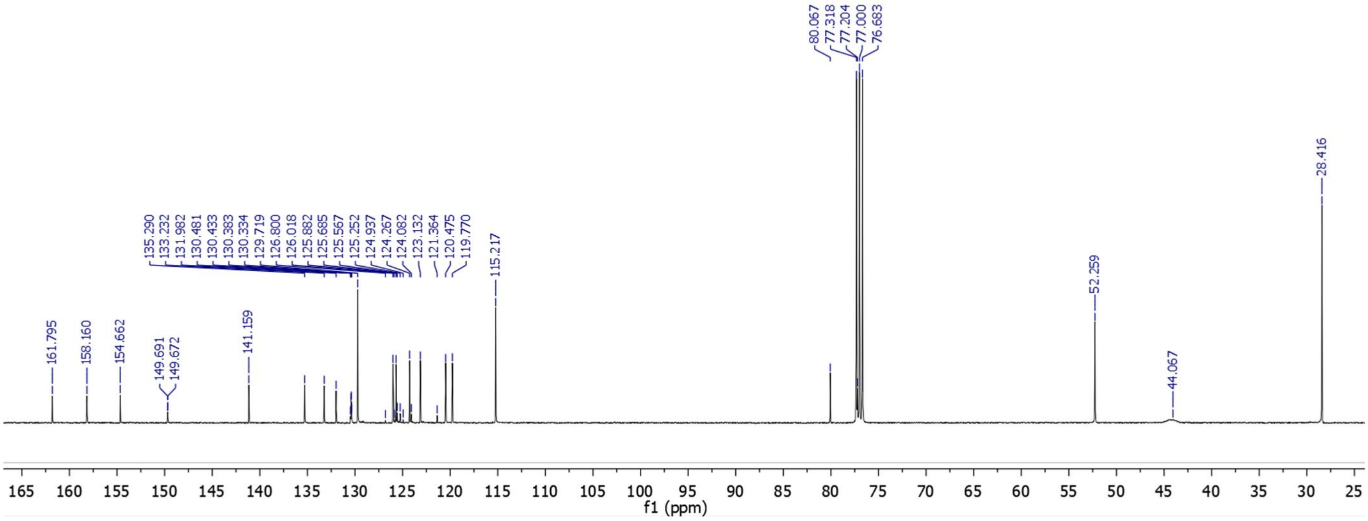

Figure S4. 1H NMR at 400 MHz and 13C NMR at 100 MHz spectra for compound **7**

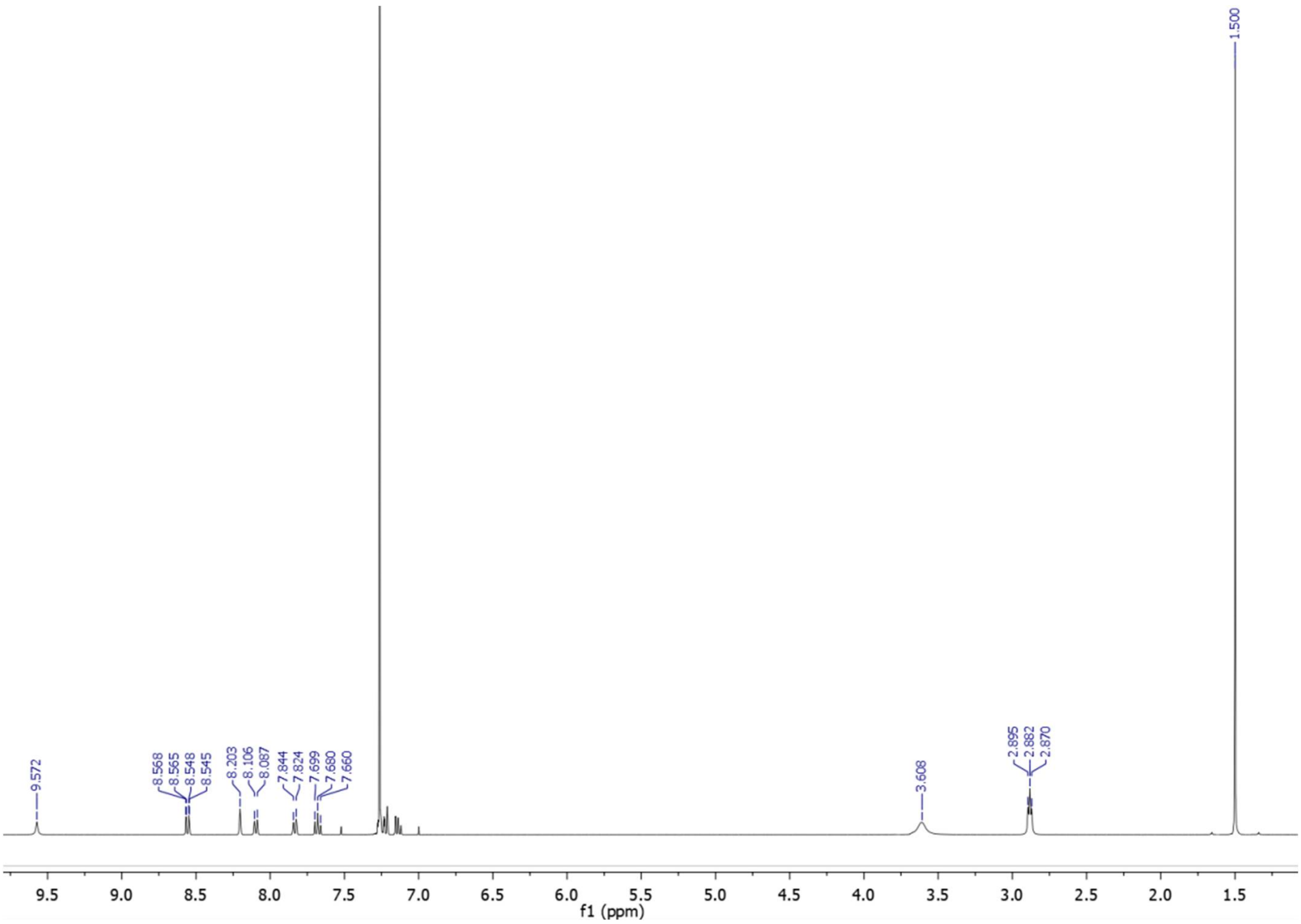

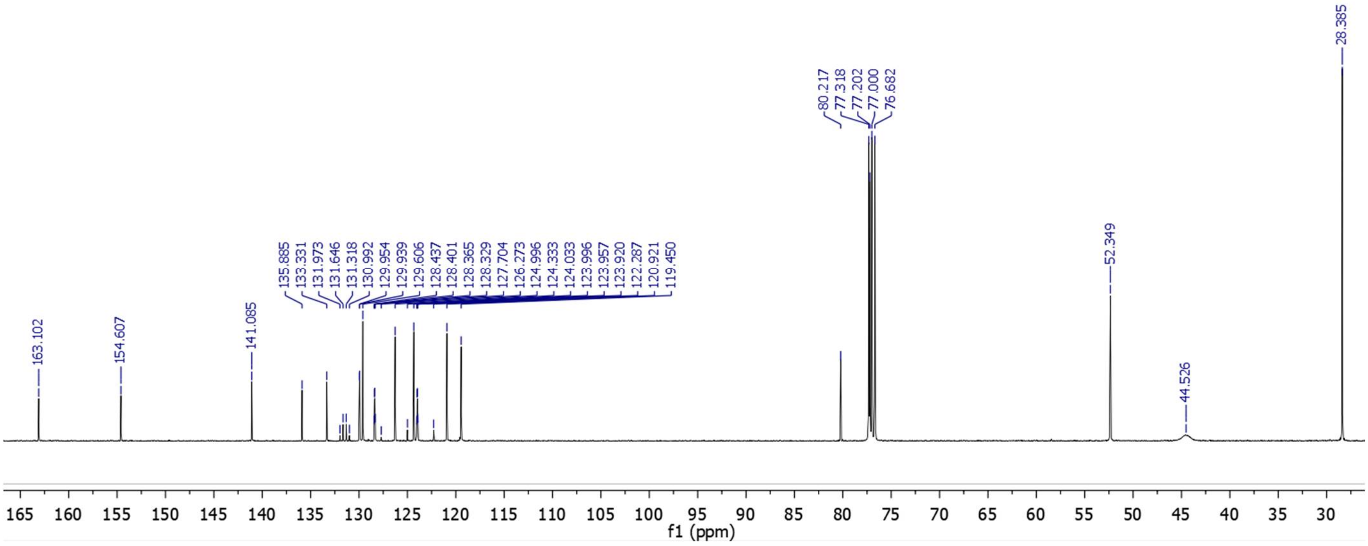

Figure S5. 1H NMR at 400 MHz and 13C NMR at 100 MHz spectra for compound **8**

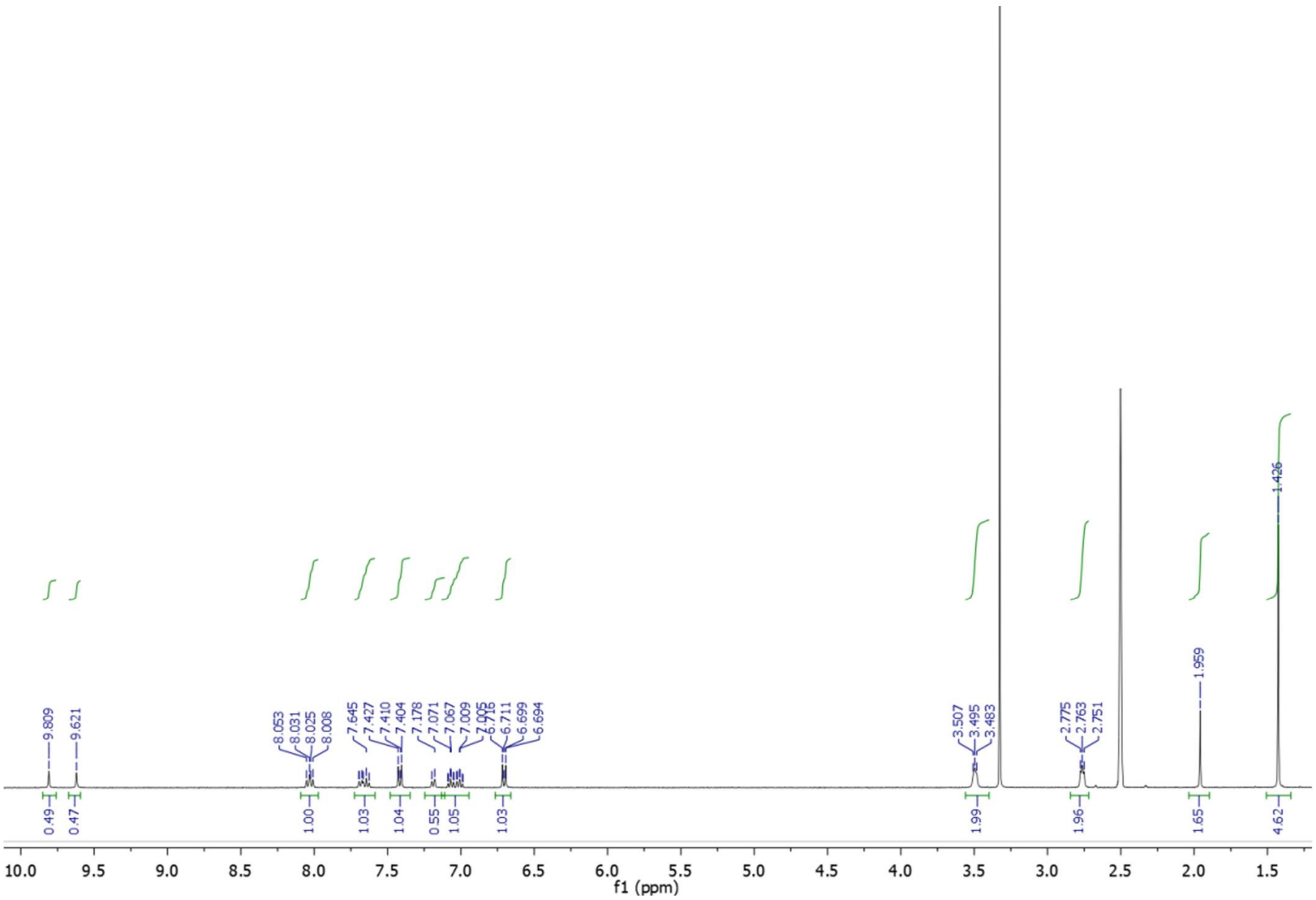

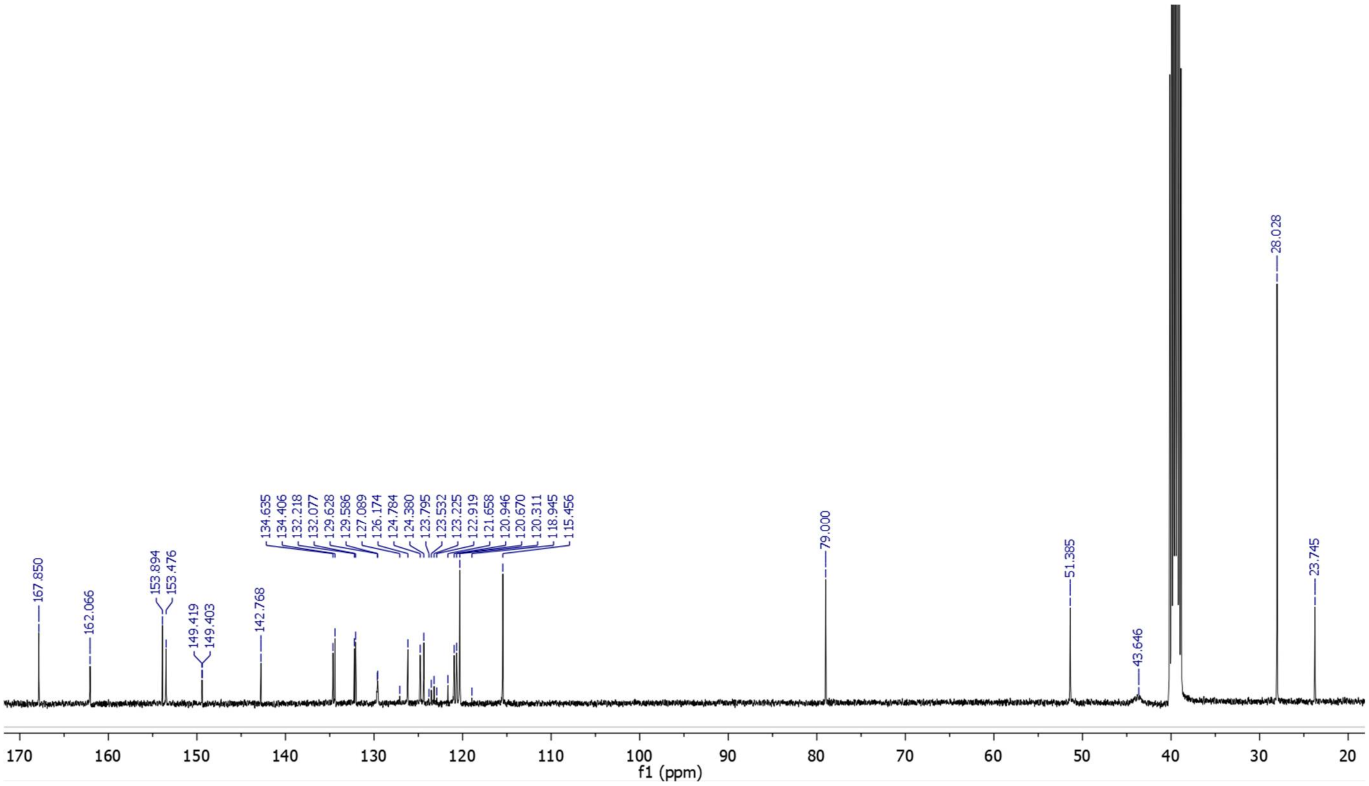

Figure S6. 1H NMR at 400 MHz and 13C NMR at 100 MHz spectra for compound **9**

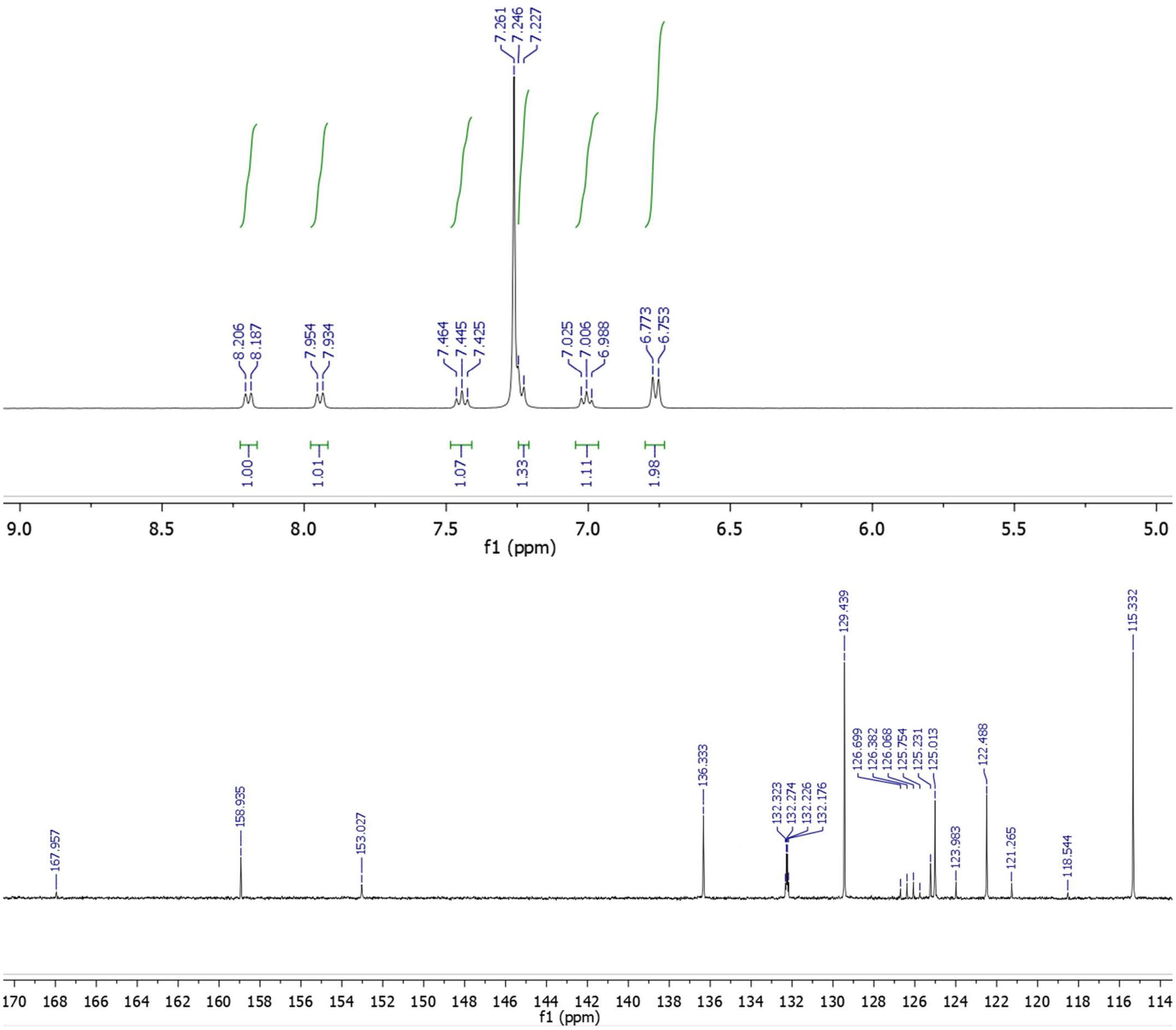

Figure S7. 1H NMR at 400 MHz and 13C NMR at 100 MHz spectra for compound **11**

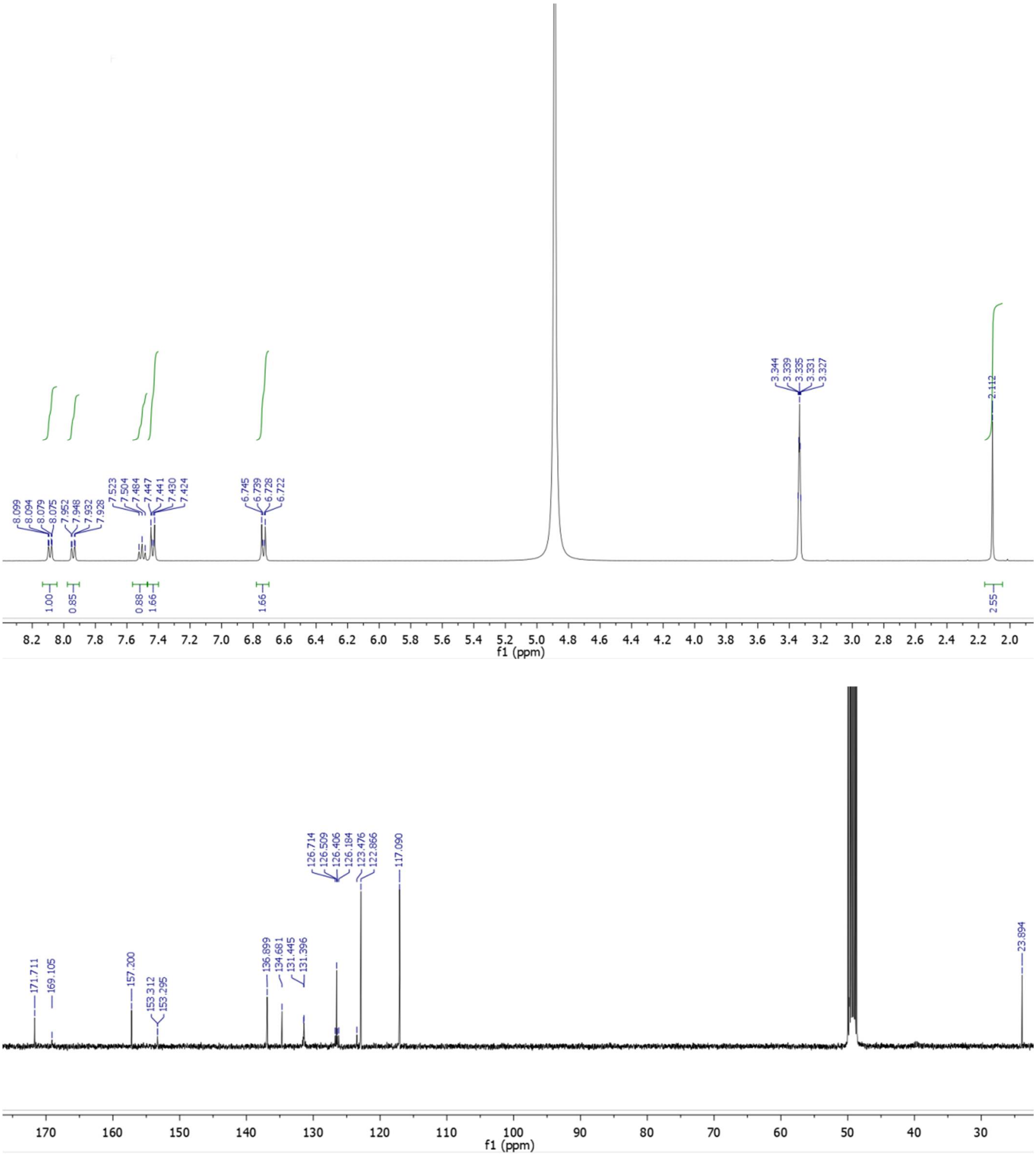

Figure S8. 1H NMR at 400 MHz and 13C NMR at 100 MHz spectra for compound **12**

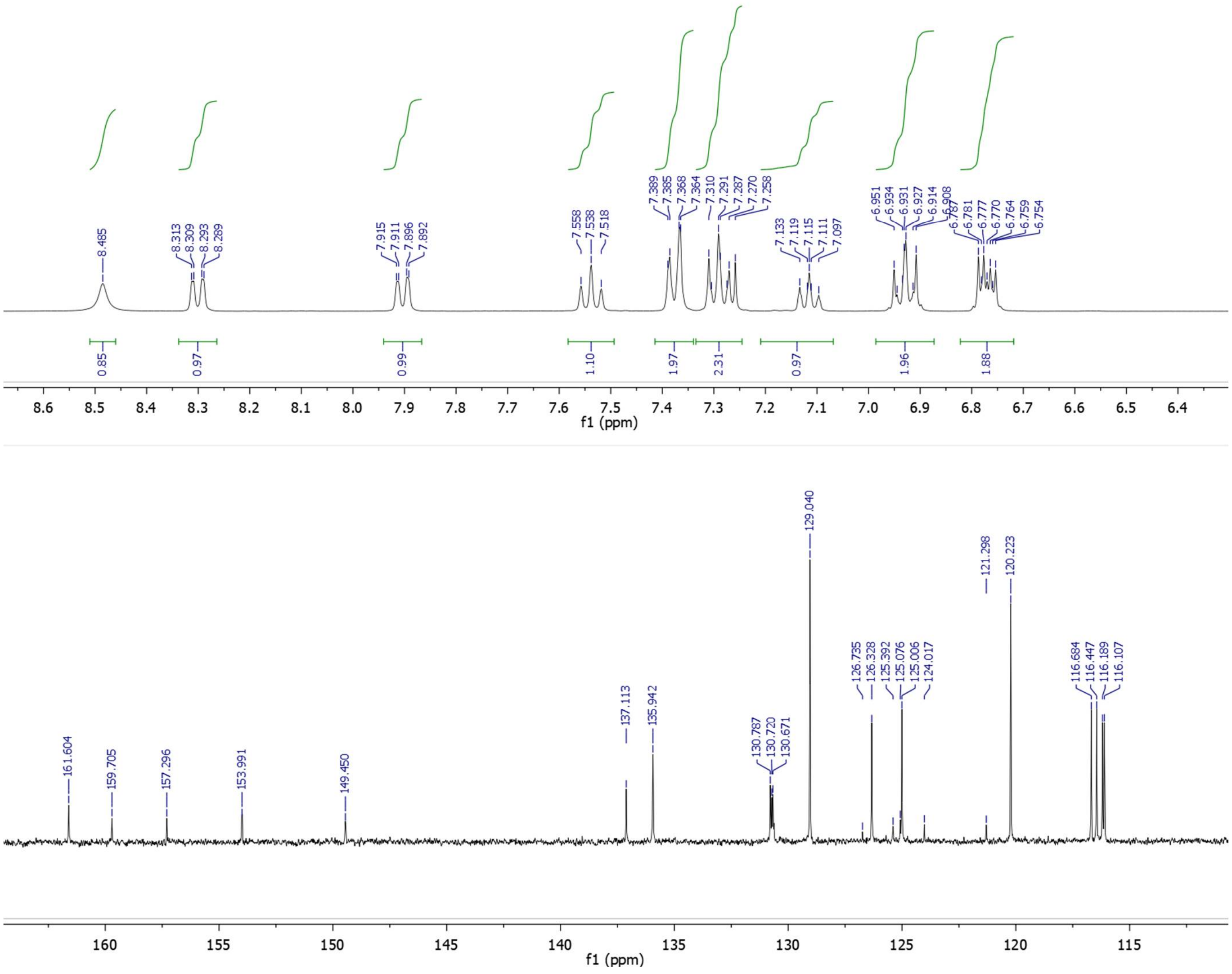

Figure S9. 1H NMR at 400 MHz and 13C NMR at 100 MHz spectra for compound **13**

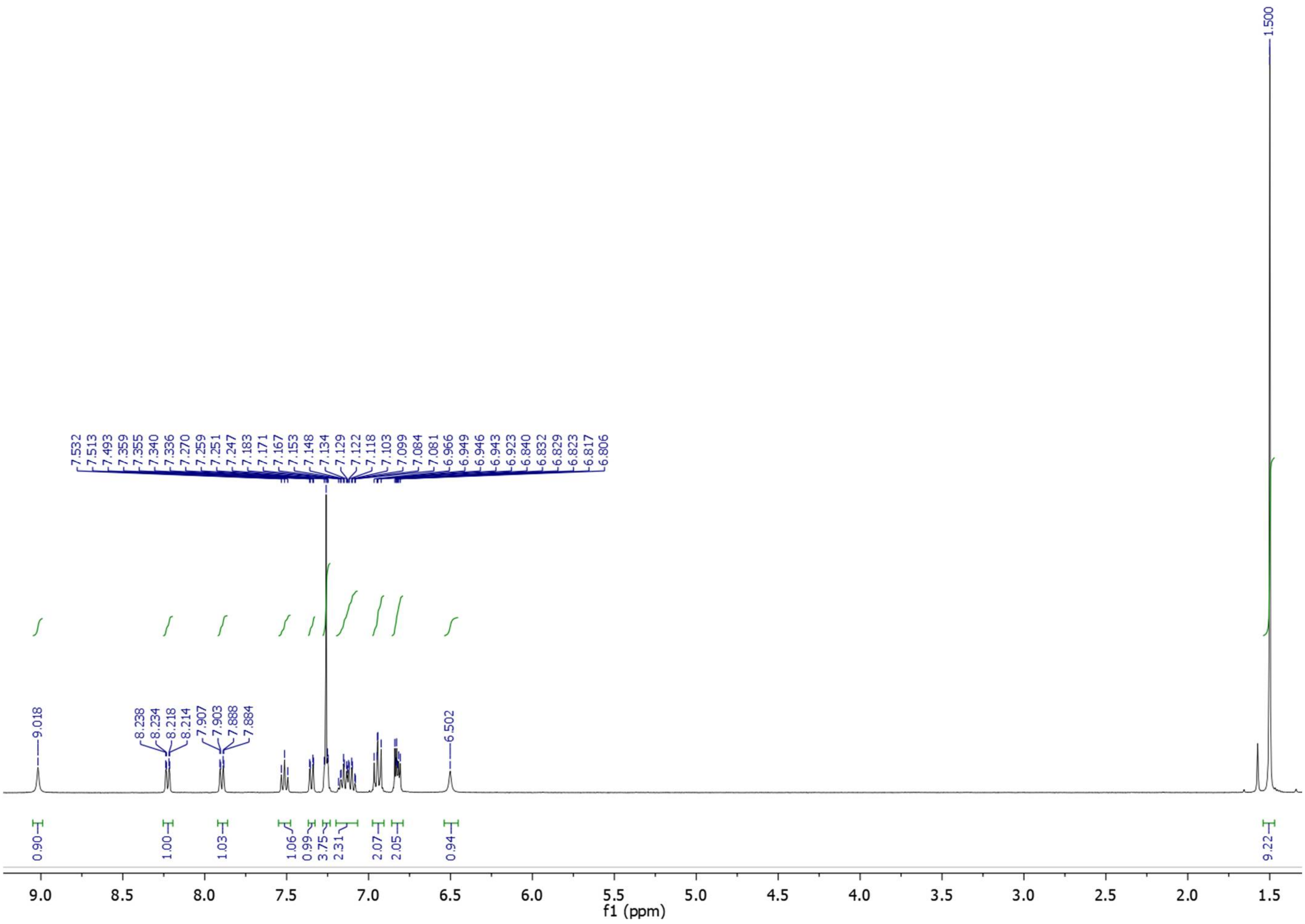

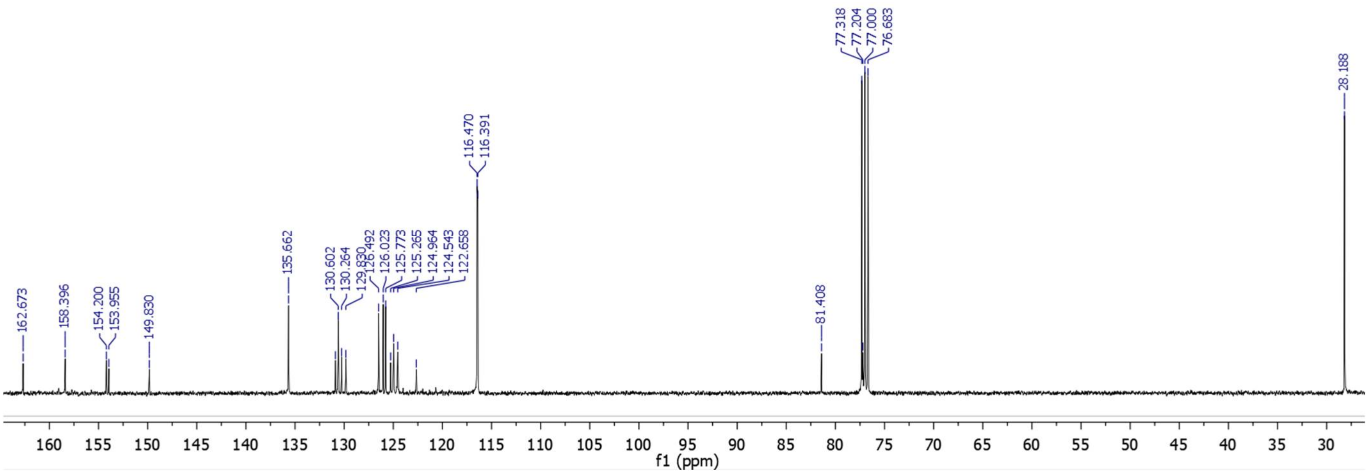

Figure S10. 1H NMR at 400 MHz and 13C NMR at 100 MHz spectra for compound **14**

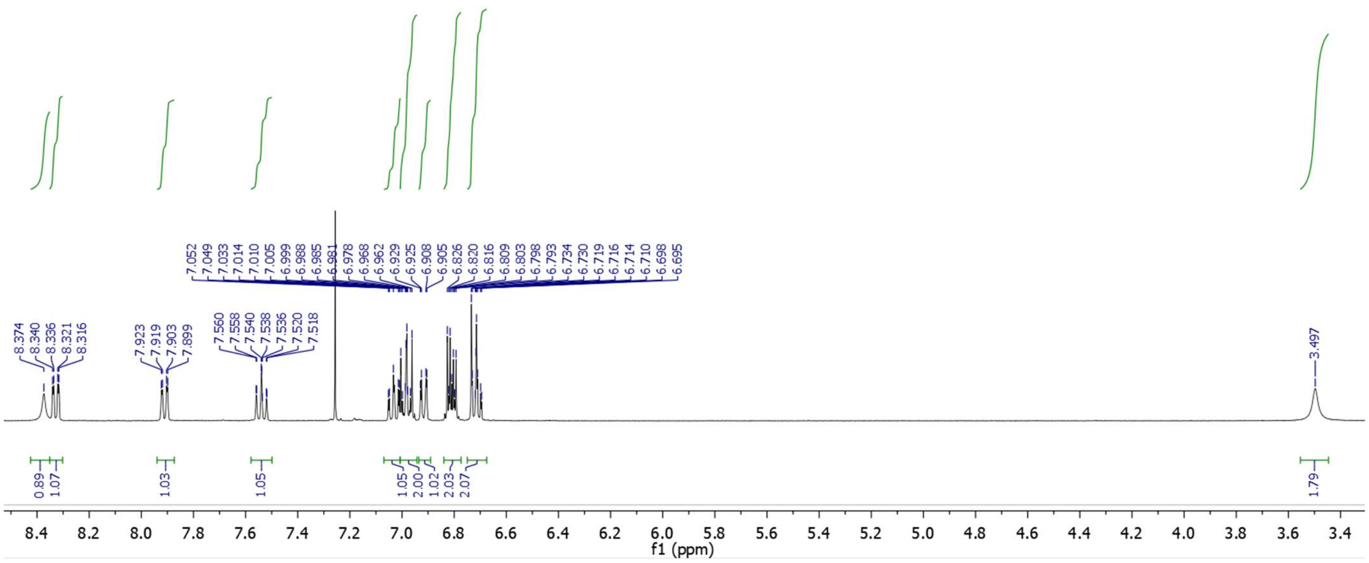

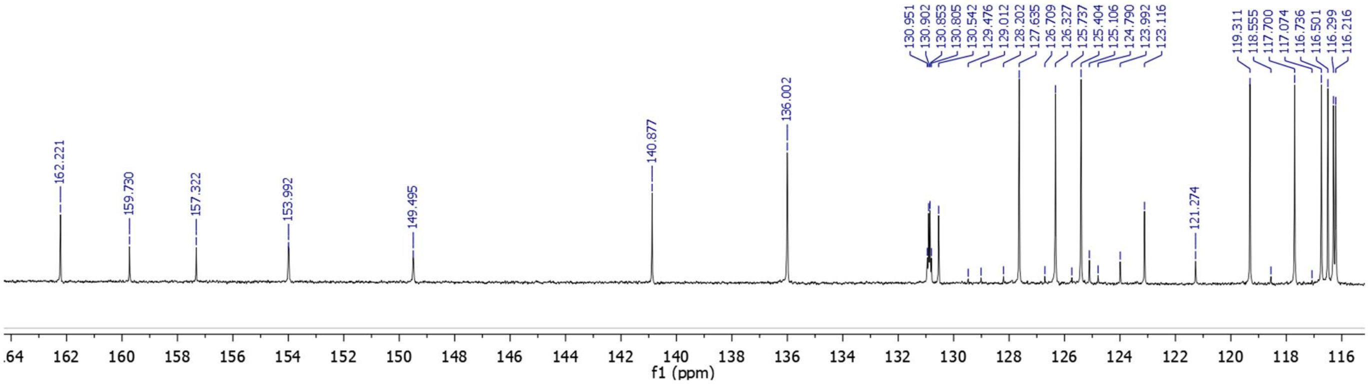

Figure S11. 1H NMR at 400 MHz and 13C NMR at 100 MHz spectra for compound **17**

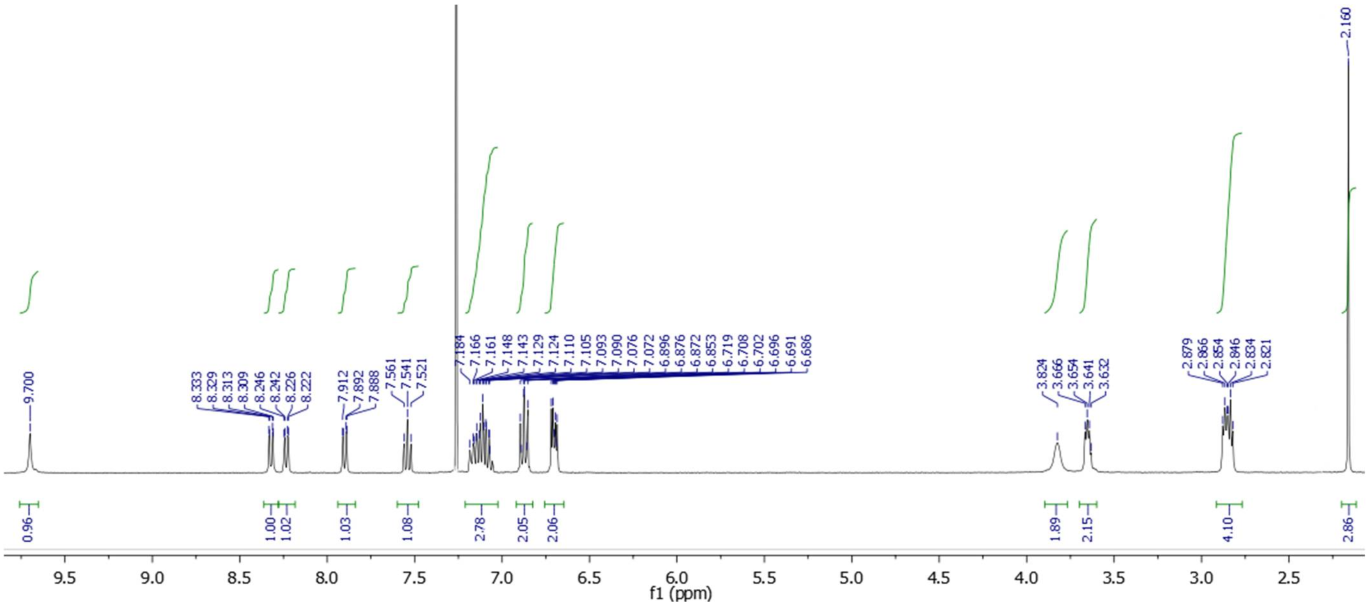

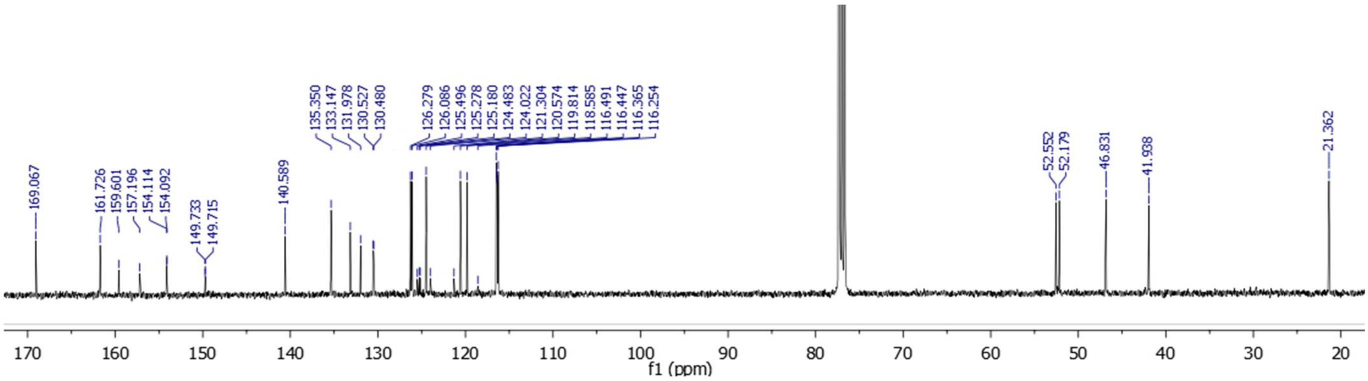

Figure S12. 1H NMR at 400 MHz and 13C NMR at 100 MHz spectra for compound **18**

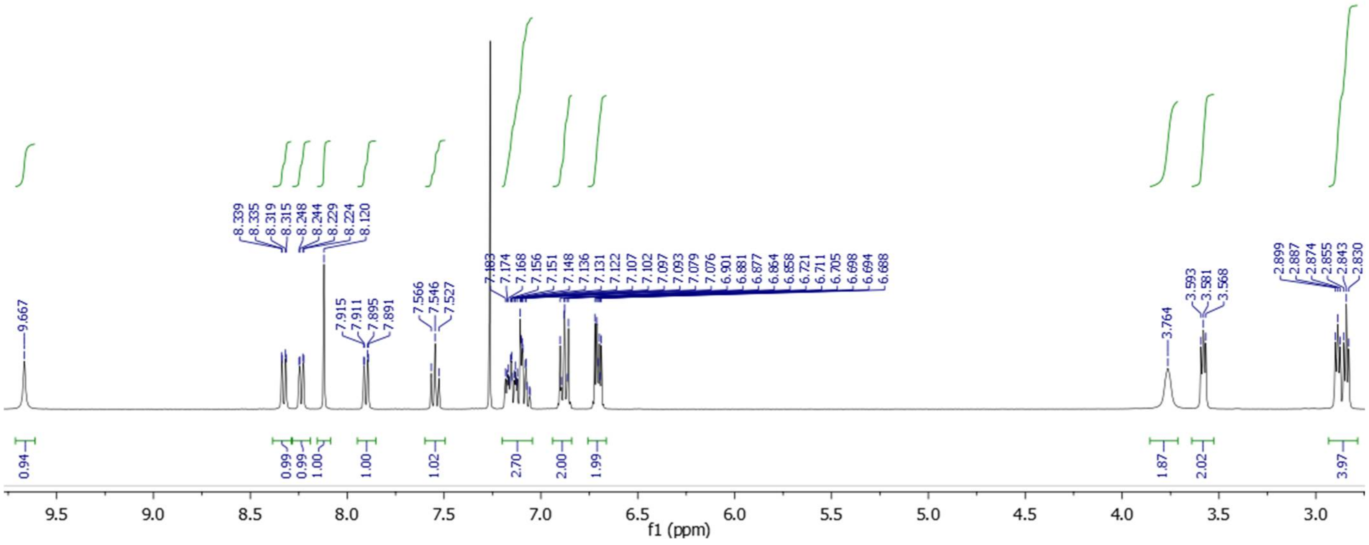

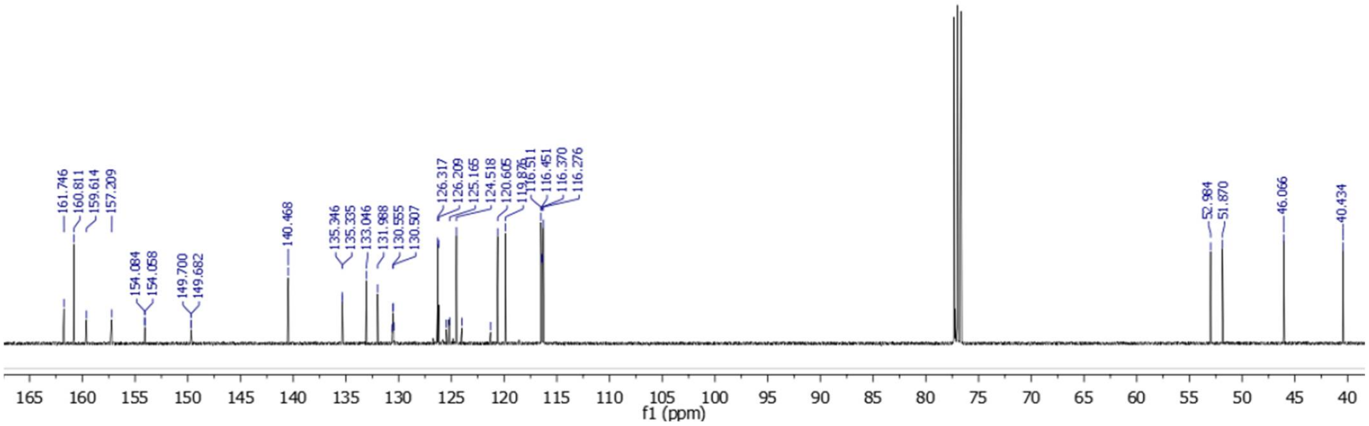

Figure S13. 1H NMR at 400 MHz and 13C NMR at 100 MHz spectra for compound **19**

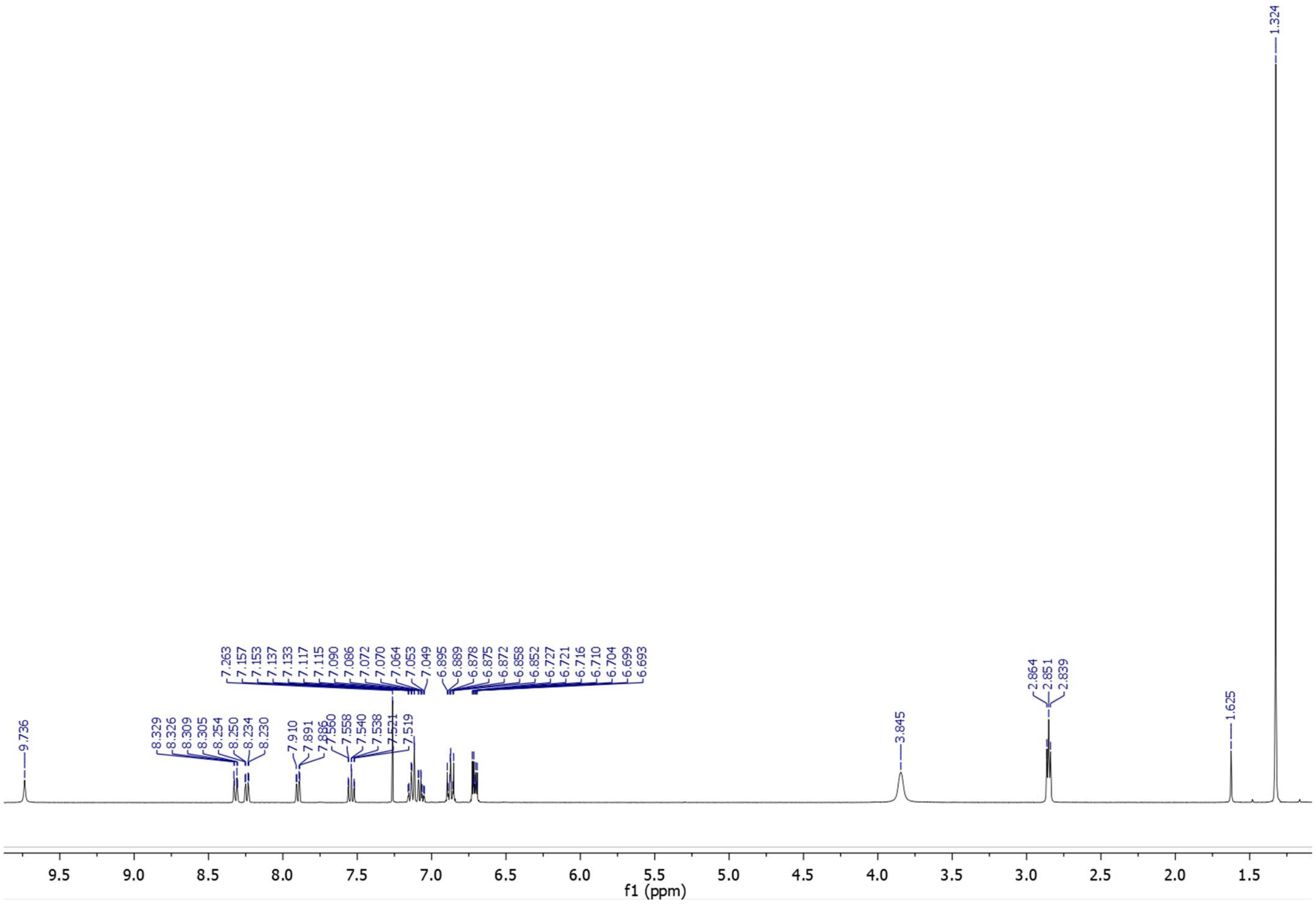

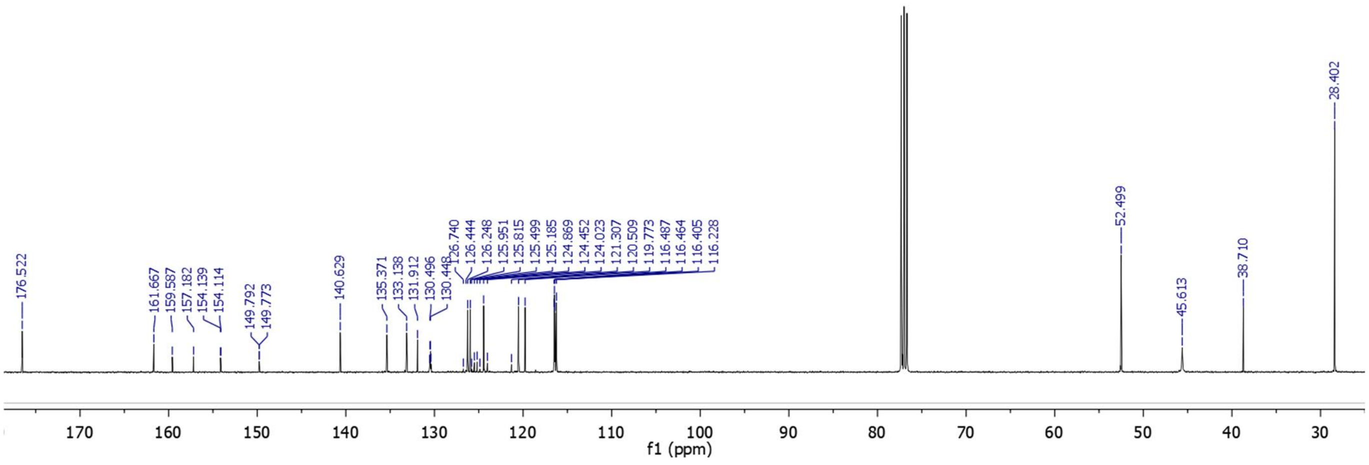

Figure S14. 1H NMR at 400 MHz and 13C NMR at 100 MHz spectra for compound **20**

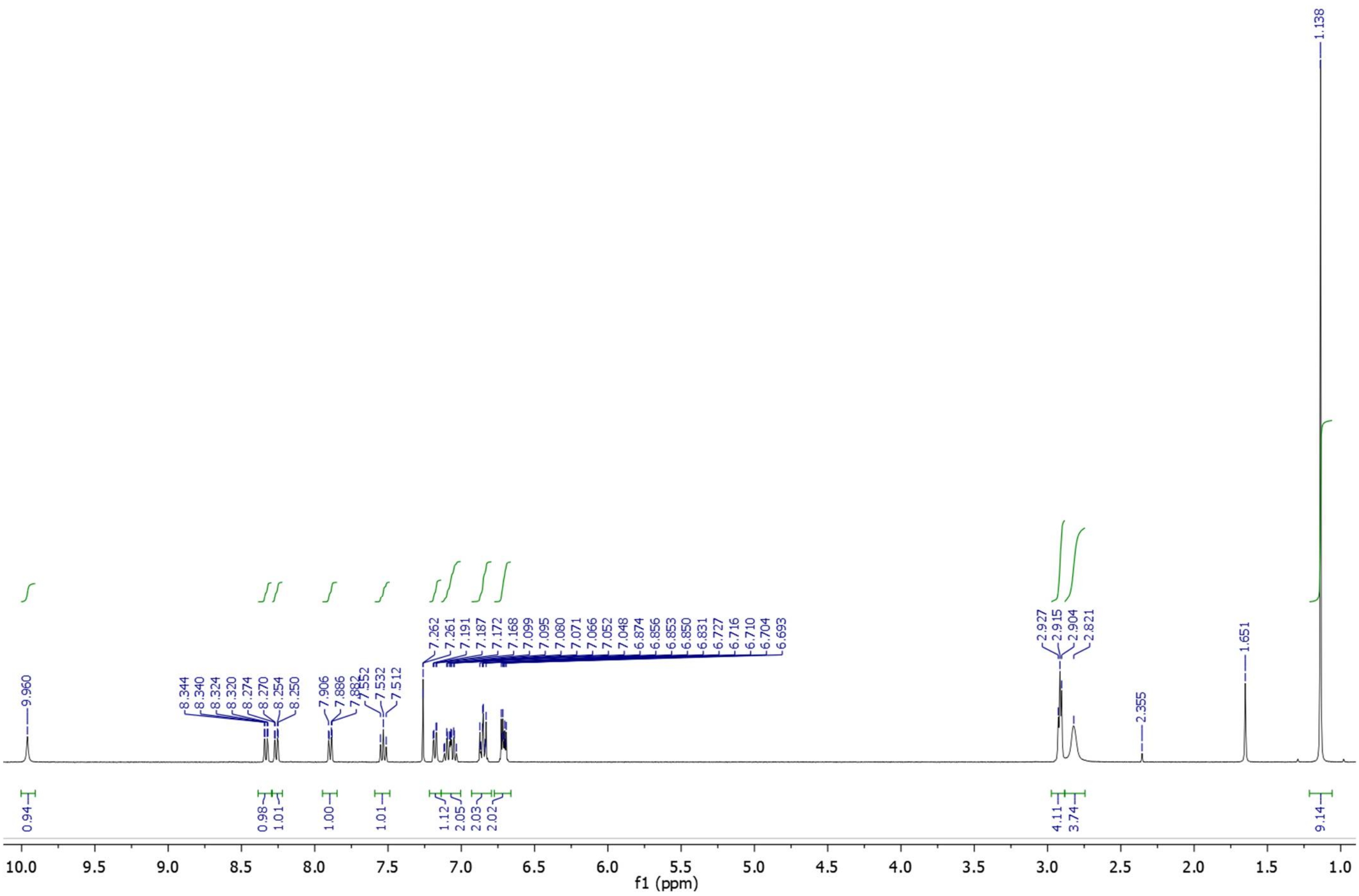

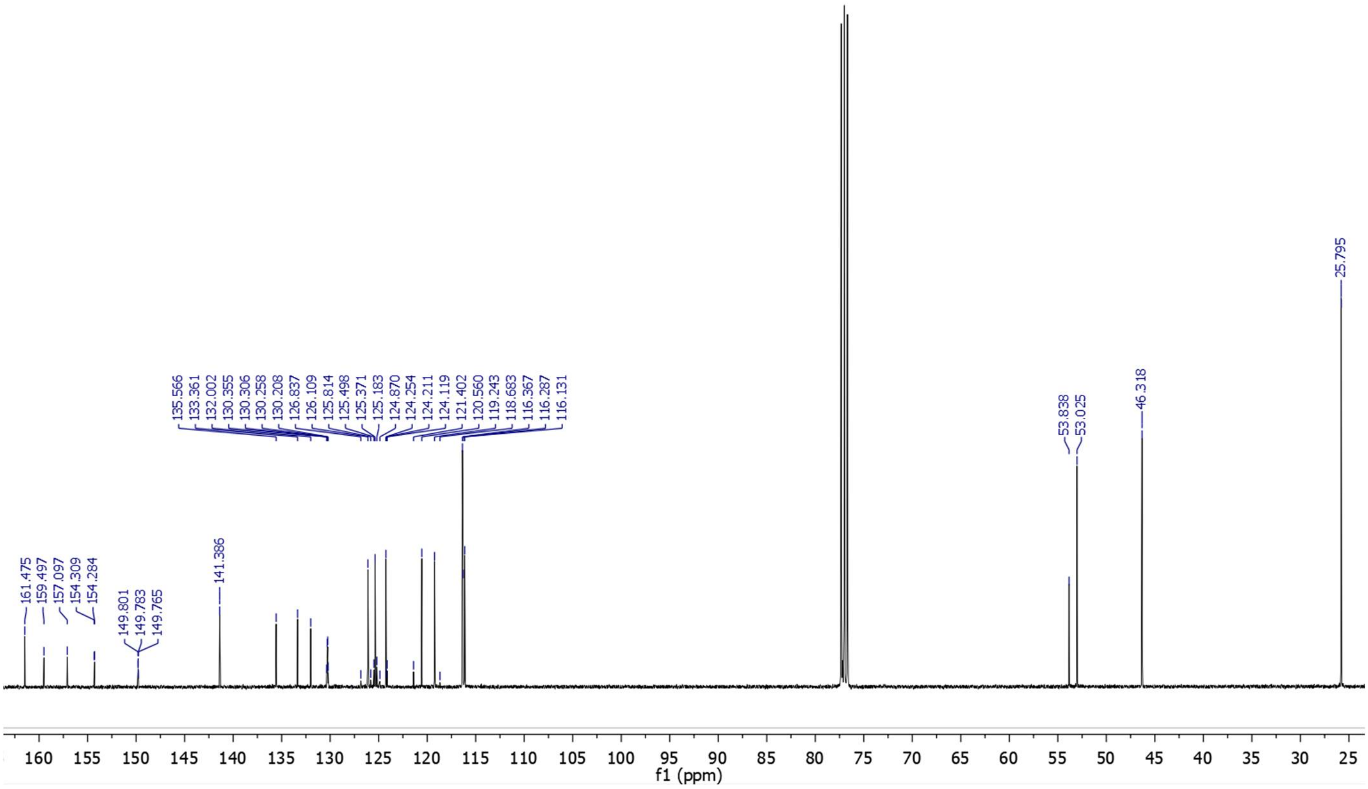

Figure S15. 1H NMR at 400 MHz and 13C NMR at 100 MHz spectra for compound **21**

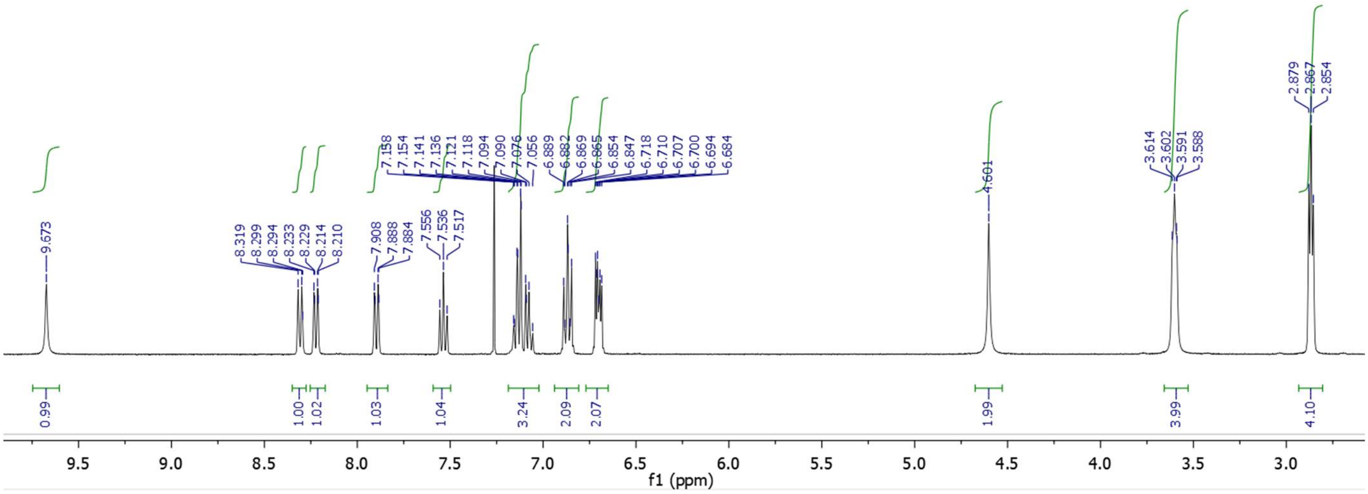

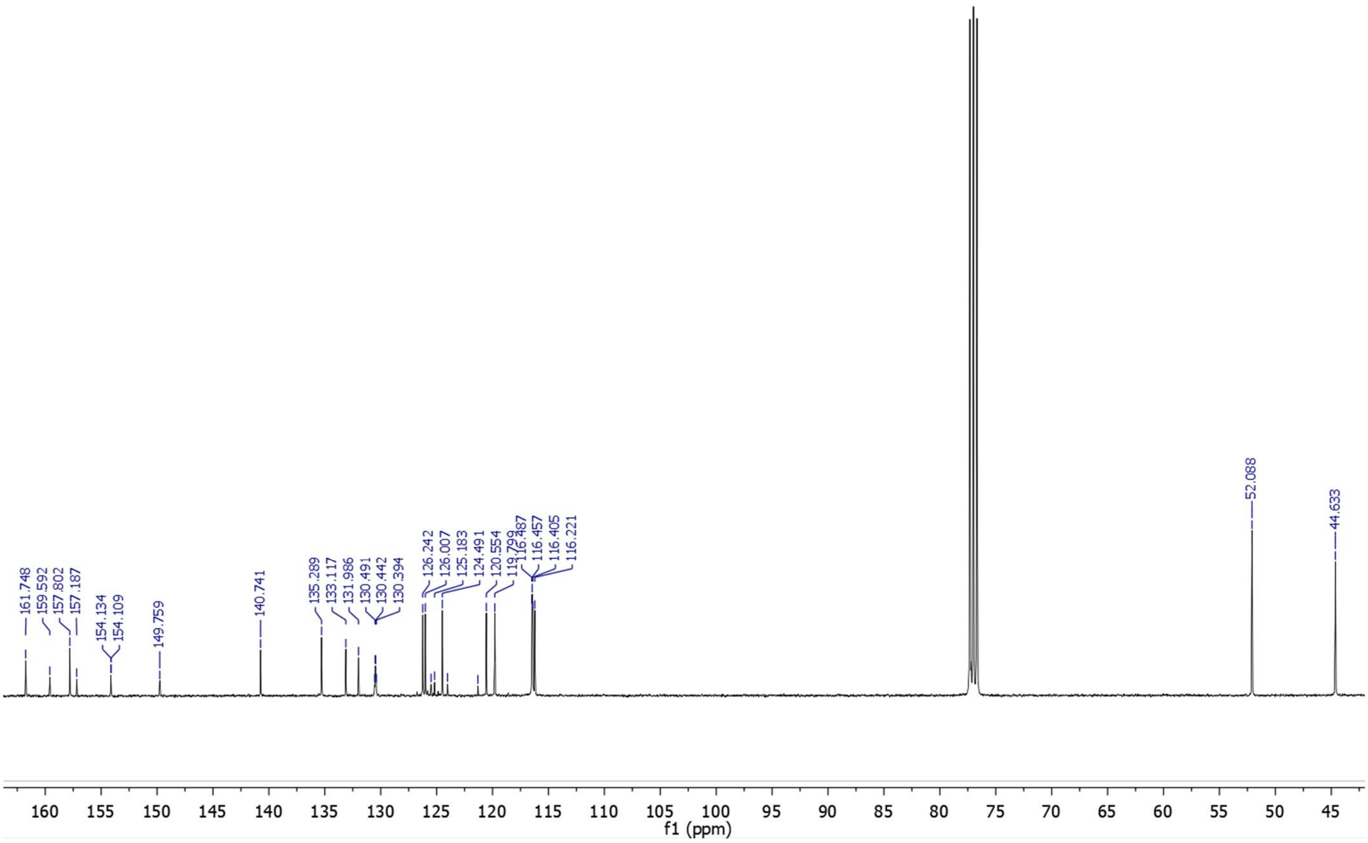

Figure S16. 1H NMR at 400 MHz and 13C NMR at 100 MHz spectra for compound **22**

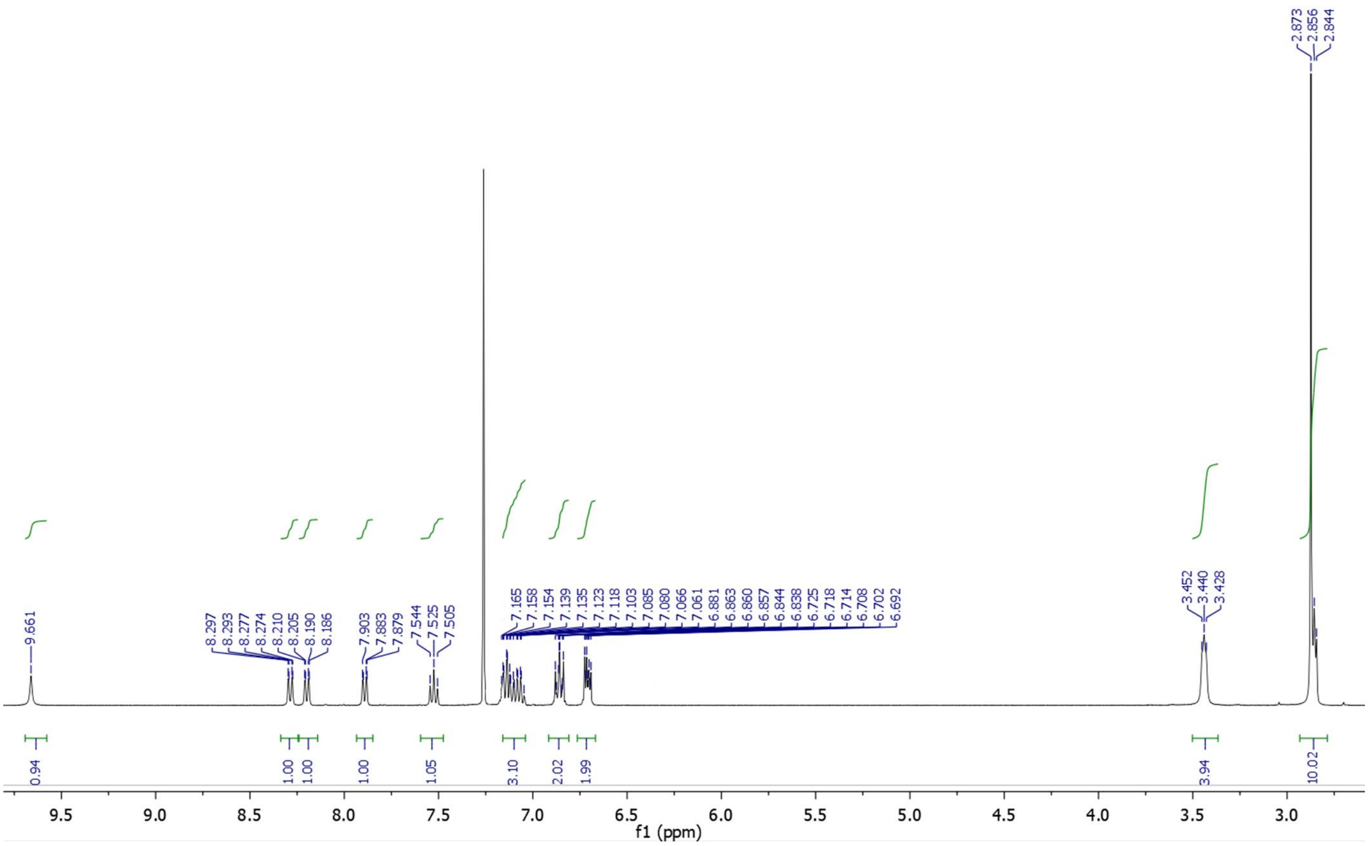

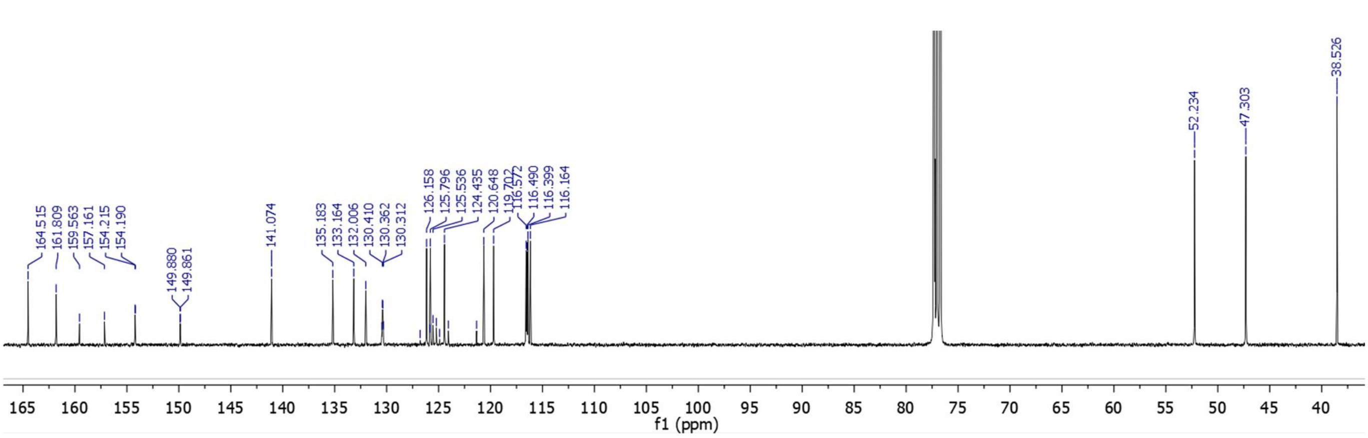

Figure S17. 1H NMR at 400 MHz and 13C NMR at 100 MHz spectra for compound **24**

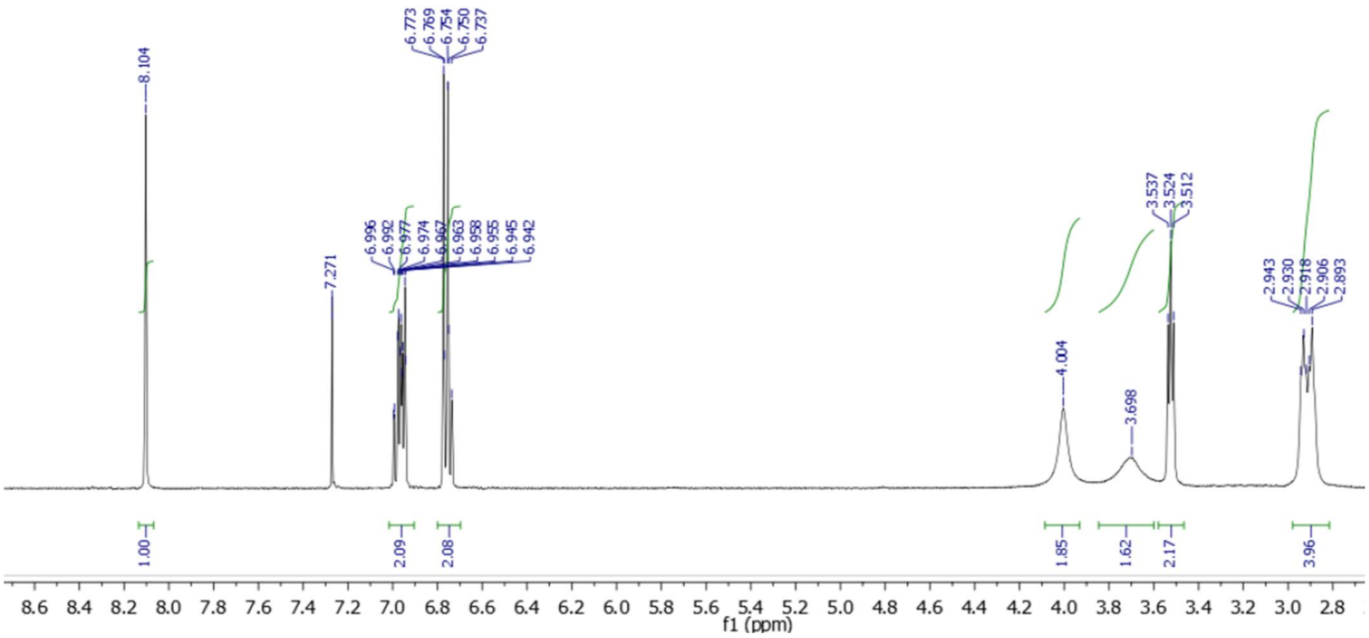

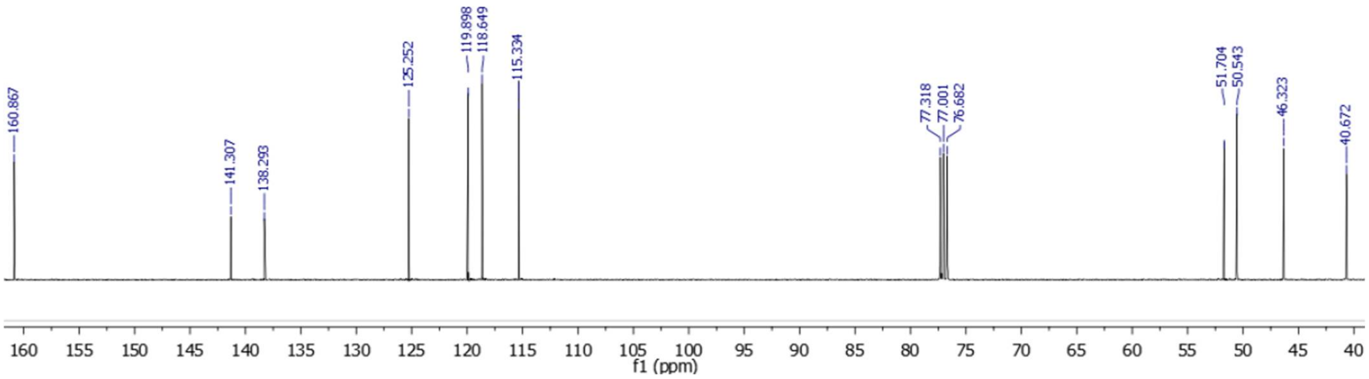

Figure S18. 1H NMR at 400 MHz and 13C NMR at 100 MHz spectra for compound **25**

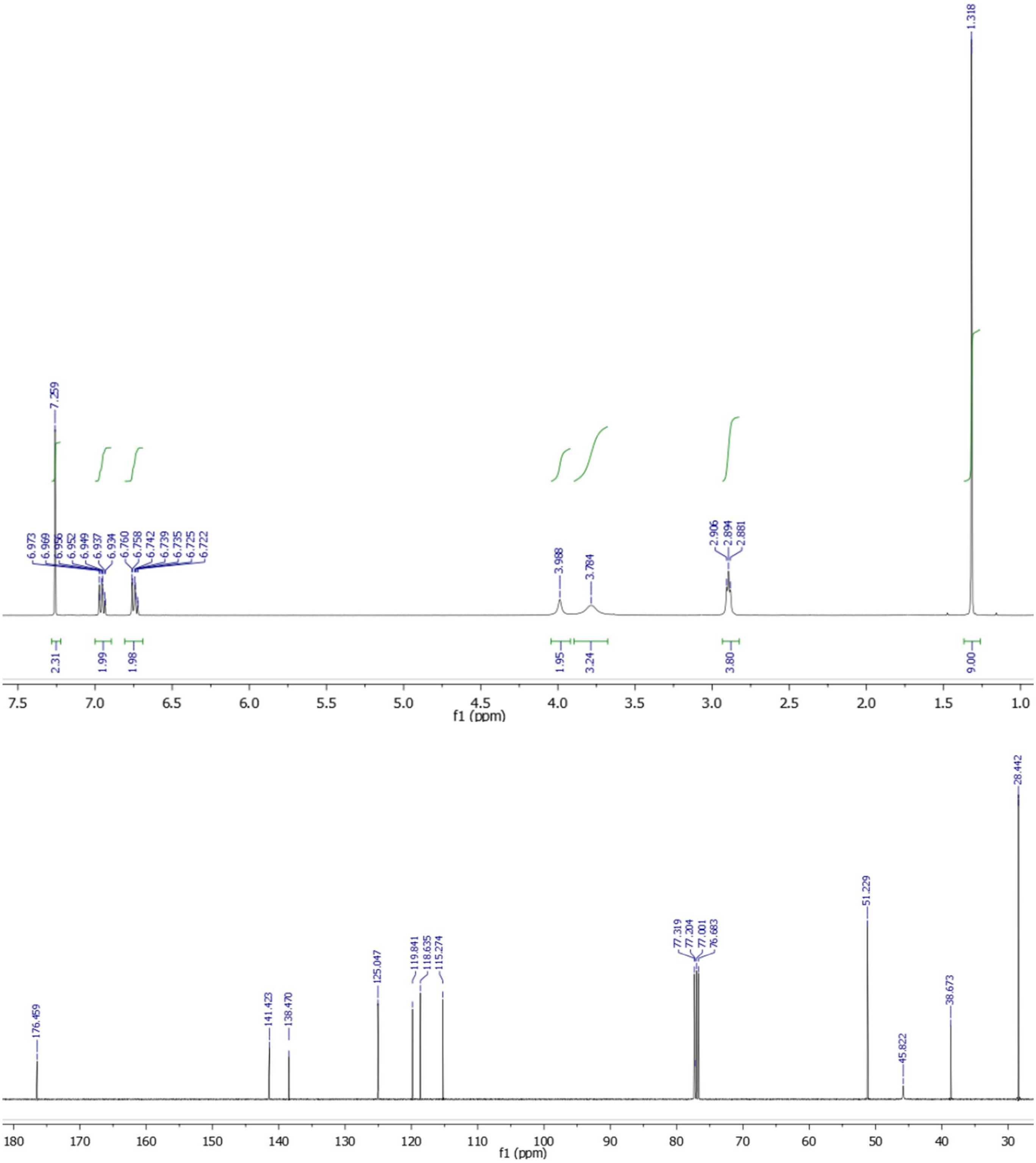

Figure S19. 1H NMR at 400 MHz and 13C NMR at 100 MHz spectra for compound **26**

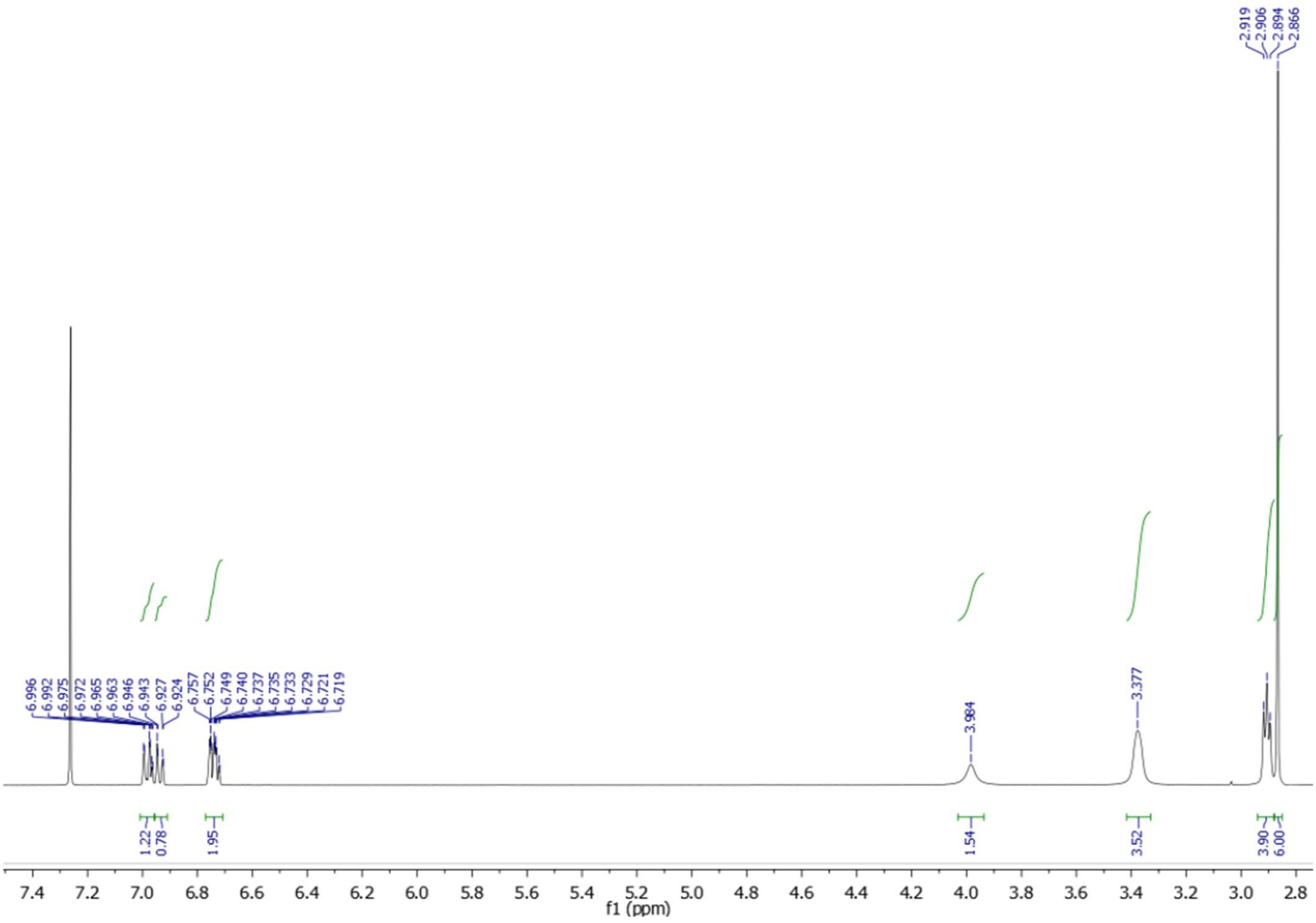

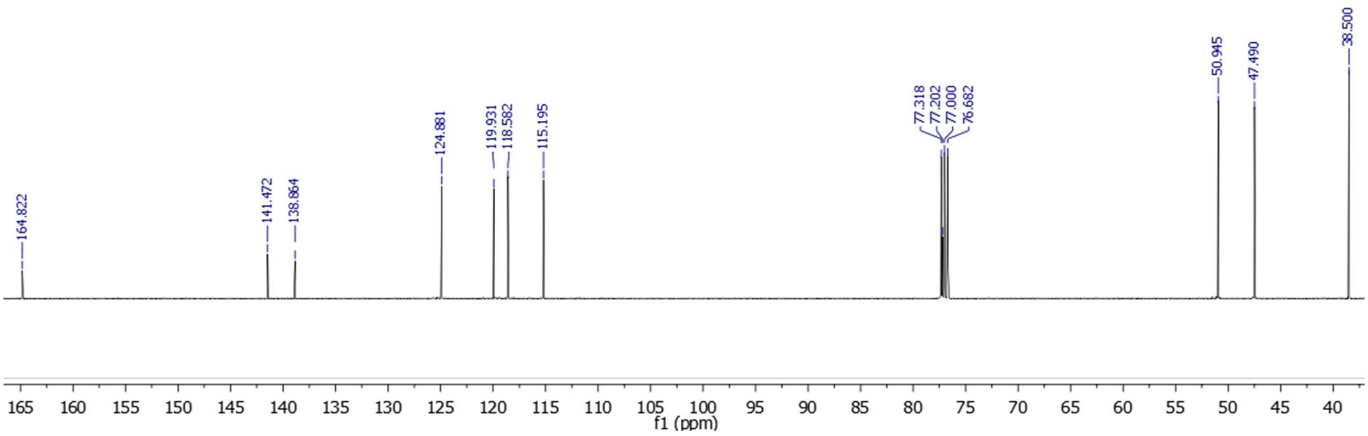

Figure S20. 1H NMR at 400 MHz and 13C NMR at 100 MHz spectra for compound **27**

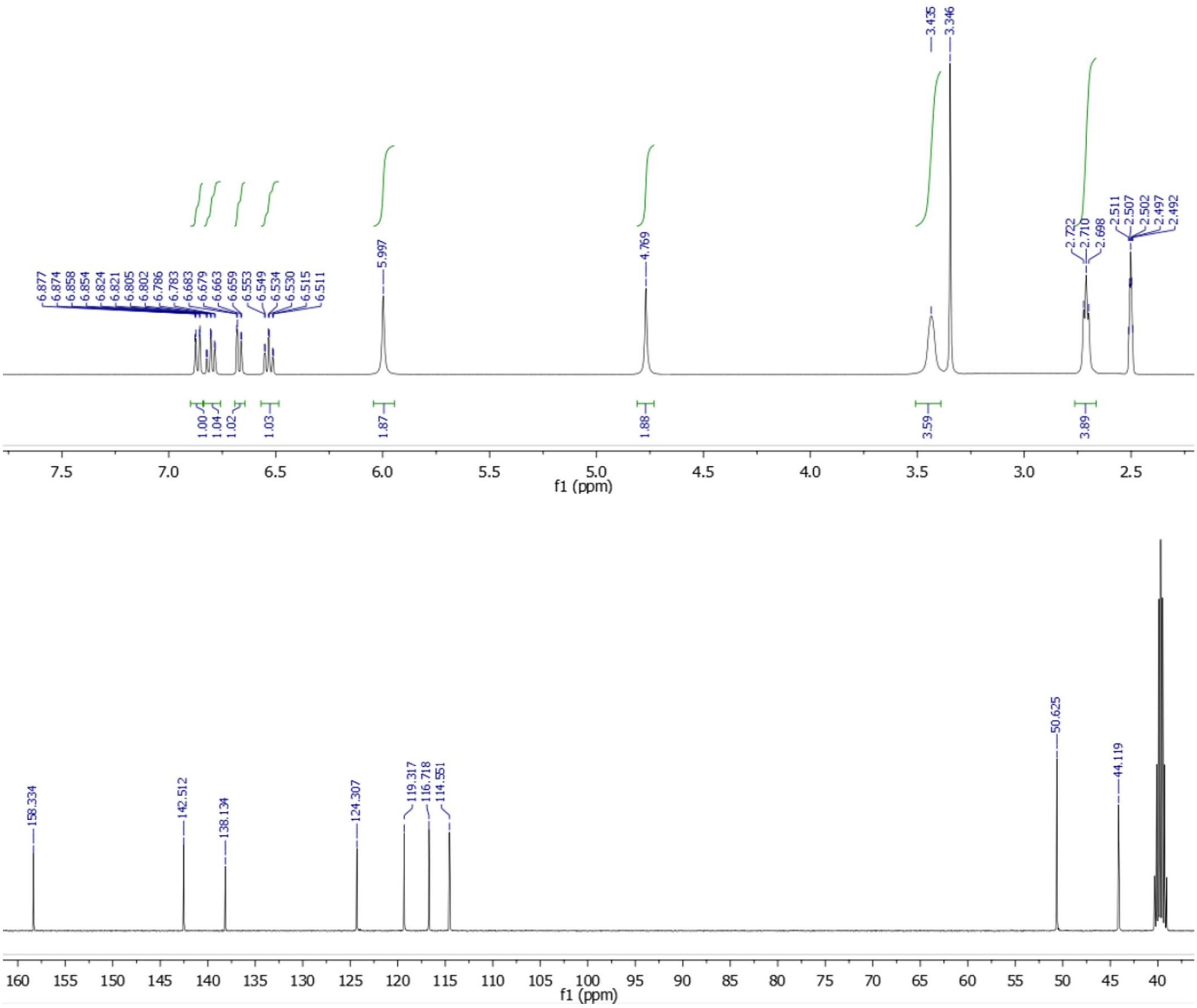

Figure S21. 1H NMR at 400 MHz and 13C NMR at 100 MHz spectra for compound **28**

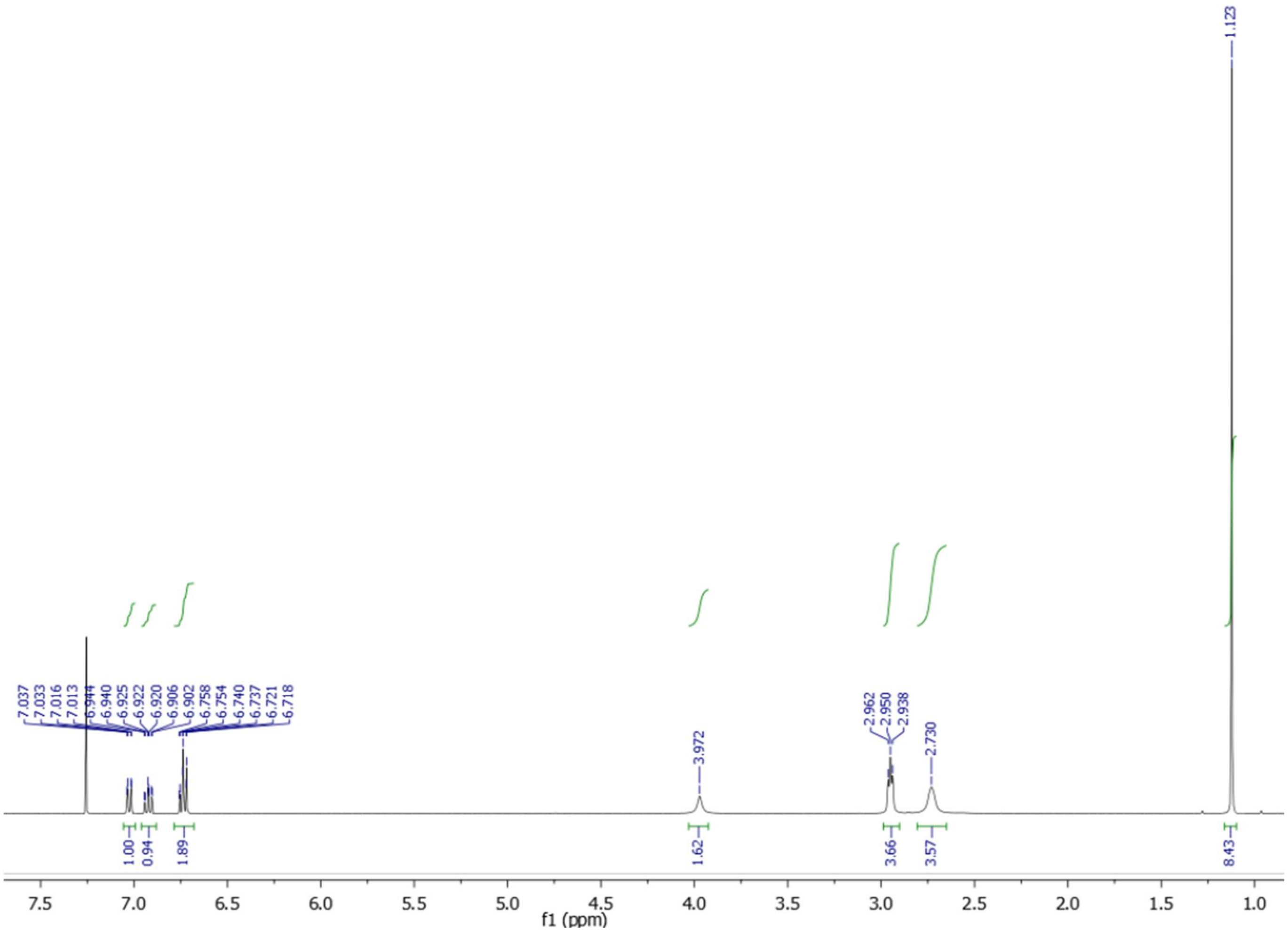

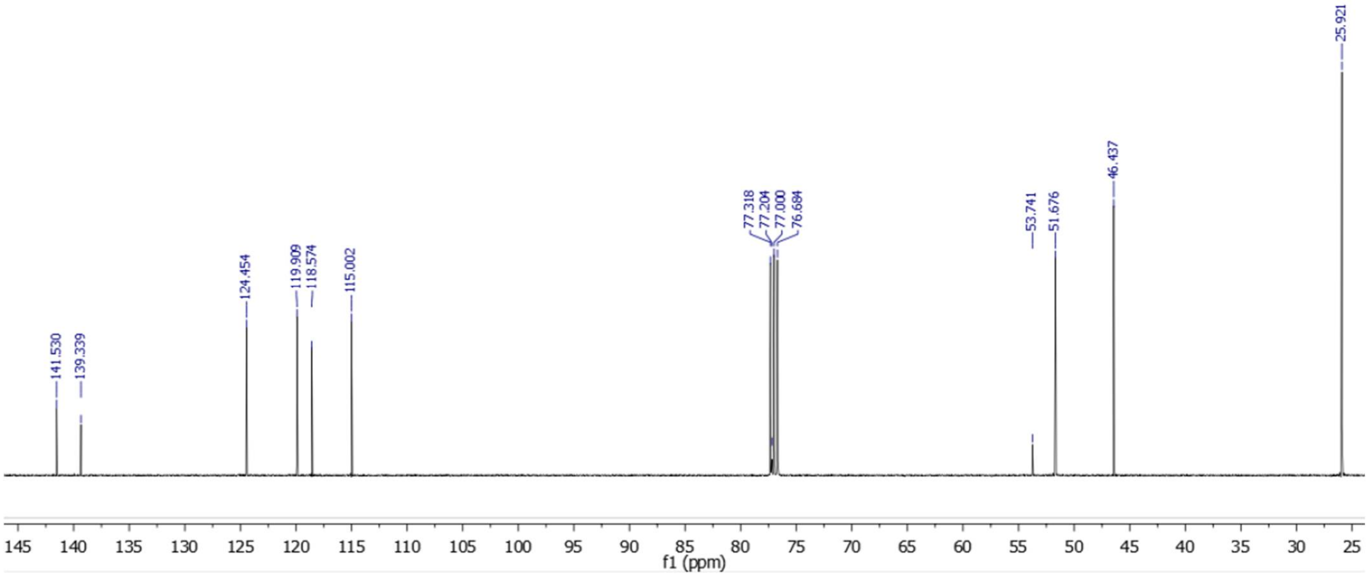

Figure S22. 1H NMR at 400 MHz and 13C NMR at 100 MHz spectra for compound **31**

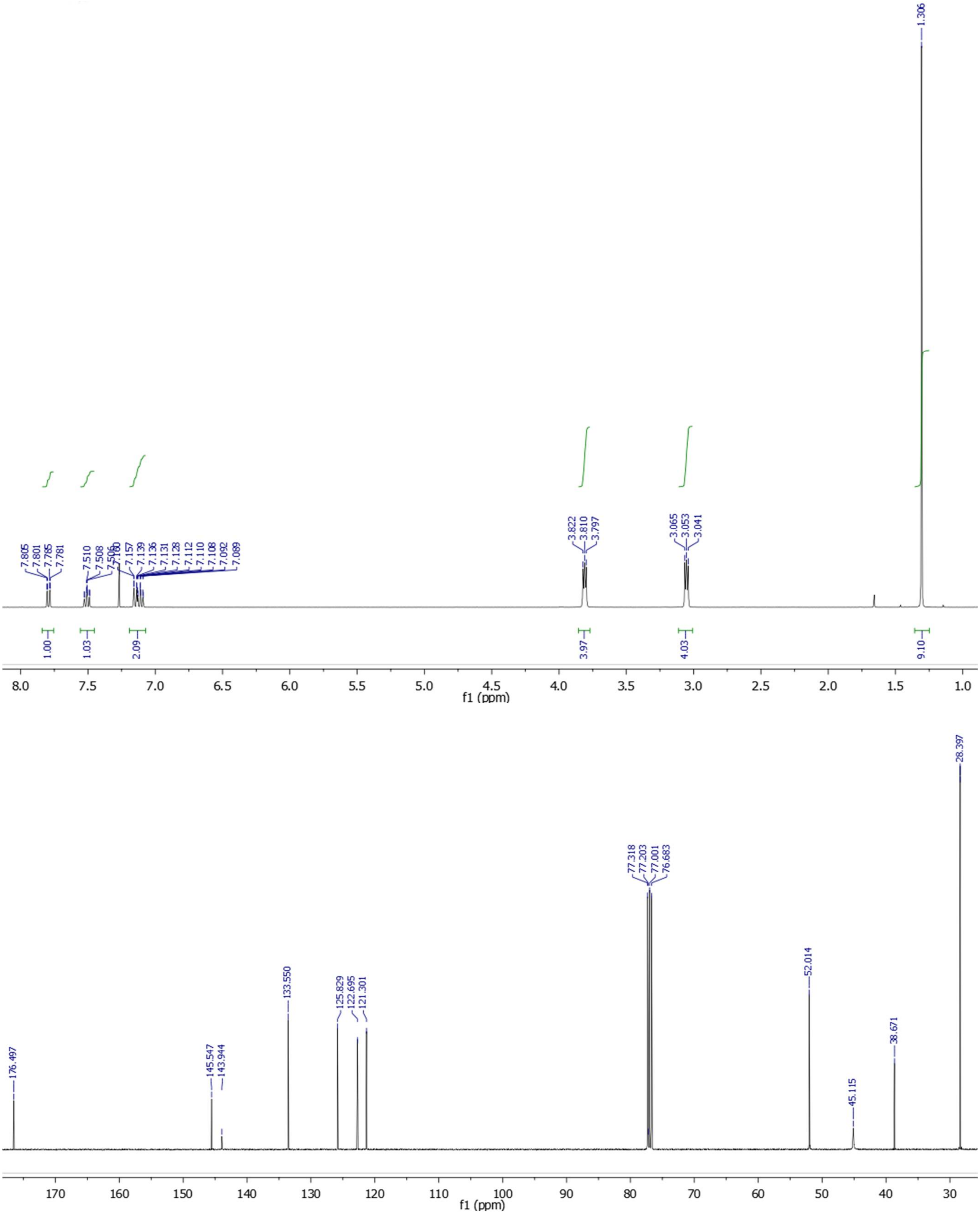

Figure S23. 1H NMR at 400 MHz and 13C NMR at 100 MHz spectra for compound **33**

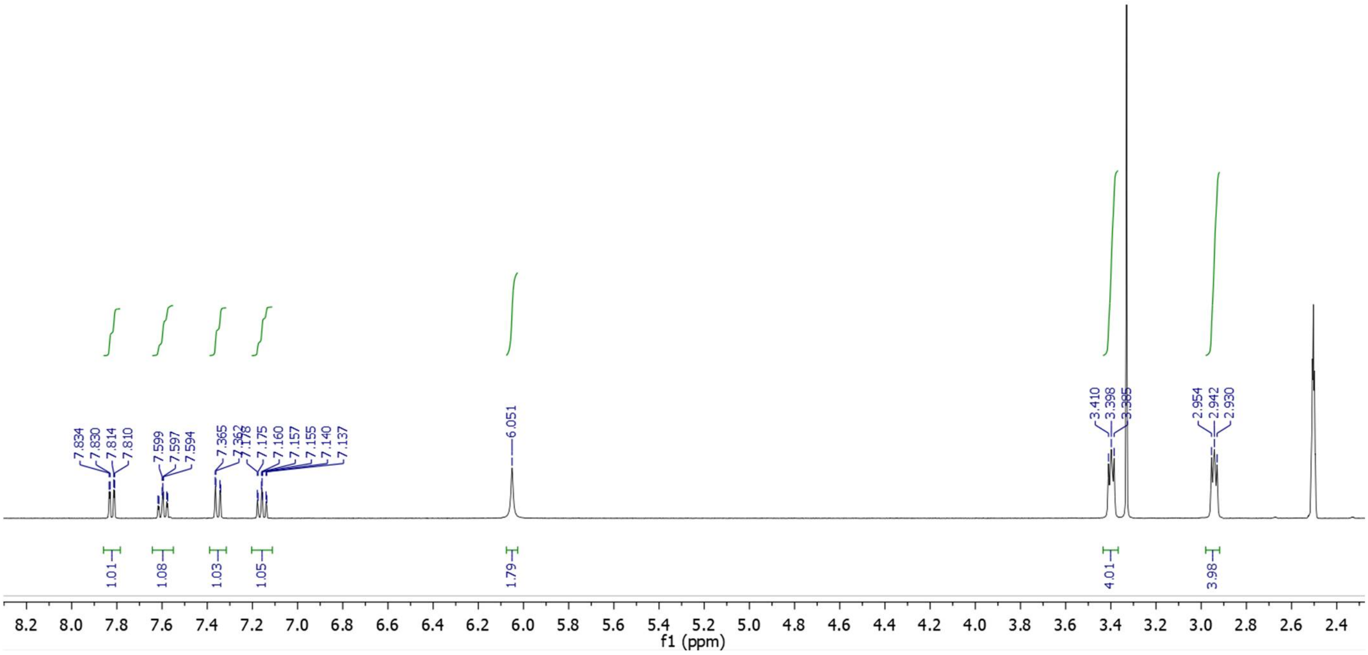

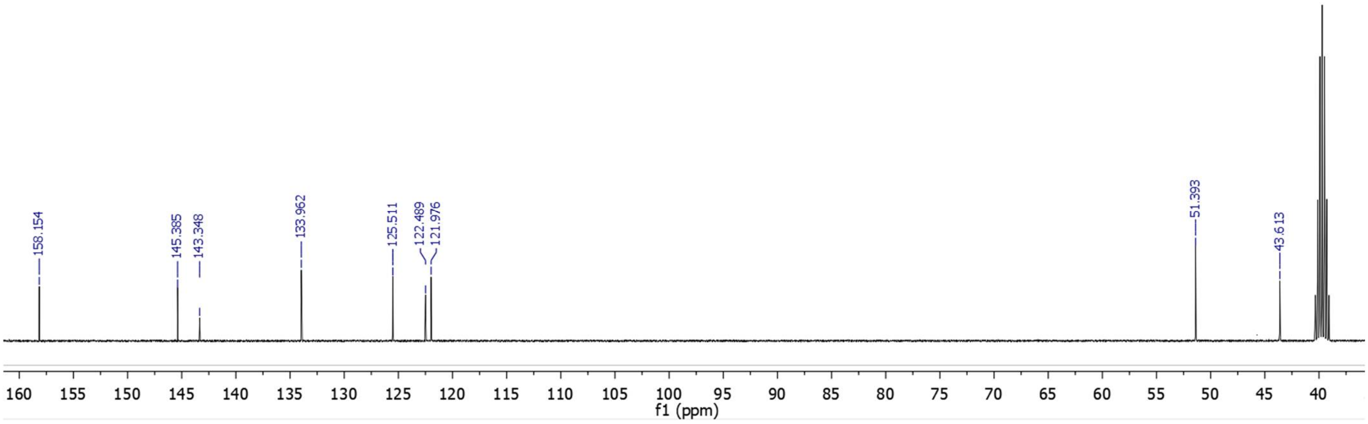

Figure S24. 1H NMR at 400 MHz and 13C NMR at 100 MHz spectra for compound **34**

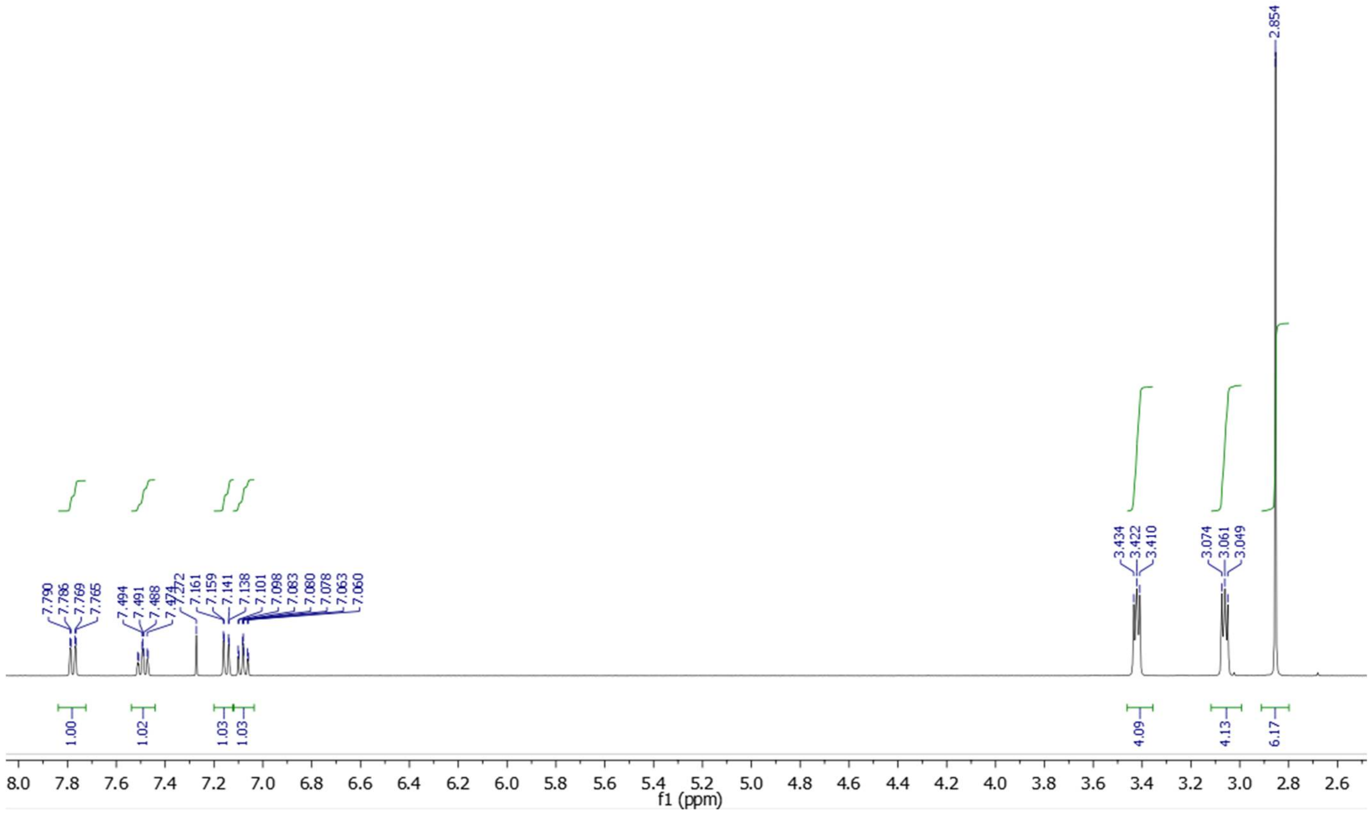

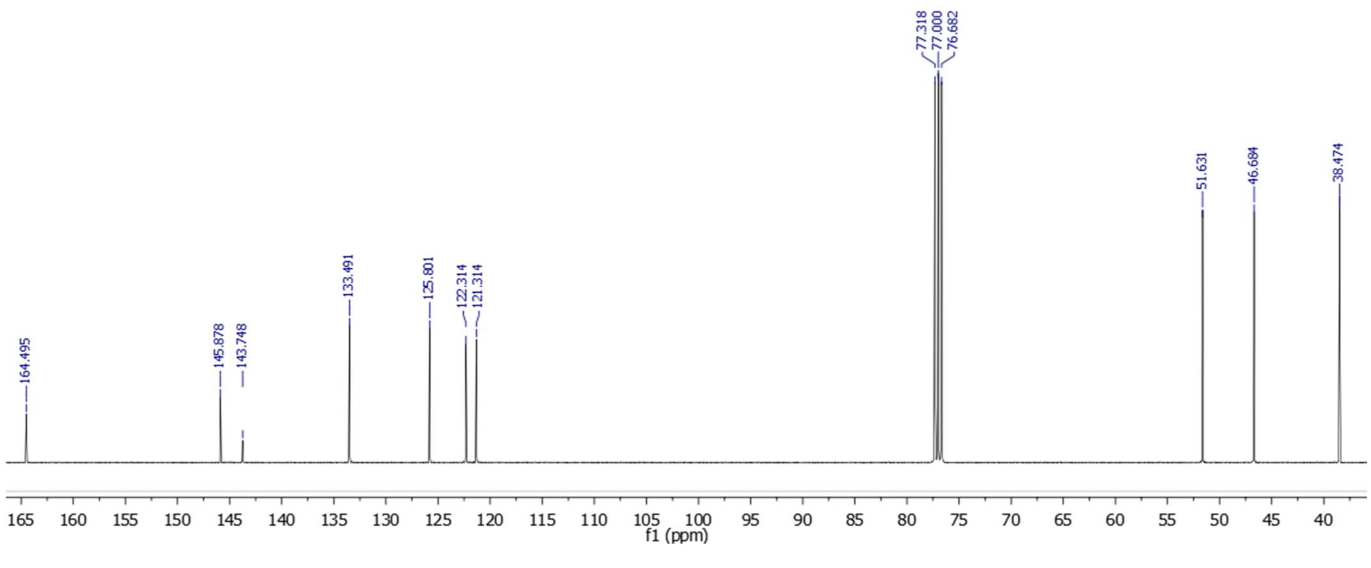

Figure S25. 1H NMR at 400 MHz and 13C NMR at 100 MHz spectra for compound **35**

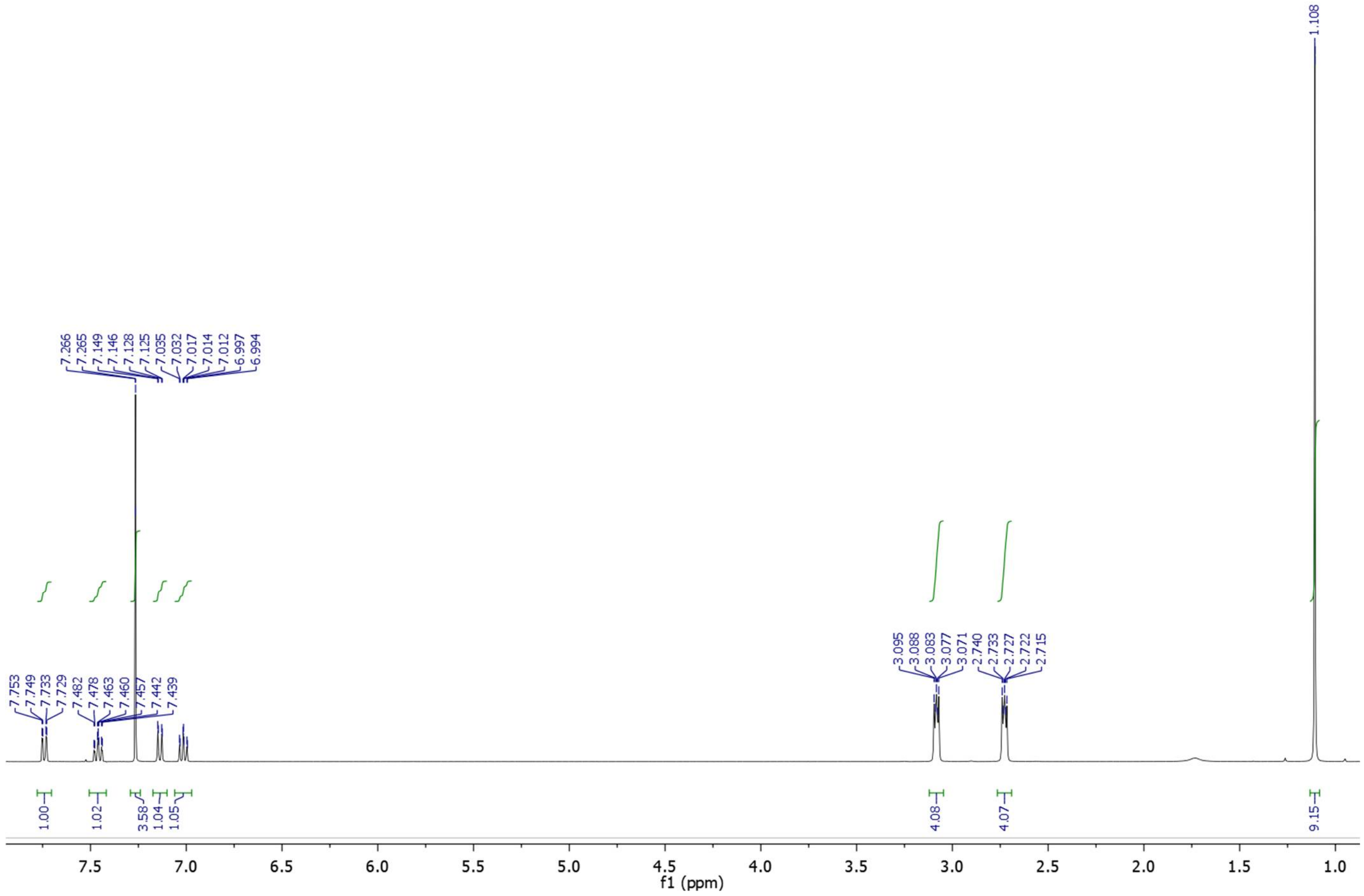

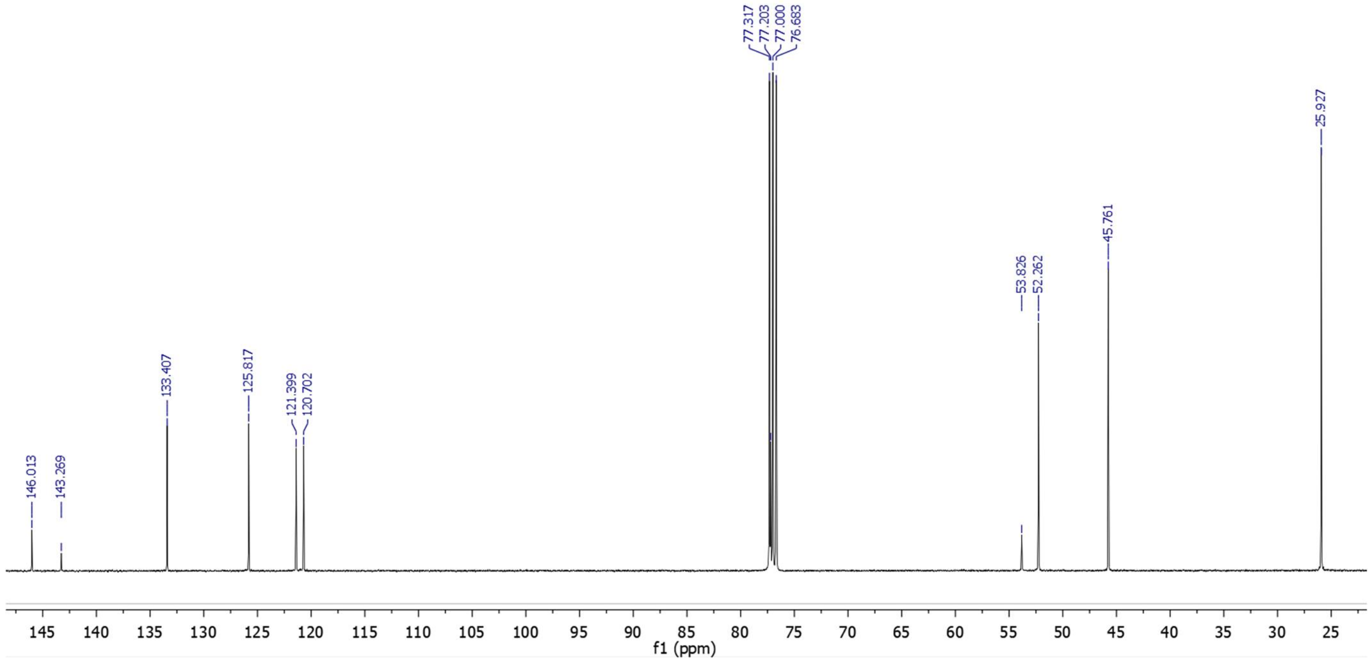

Figure S26. 1H NMR at 400 MHz and 13C NMR at 100 MHz spectra for compound **36**

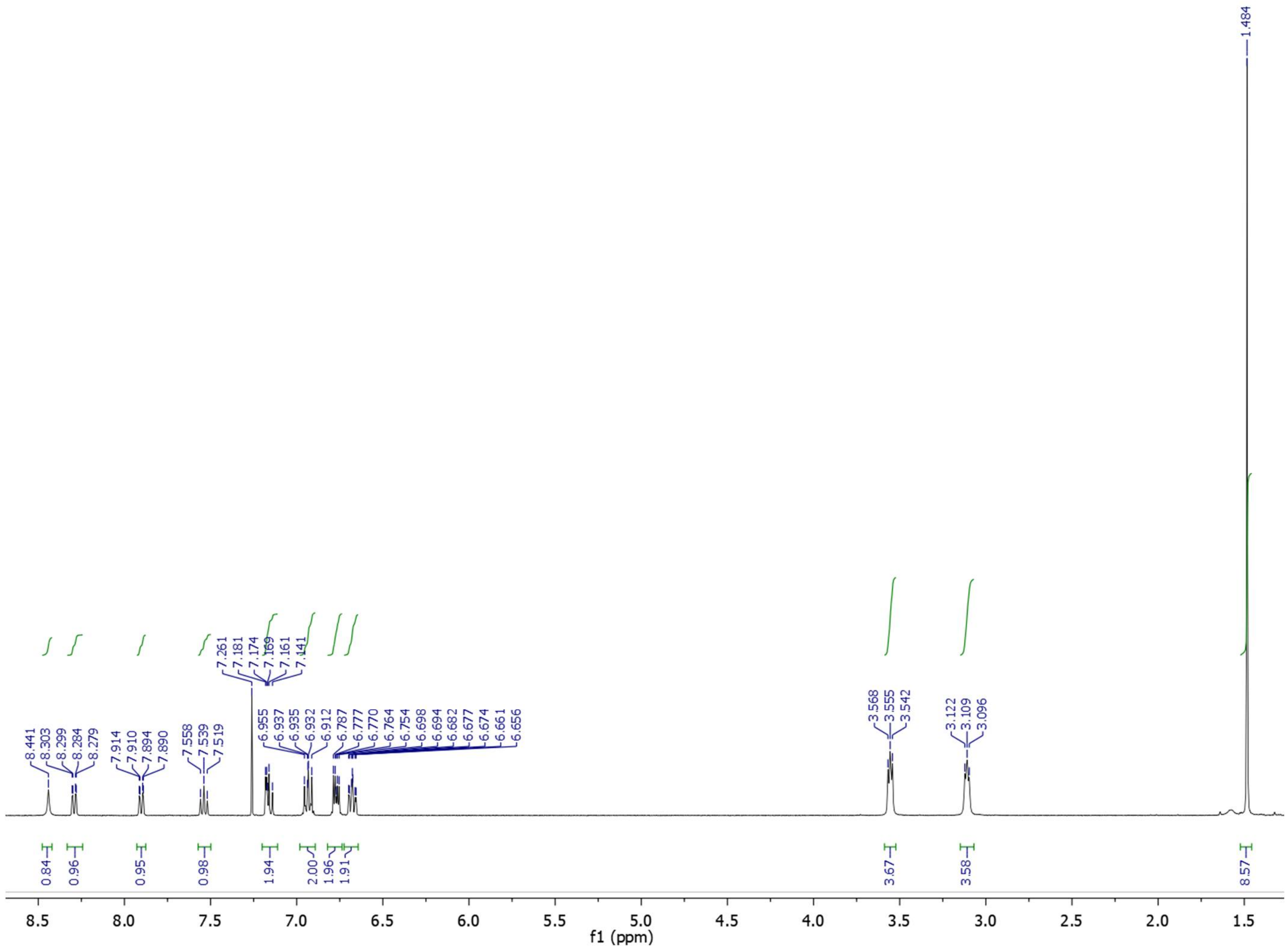

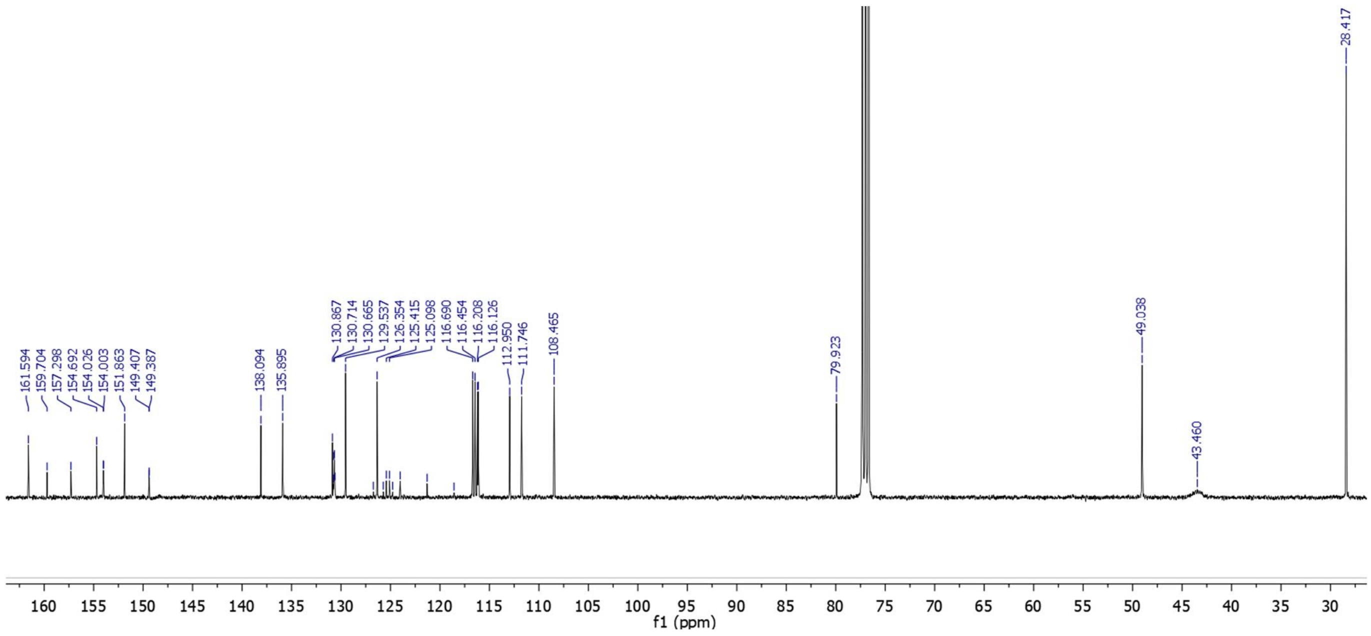

Figure S27. 1H NMR at 400 MHz and 13C NMR at 100 MHz spectra for compound **37**

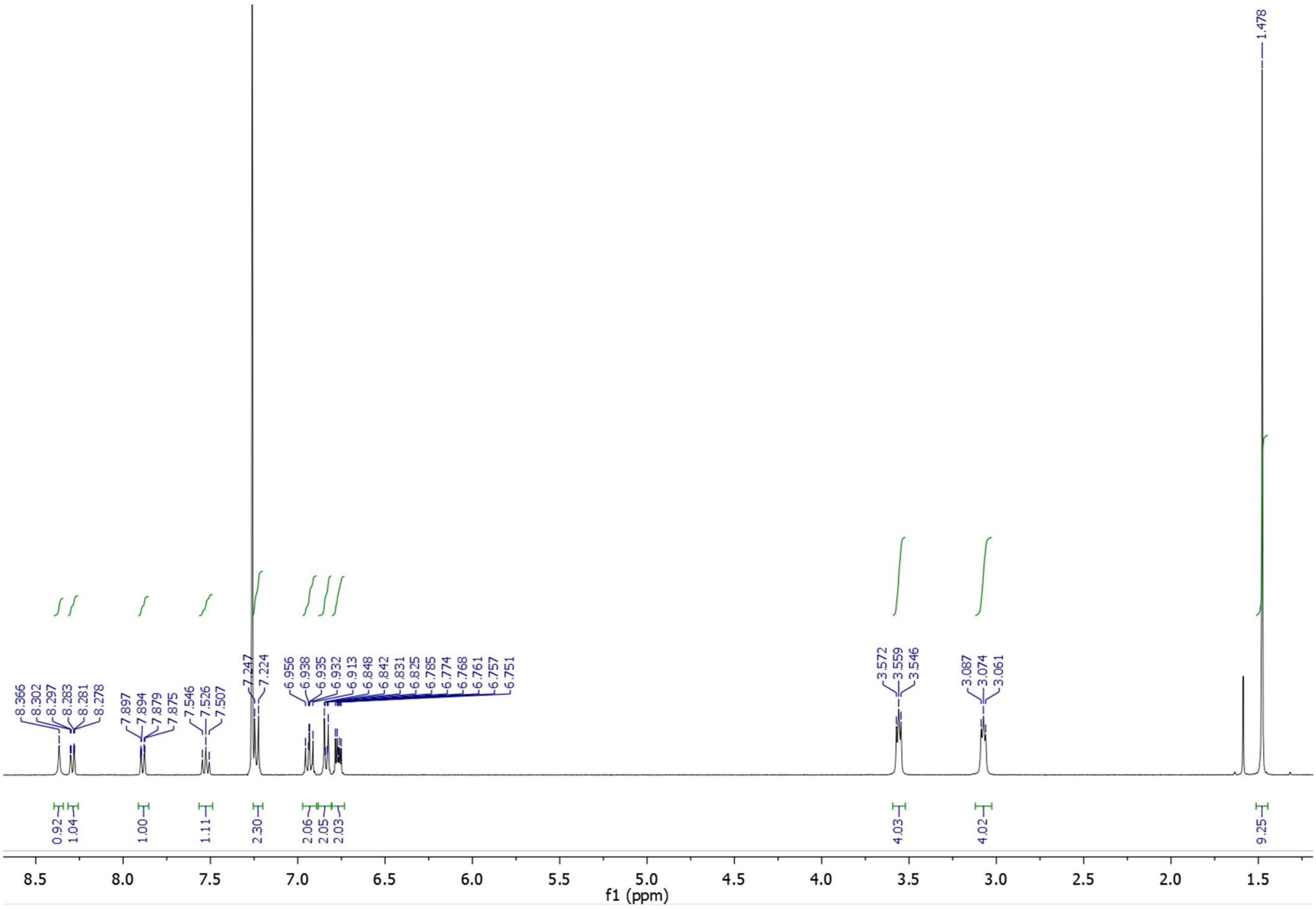

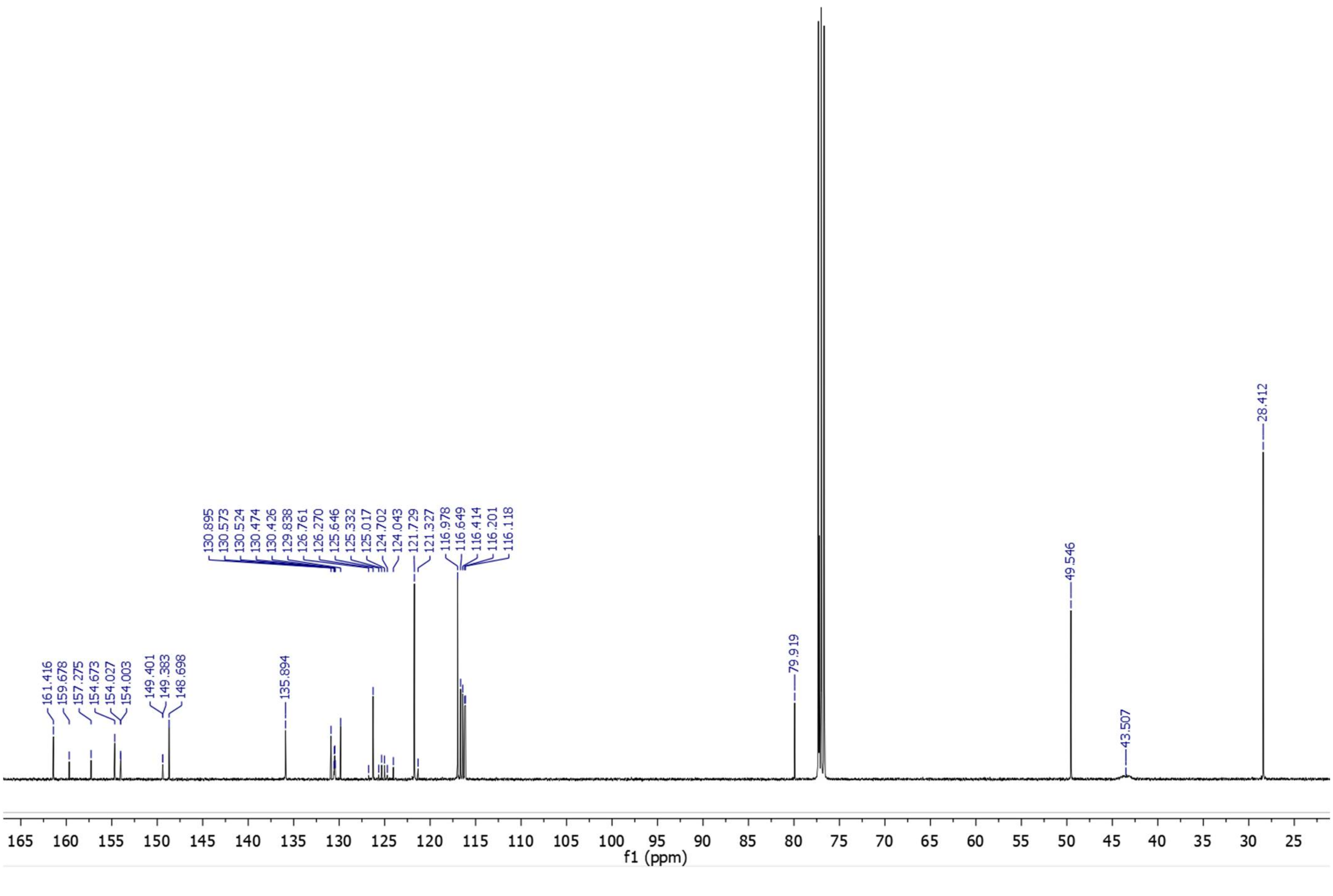

Figure S28. 1H NMR at 400 MHz and 13C NMR at 100 MHz spectra for compound **38**

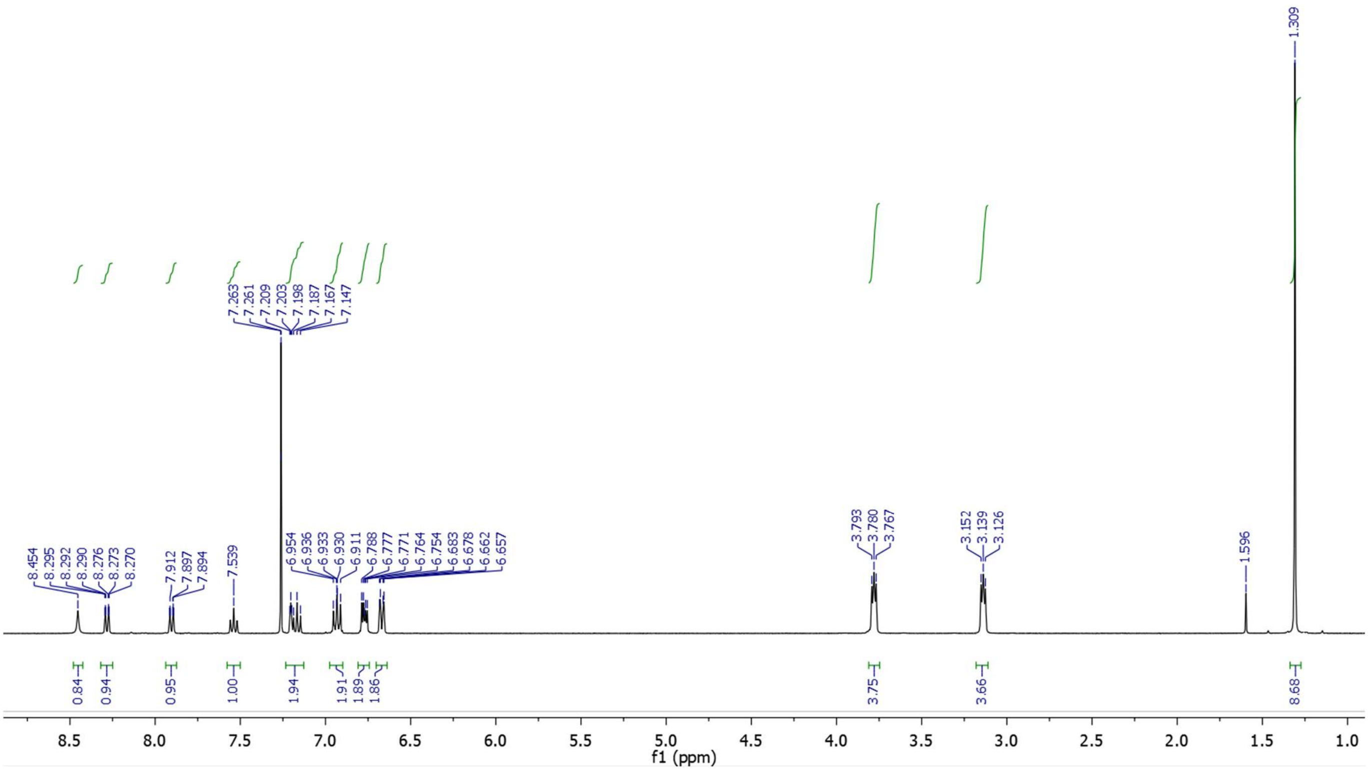

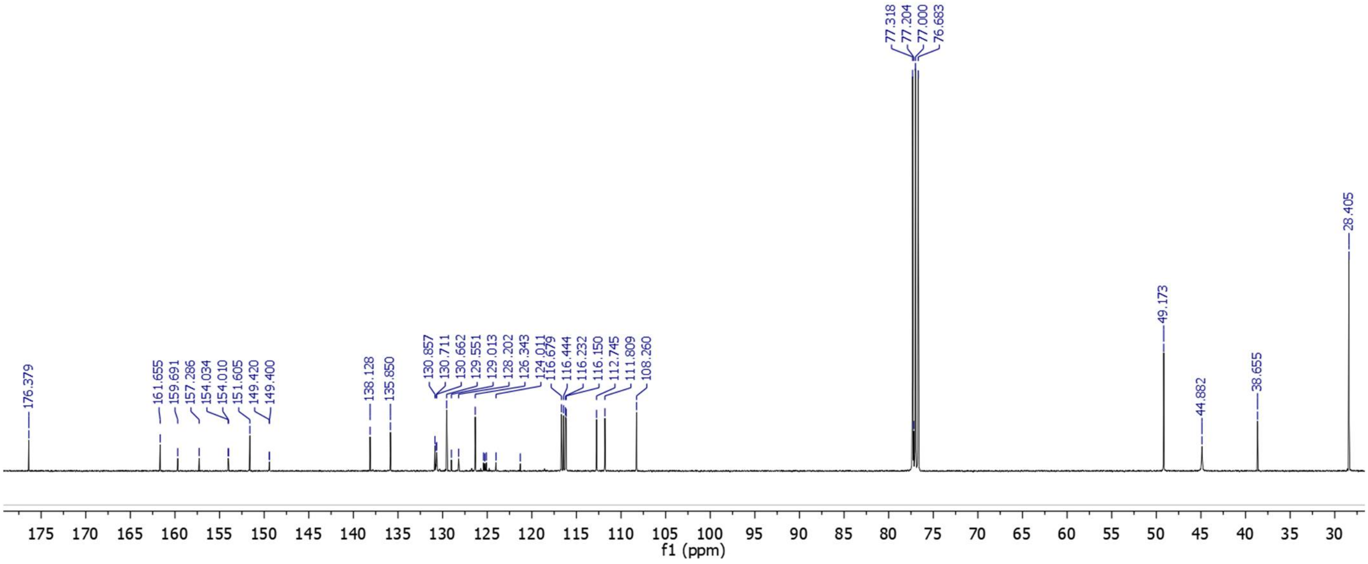

Figure S29. 1H NMR at 400 MHz and 13C NMR at 100 MHz spectra for compound **39**

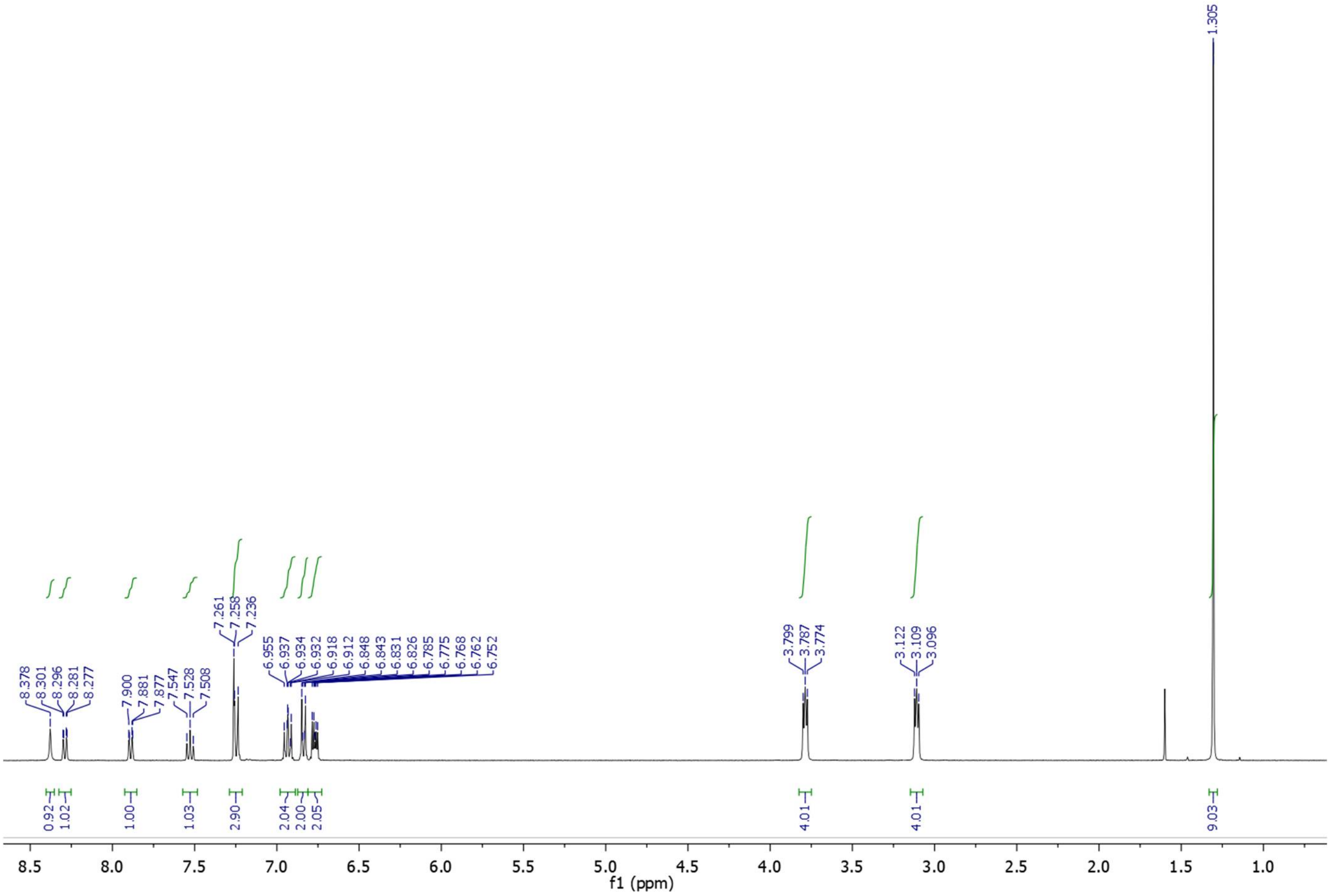

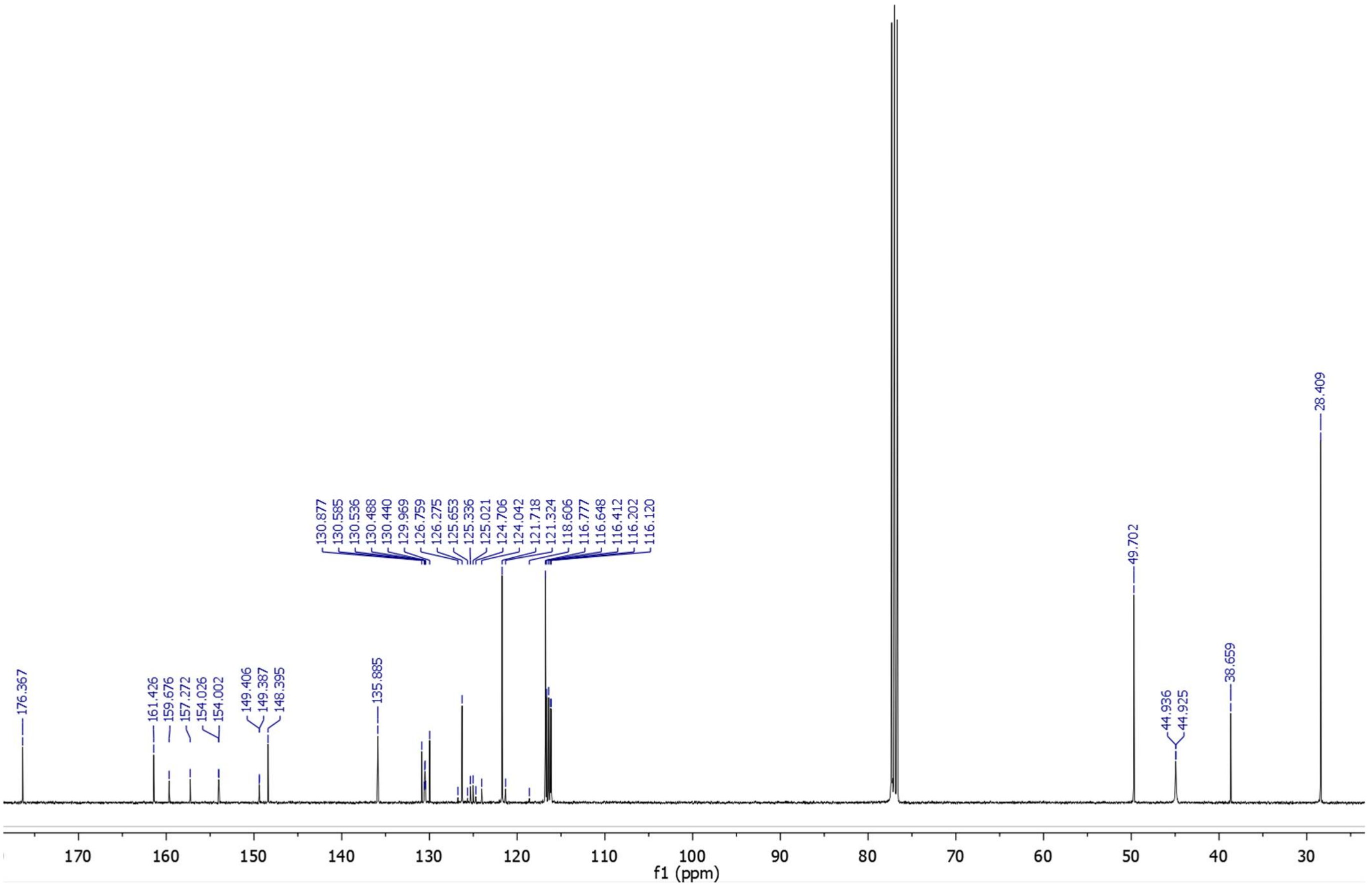

Figure S30. 1H NMR at 400 MHz and 13C NMR at 100 MHz spectra for compound **42**

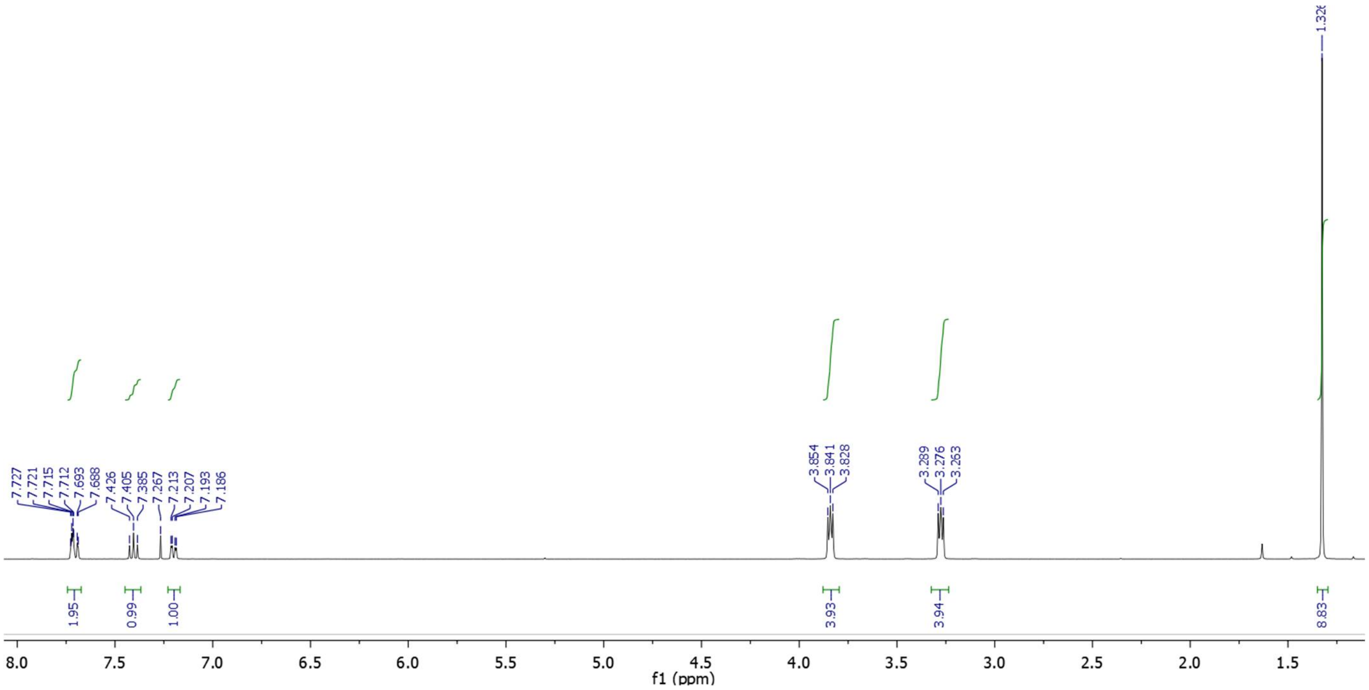

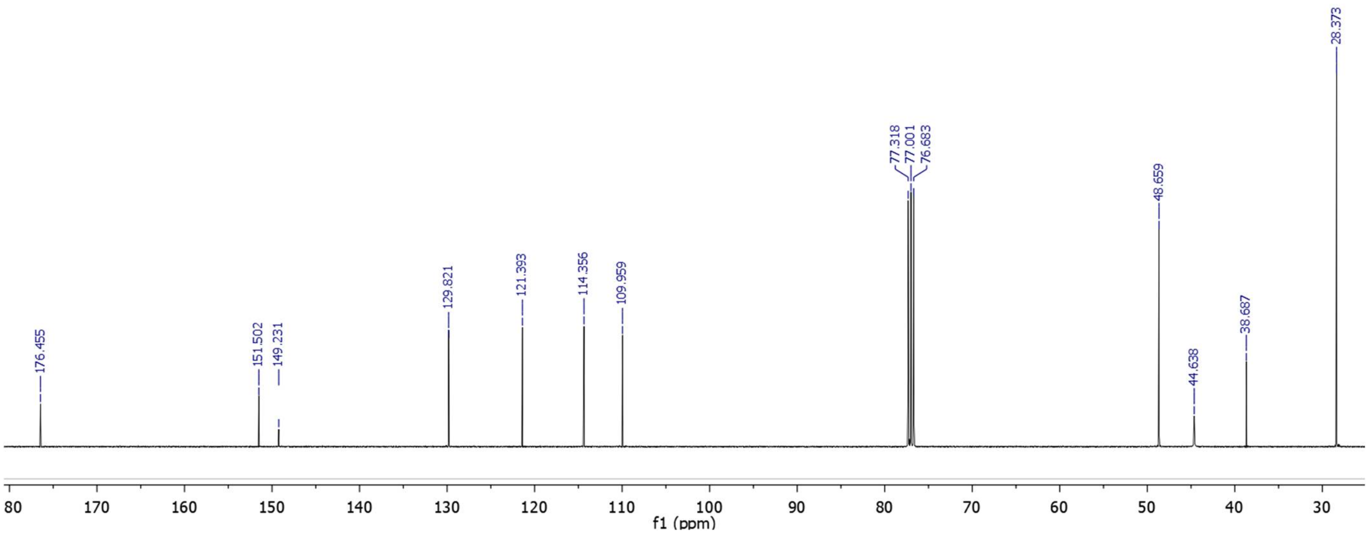

Figure S31. 1H NMR at 400 MHz and 13C NMR at 100 MHz spectra for compound **43**

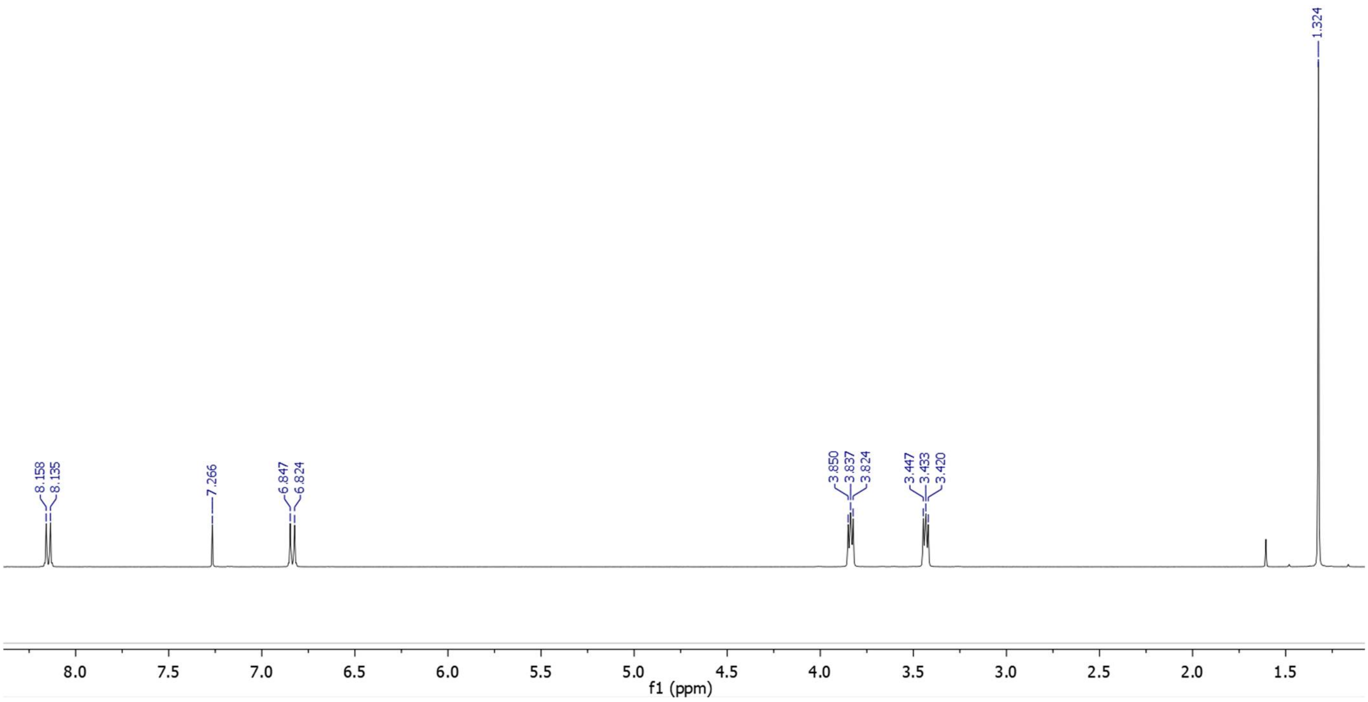

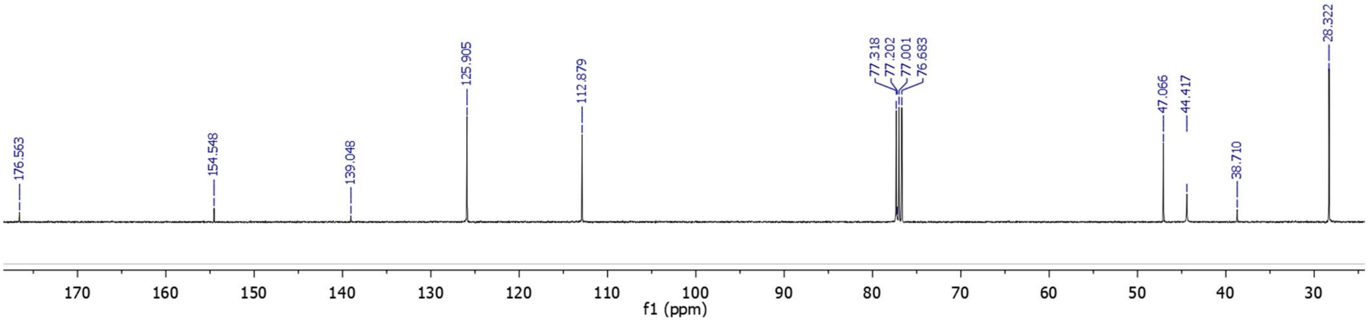

Figure S32. 1H NMR at 400 MHz and 13C NMR at 100 MHz spectra for compound **47**

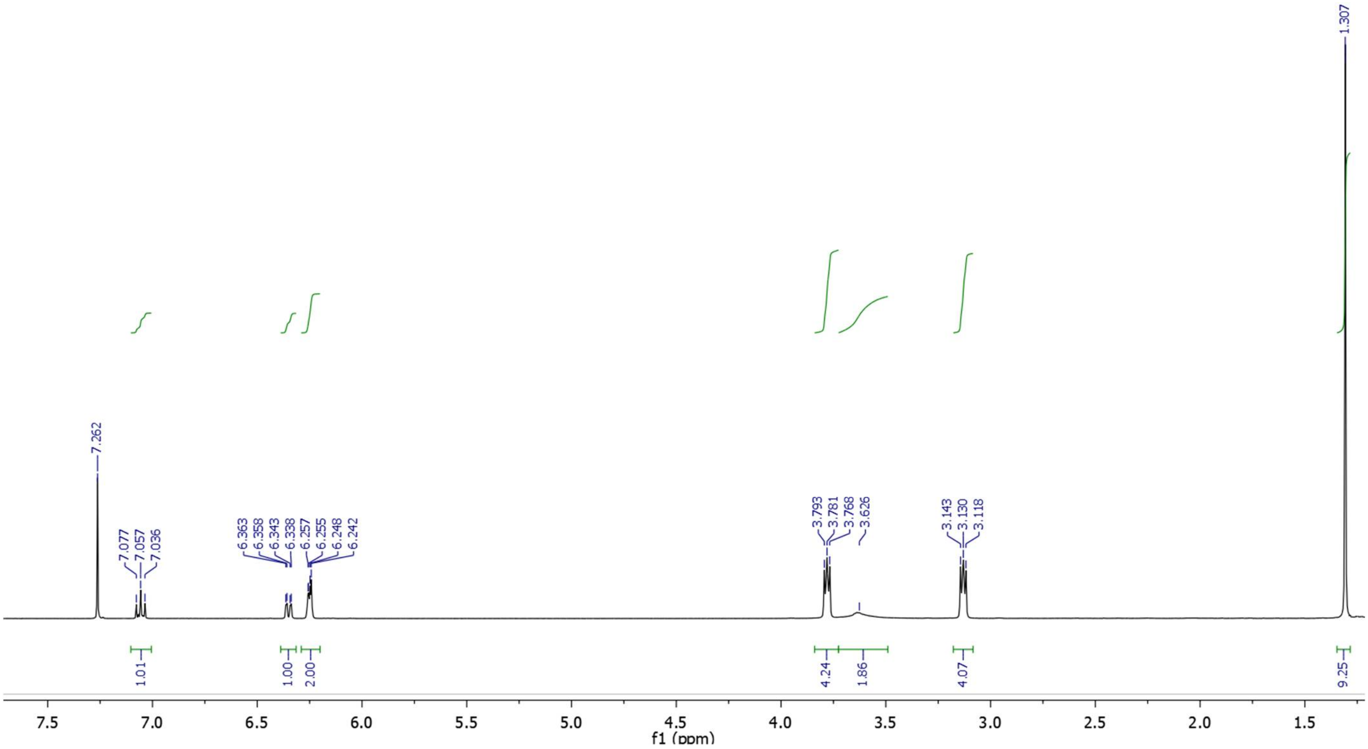

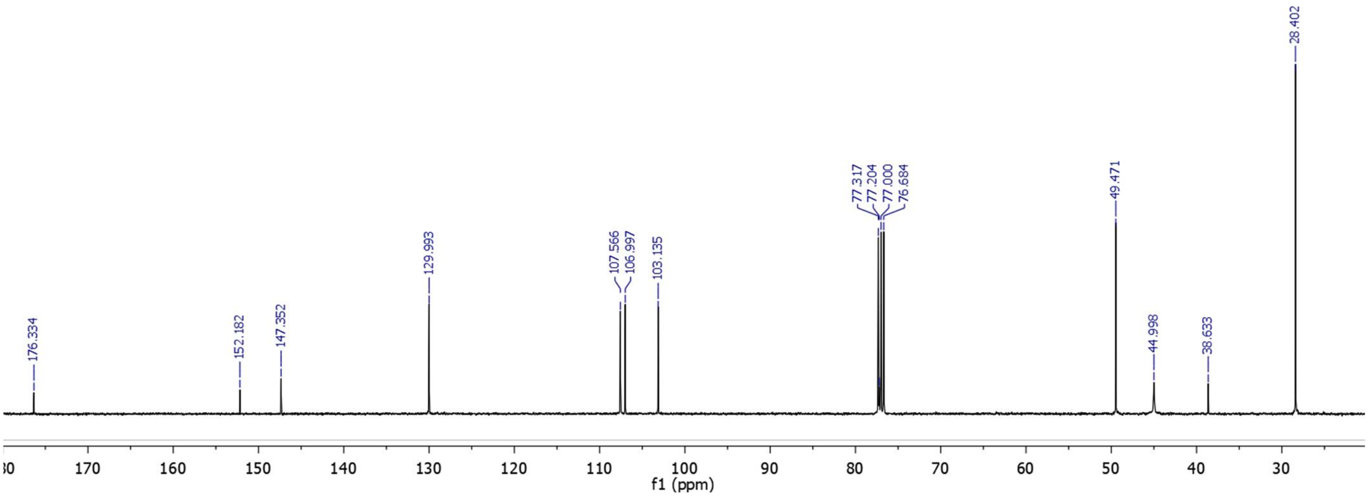

Figure S33. 1H NMR at 400 MHz and 13C NMR at 100 MHz spectra for compound **49**

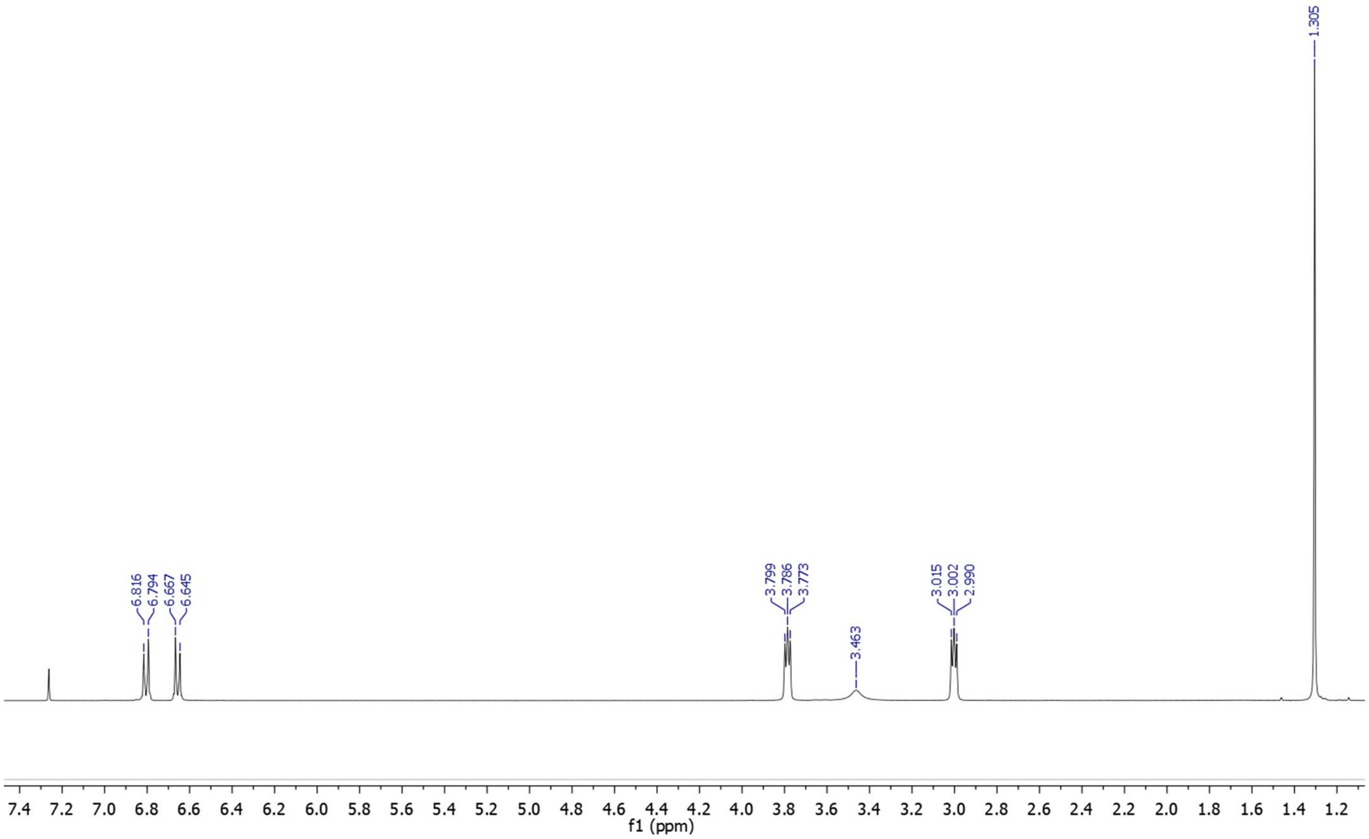

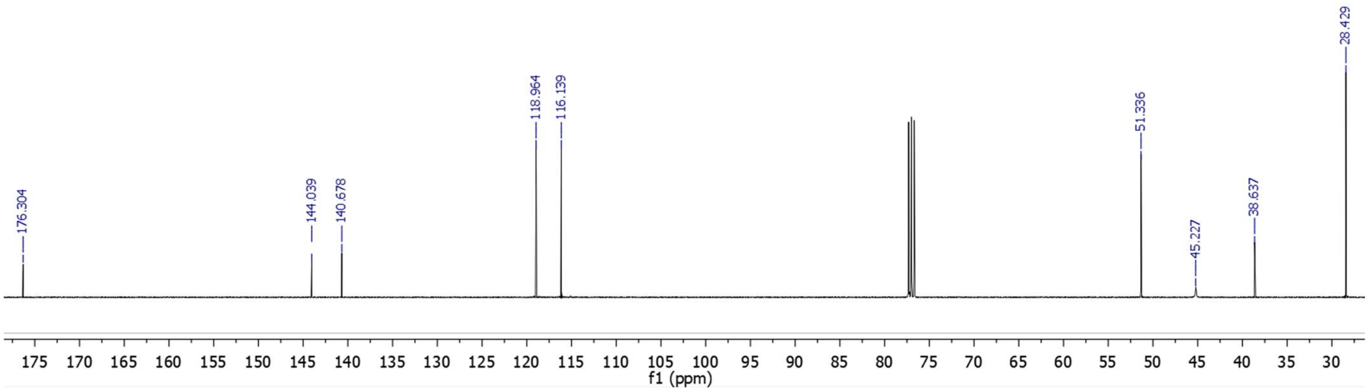

Figure S34. 1H NMR at 400 MHz and 13C NMR at 100 MHz spectra for compound **50**

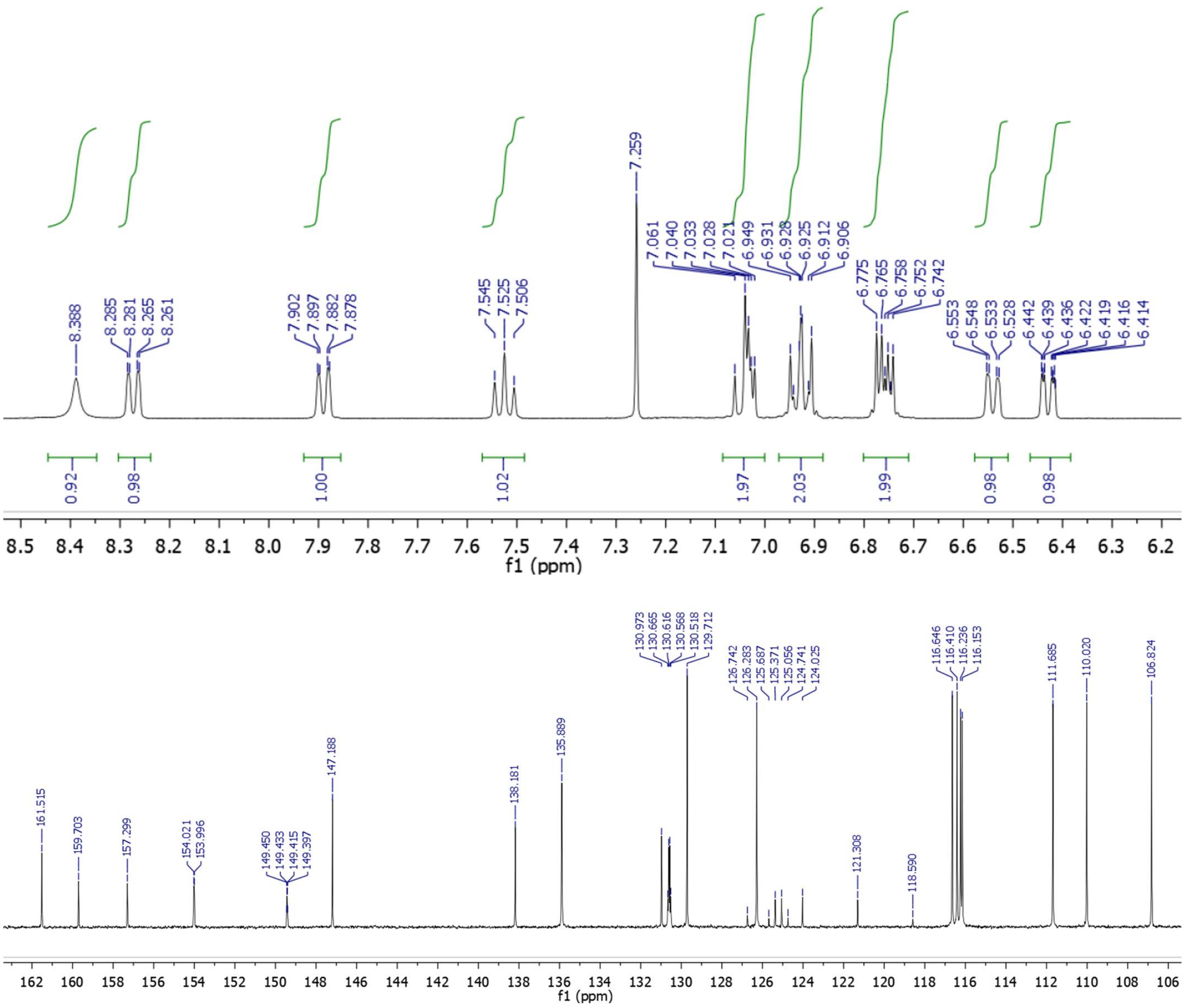

Figure S35. 1H NMR at 400 MHz and 13C NMR at 100 MHz spectra for compound **51**

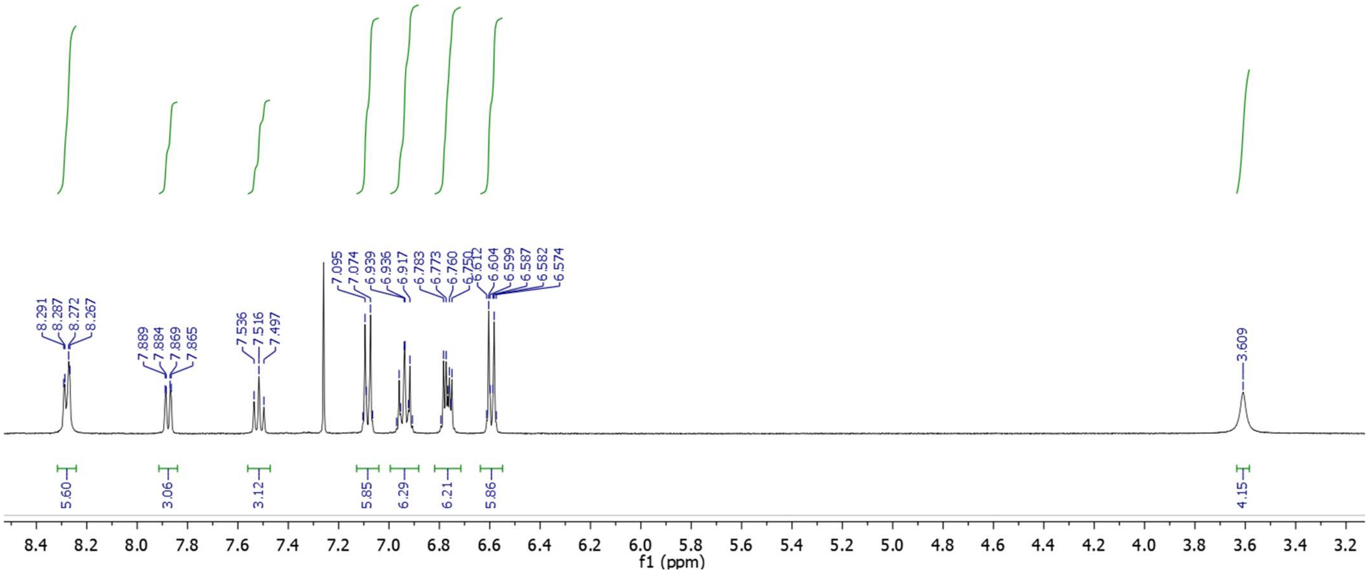

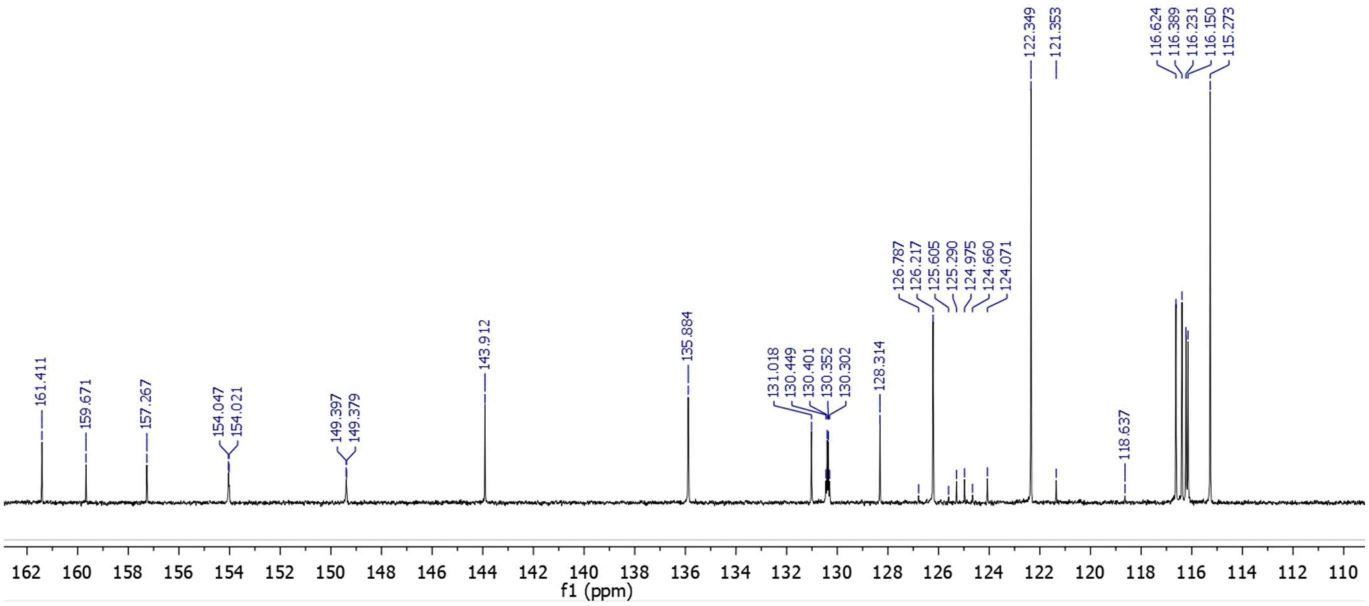

Figure S36. 1H NMR at 400 MHz and 13C NMR at 100 MHz spectra for compound **52**

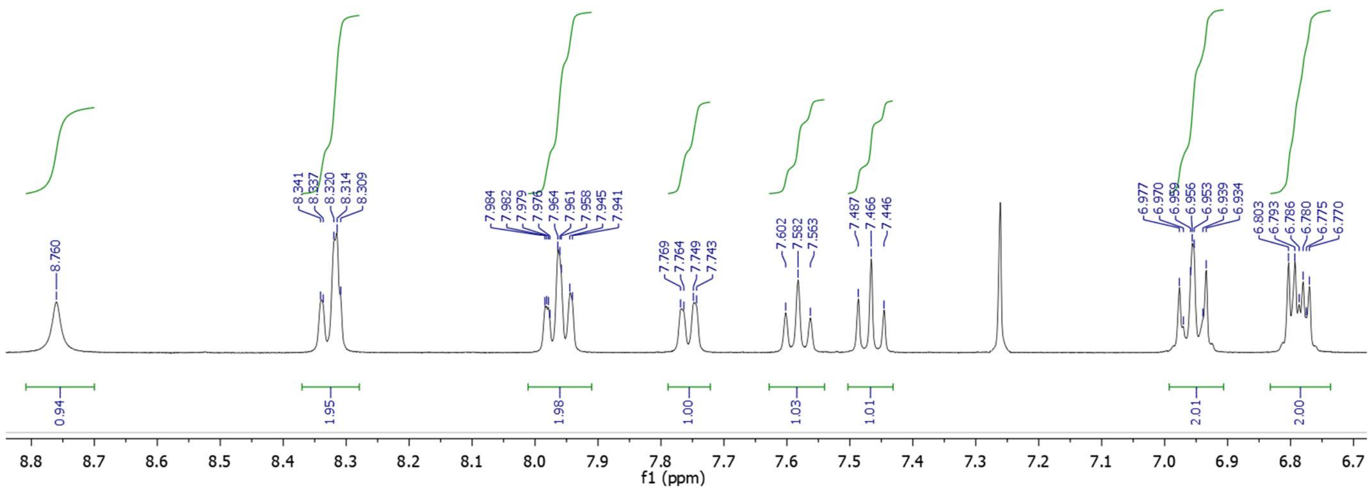

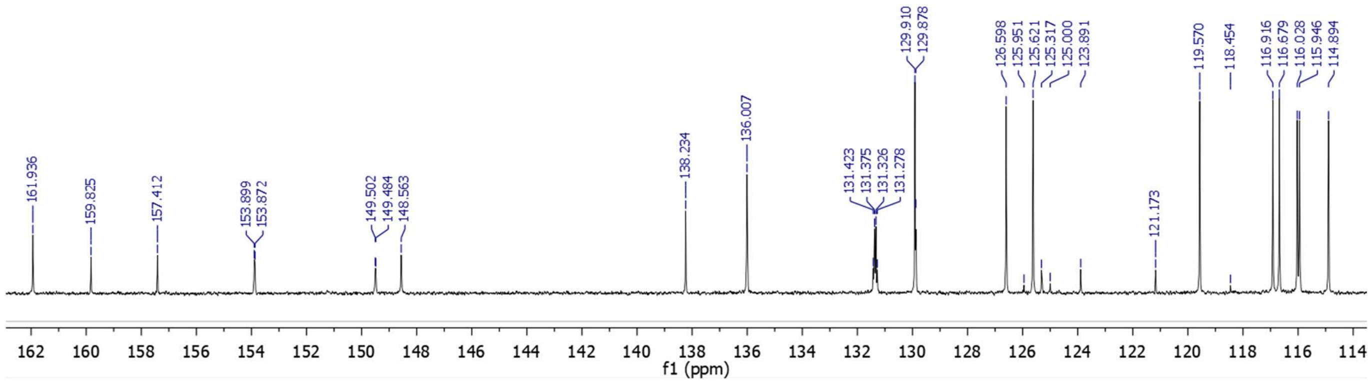

Figure S37. 1H NMR at 400 MHz and 13C NMR at 100 MHz spectra for compound **53**

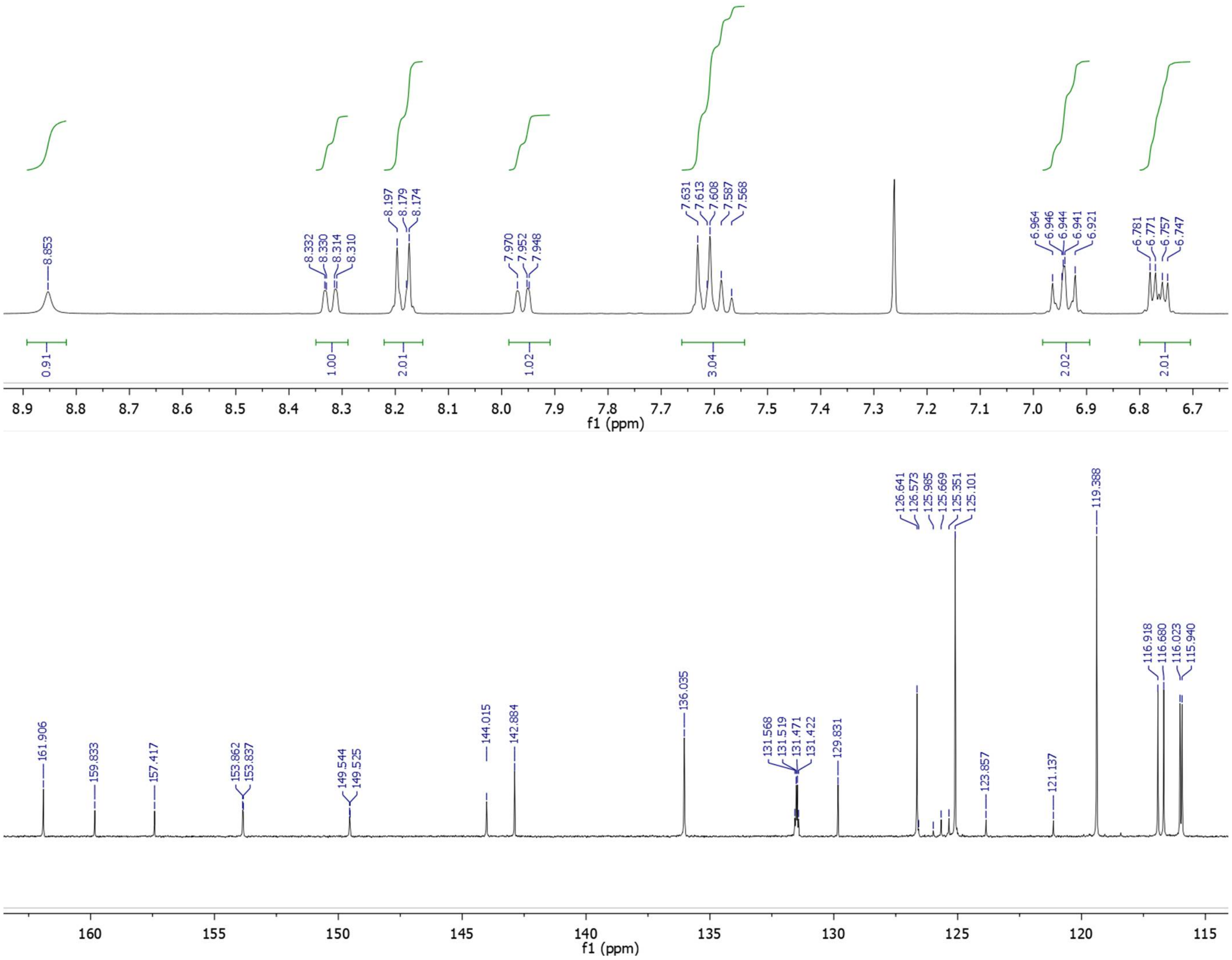

Figure S38. 1H NMR at 400 MHz and 13C NMR at 100 MHz spectra for compound **54**

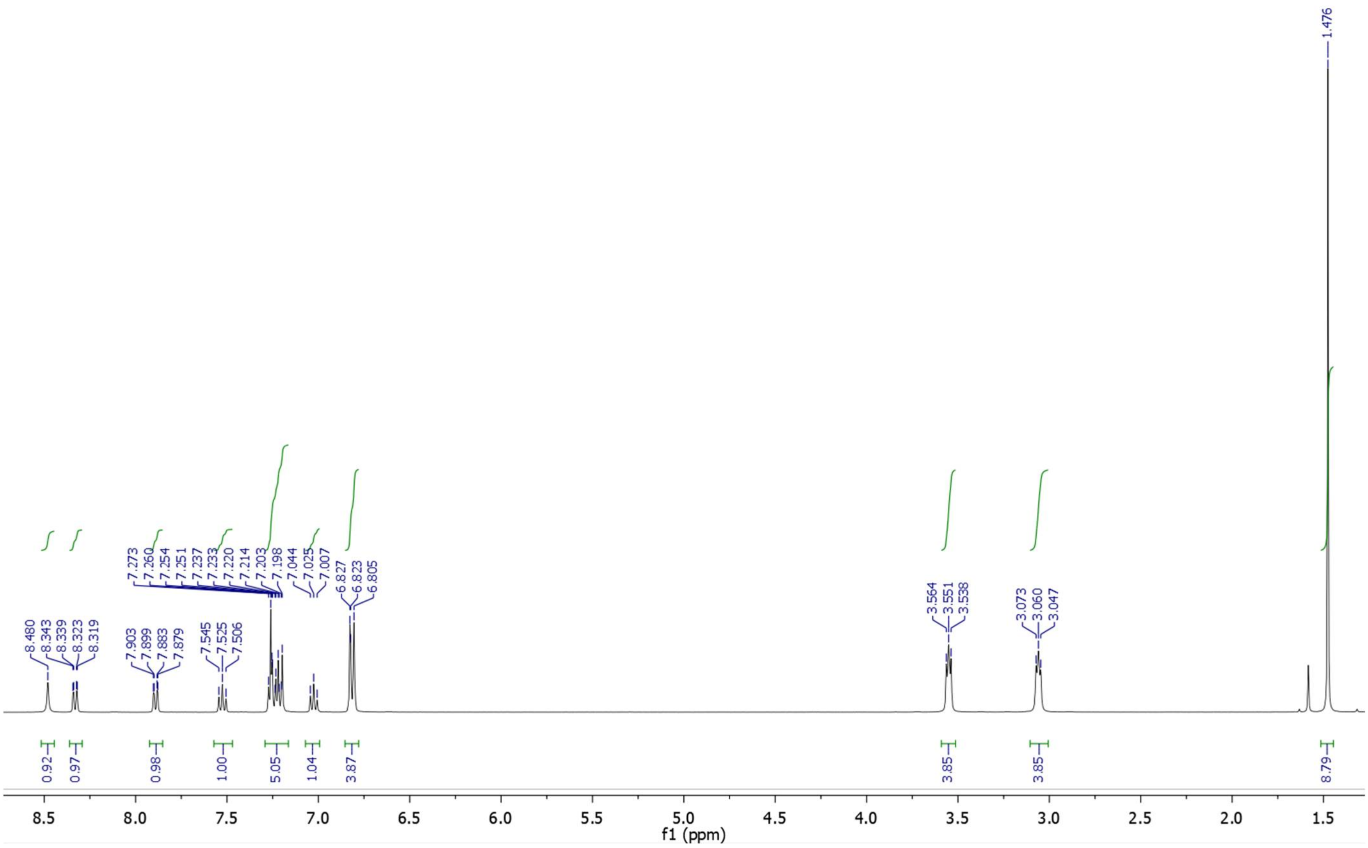

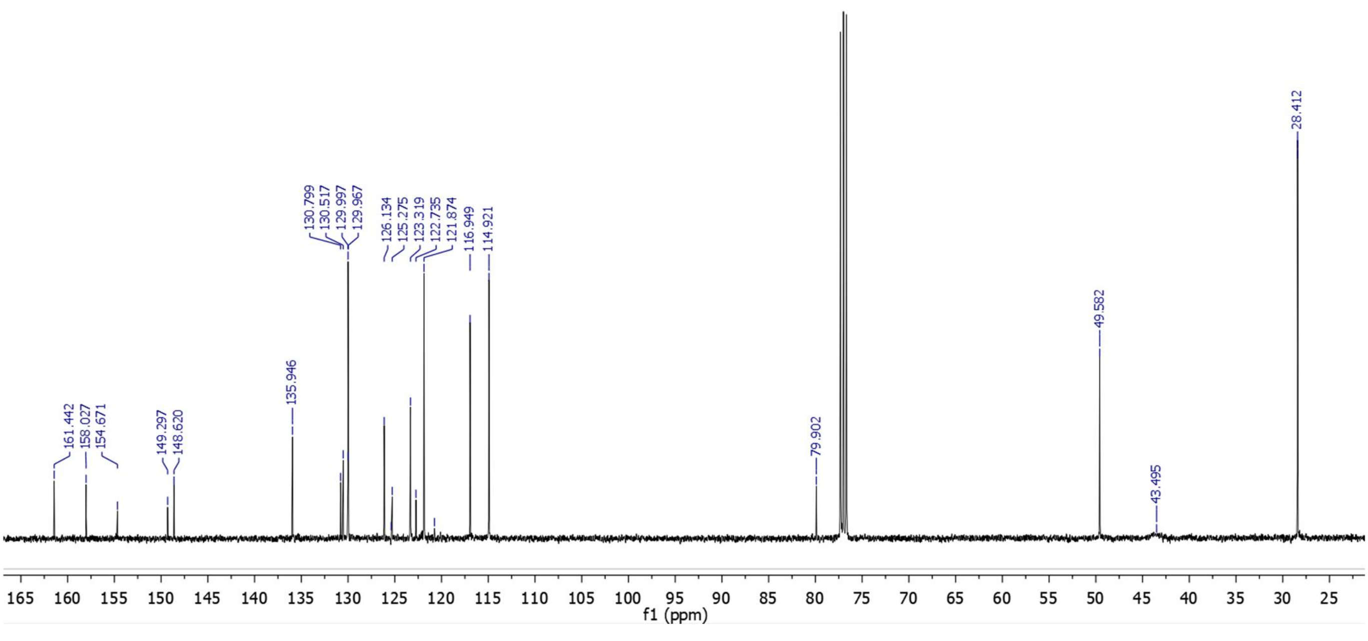

Figure S39. 1H NMR at 400 MHz and 13C NMR at 100 MHz spectra for compound **55**

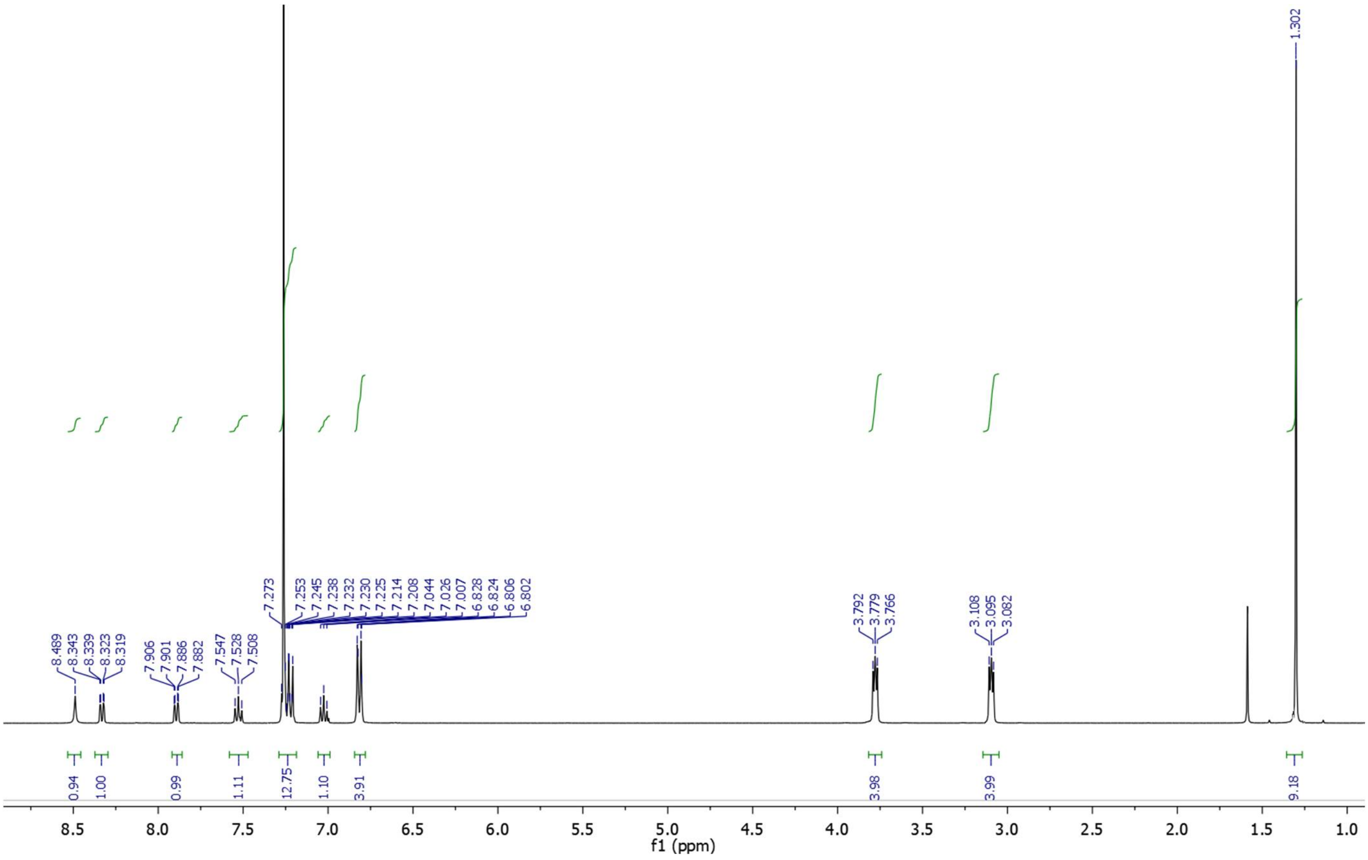

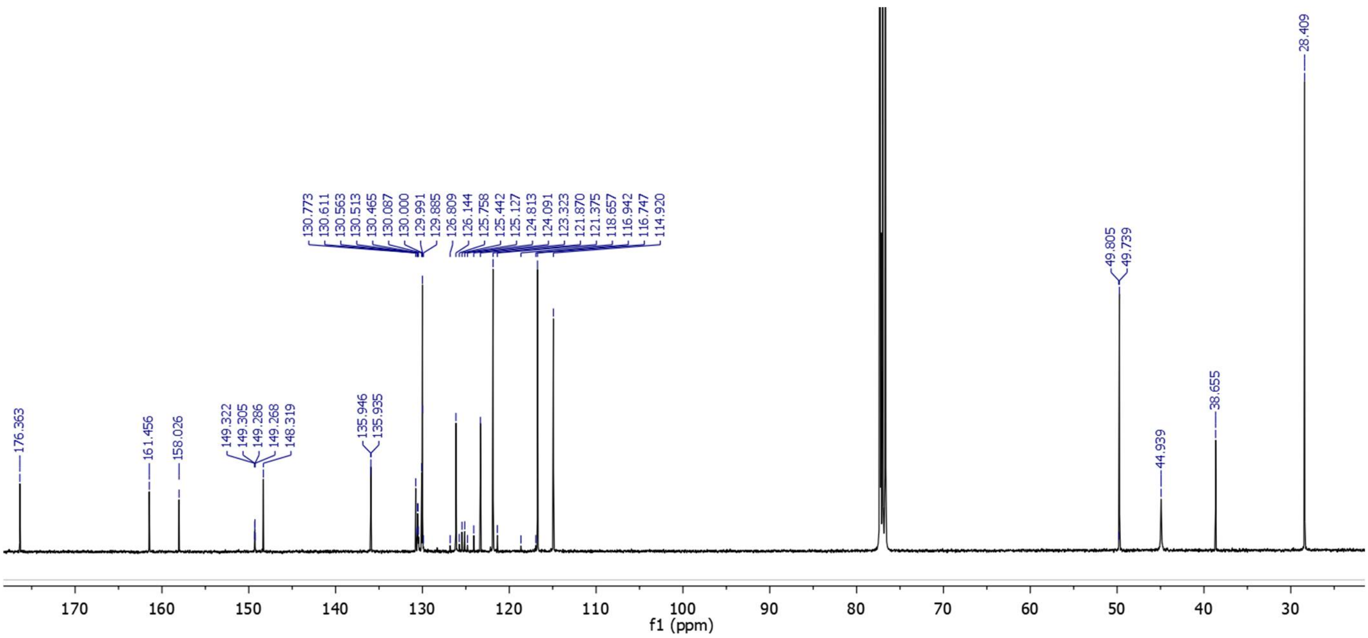

Figure S40. 1H NMR at 400 MHz and 13C NMR at 100 MHz spectra for compound **56**

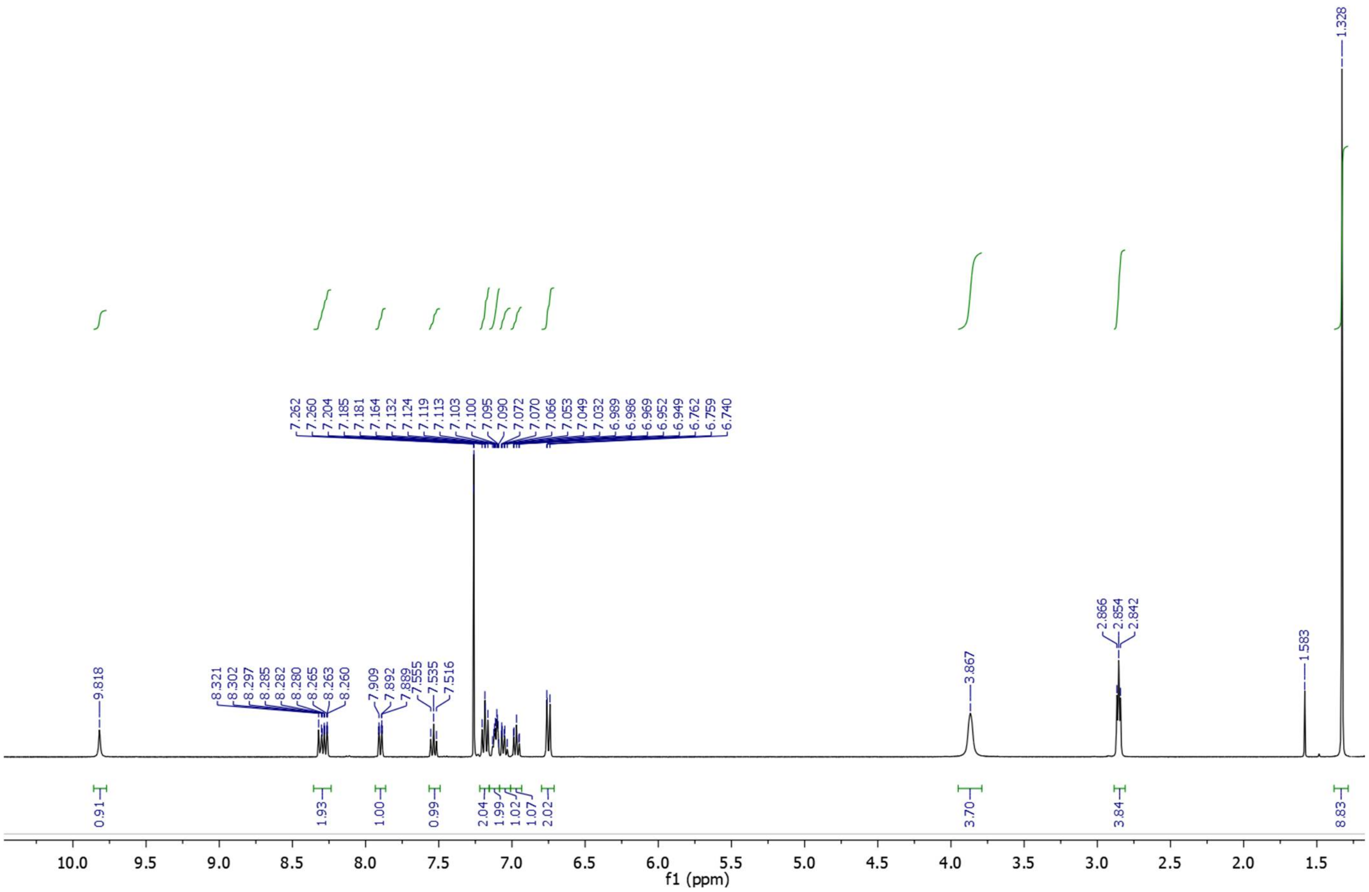

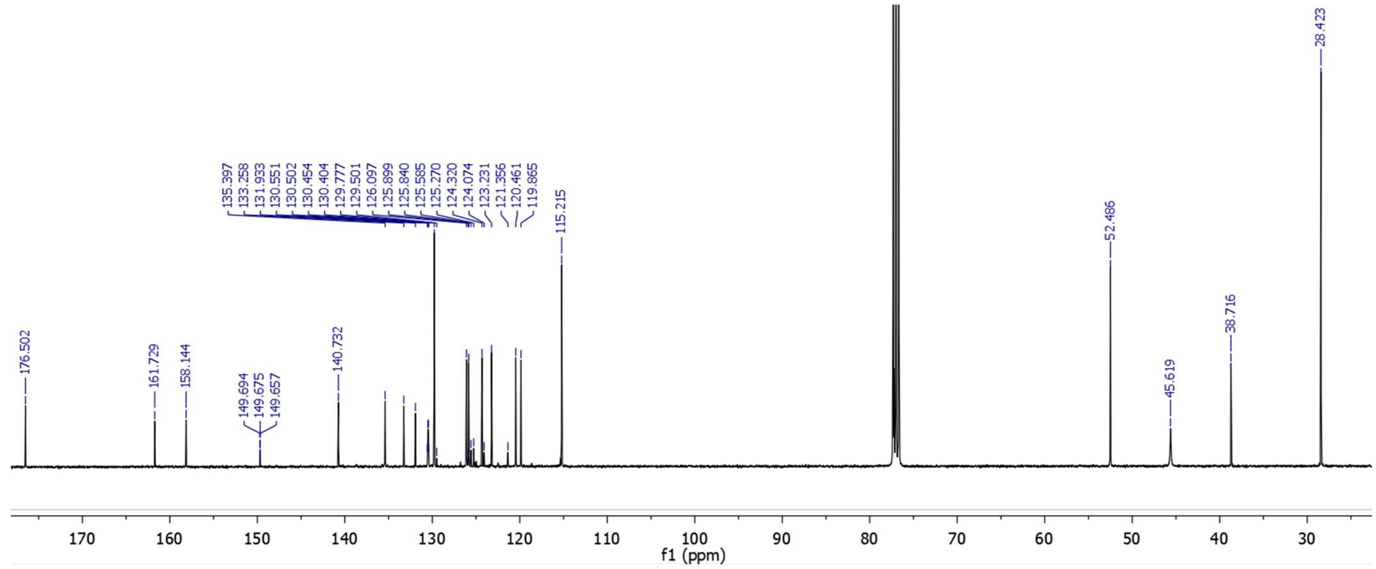

Supplement: Supplementary file 1 [file pharmaceuticals-14-01109-s001.zip › pharmaceuticals-1409769-supplementary.pdf]
